# Supplementary figures and images for: Learning protein constitutive motifs from sequence data
Source: eLife. 2019 Mar 12;8:e39397. doi: 10.7554/eLife.39397 (PMC6436896; doi:10.7554/eLife.39397)

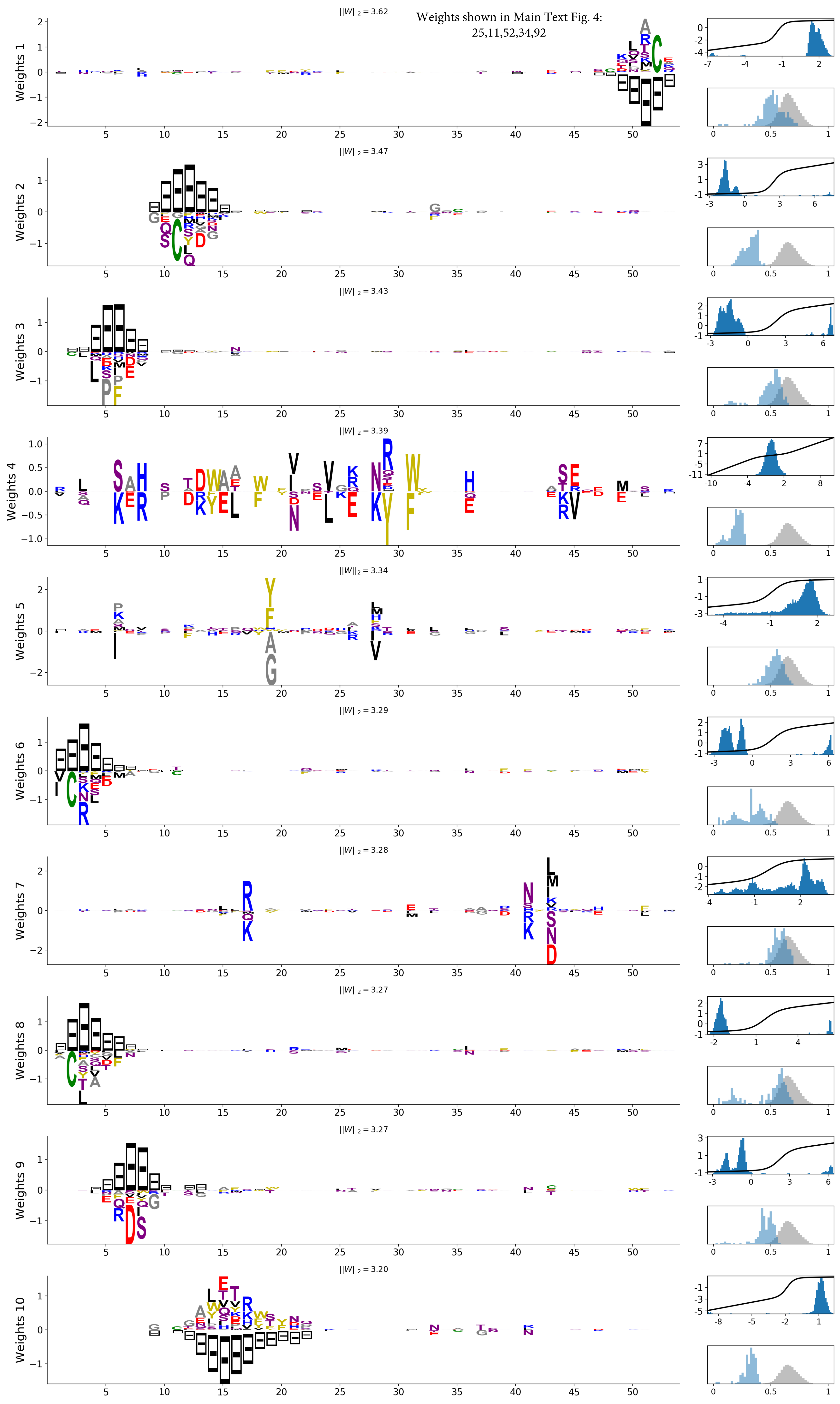

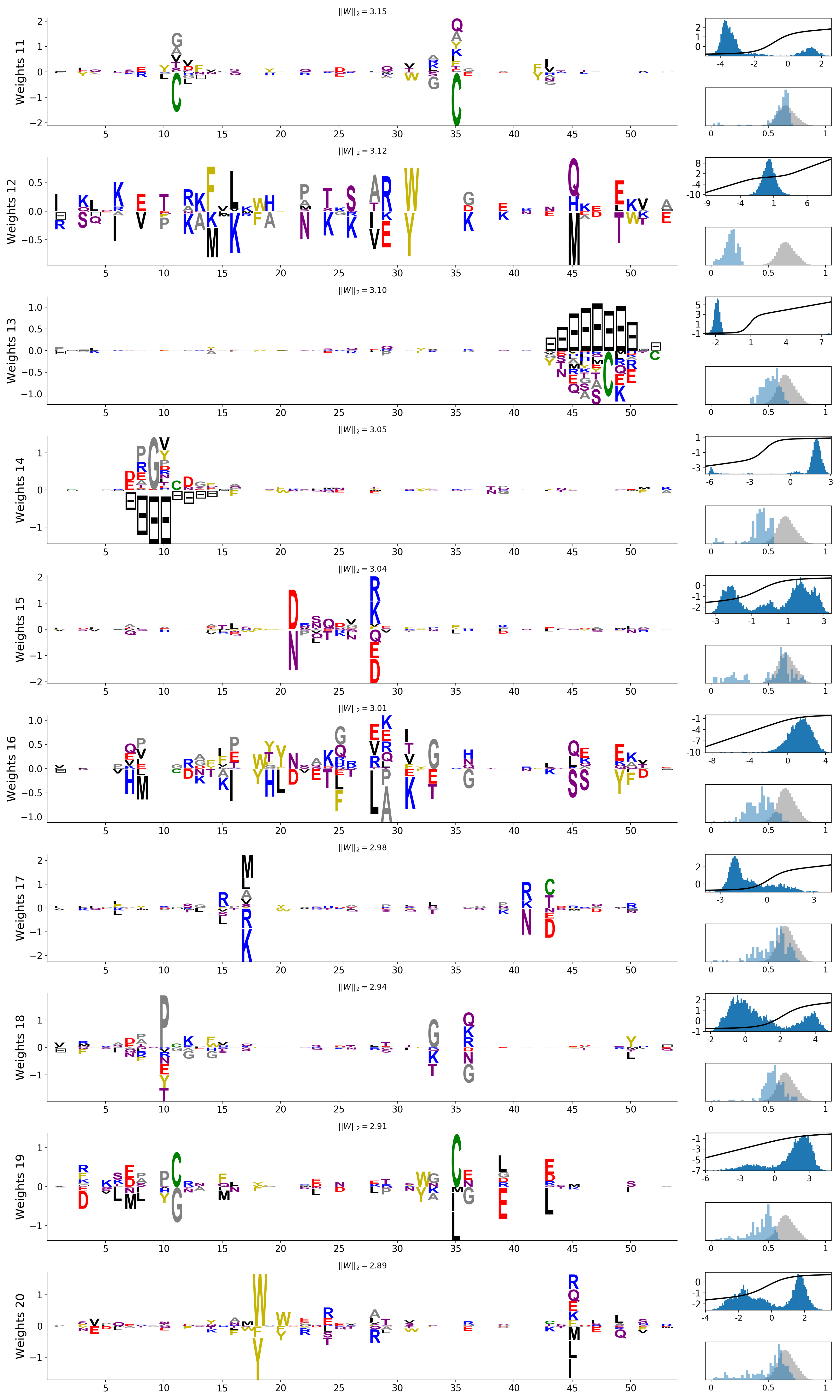

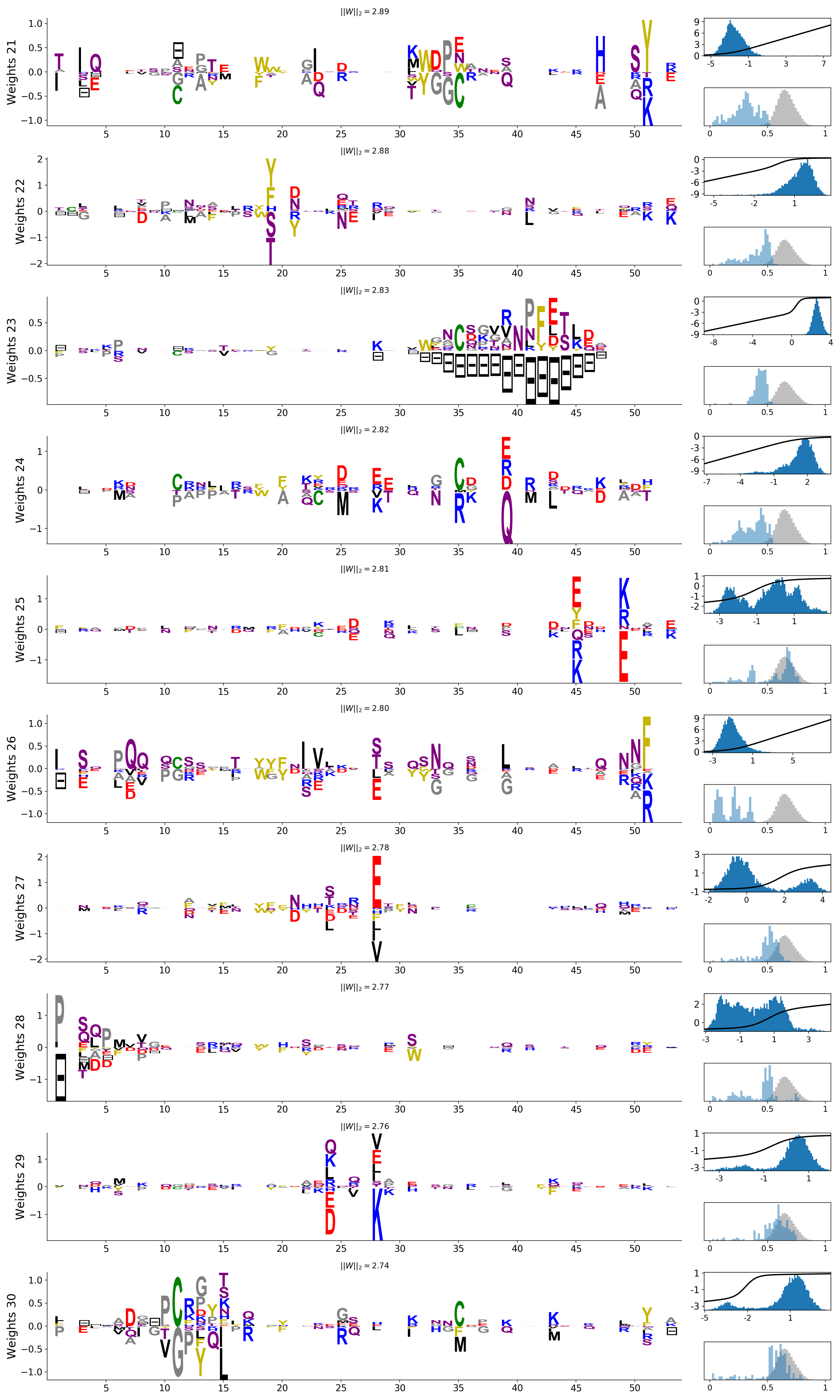

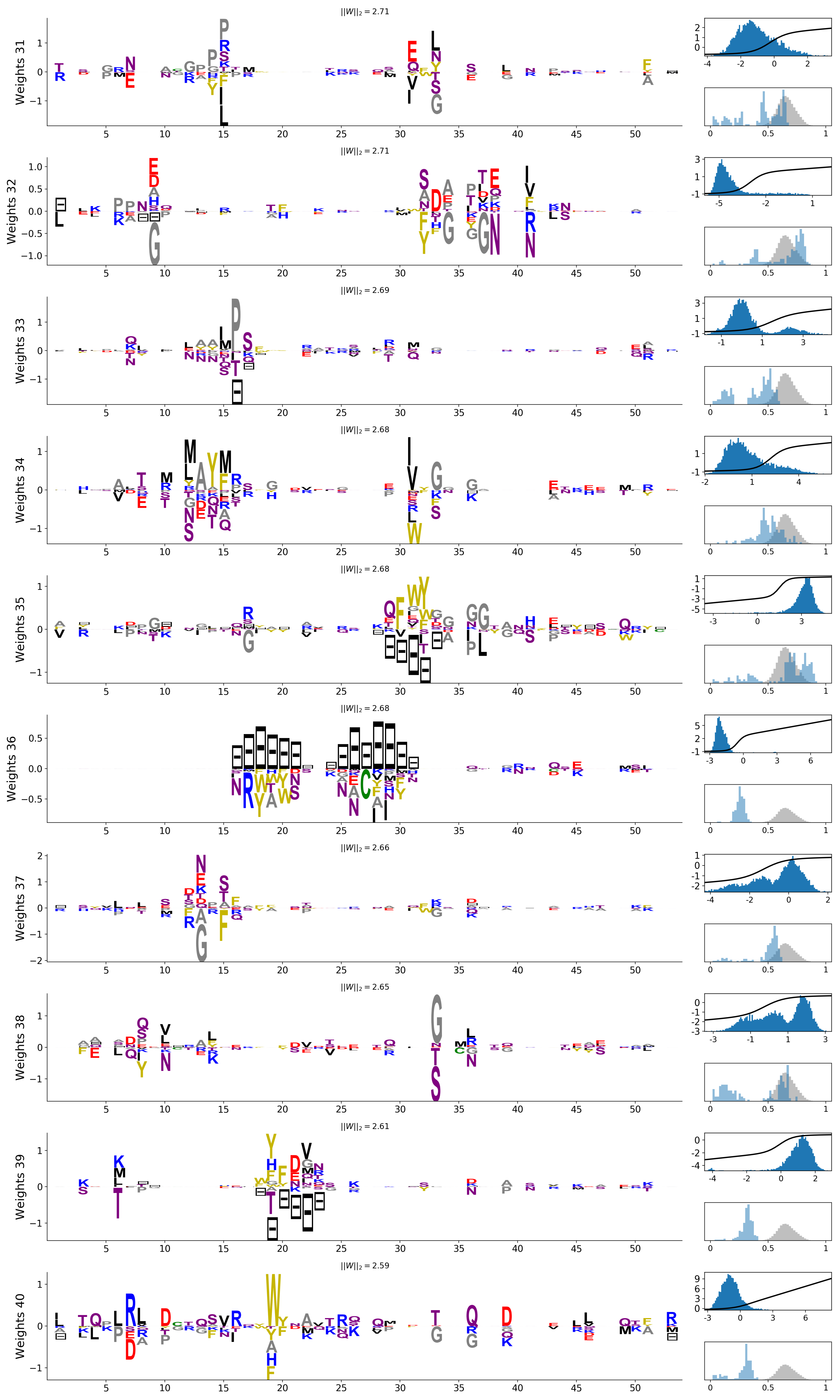

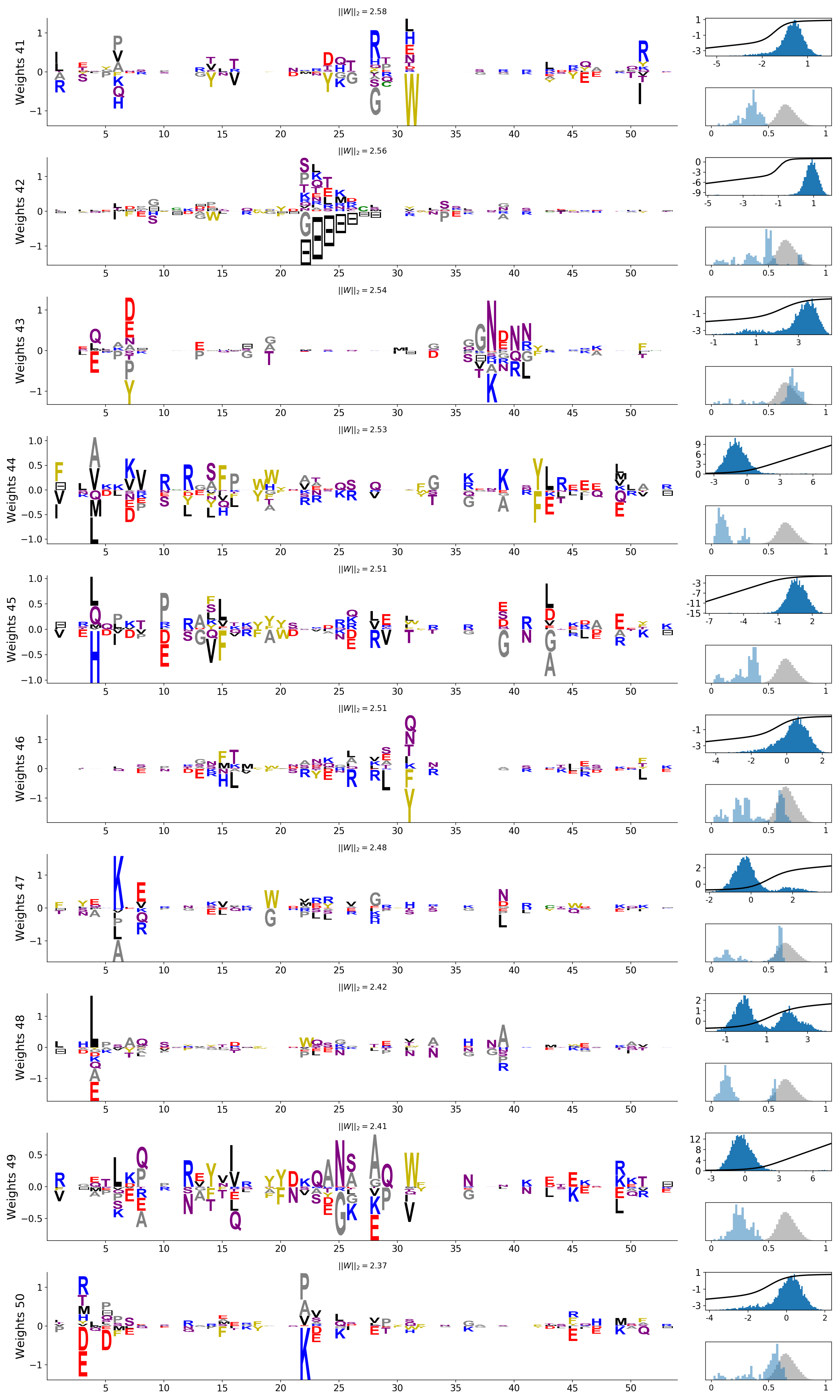

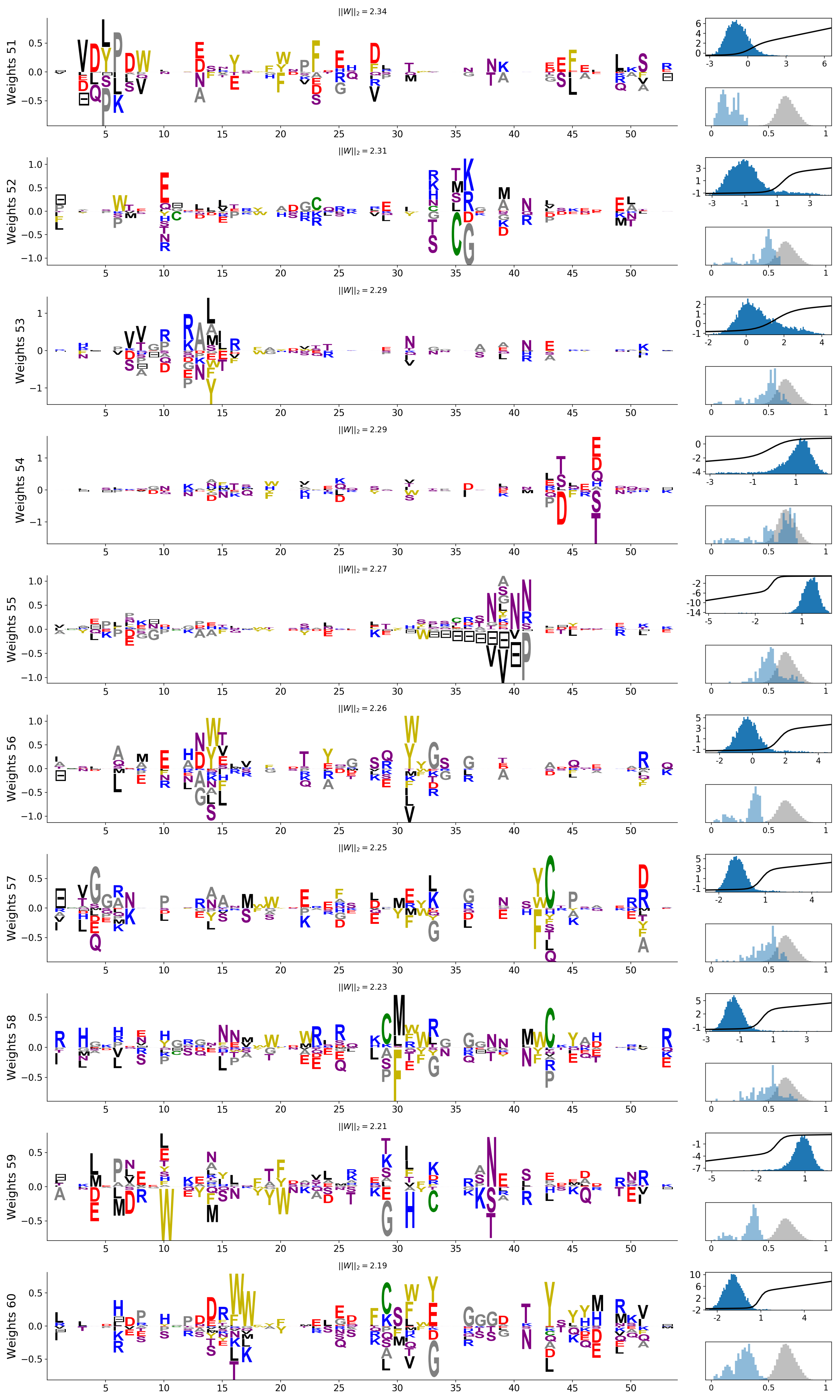

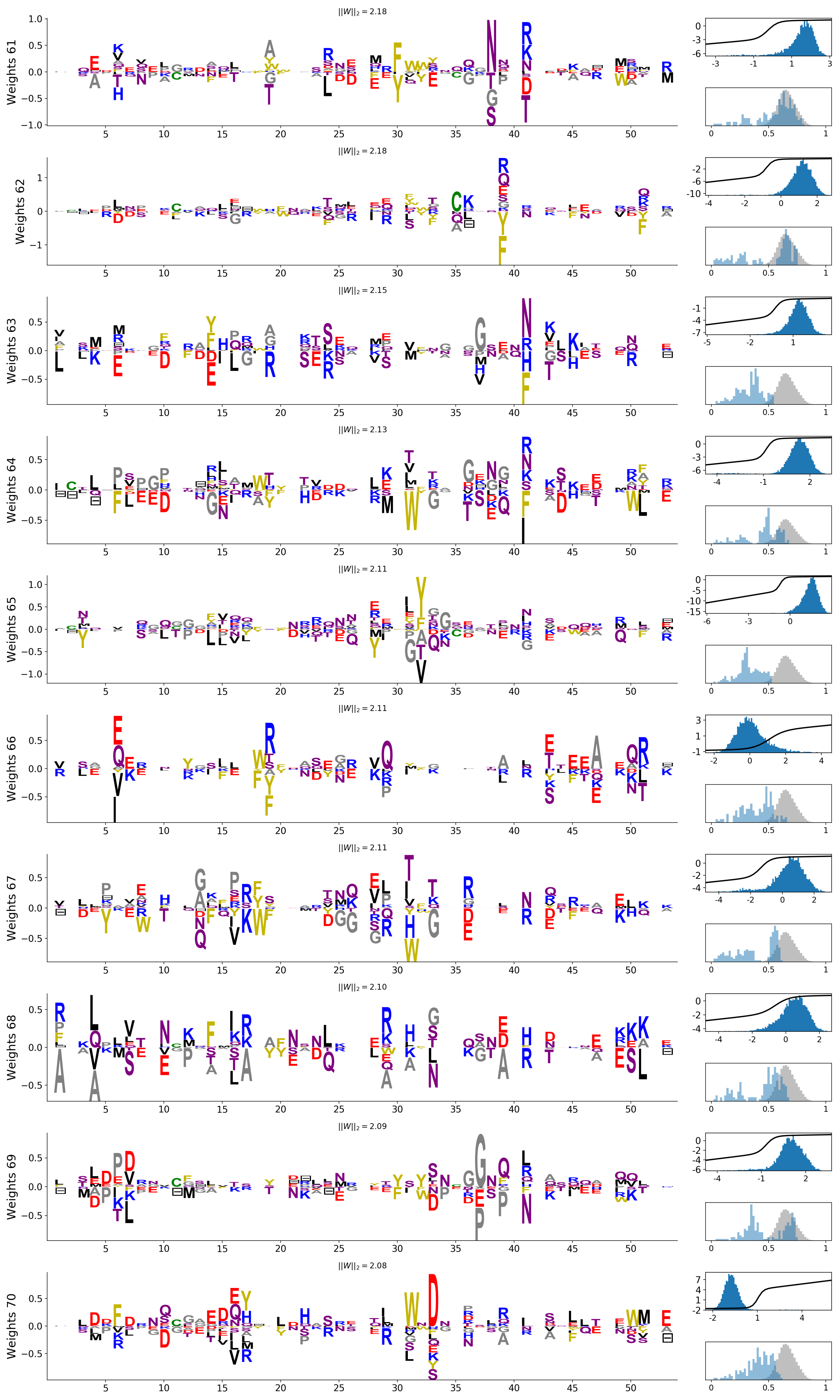

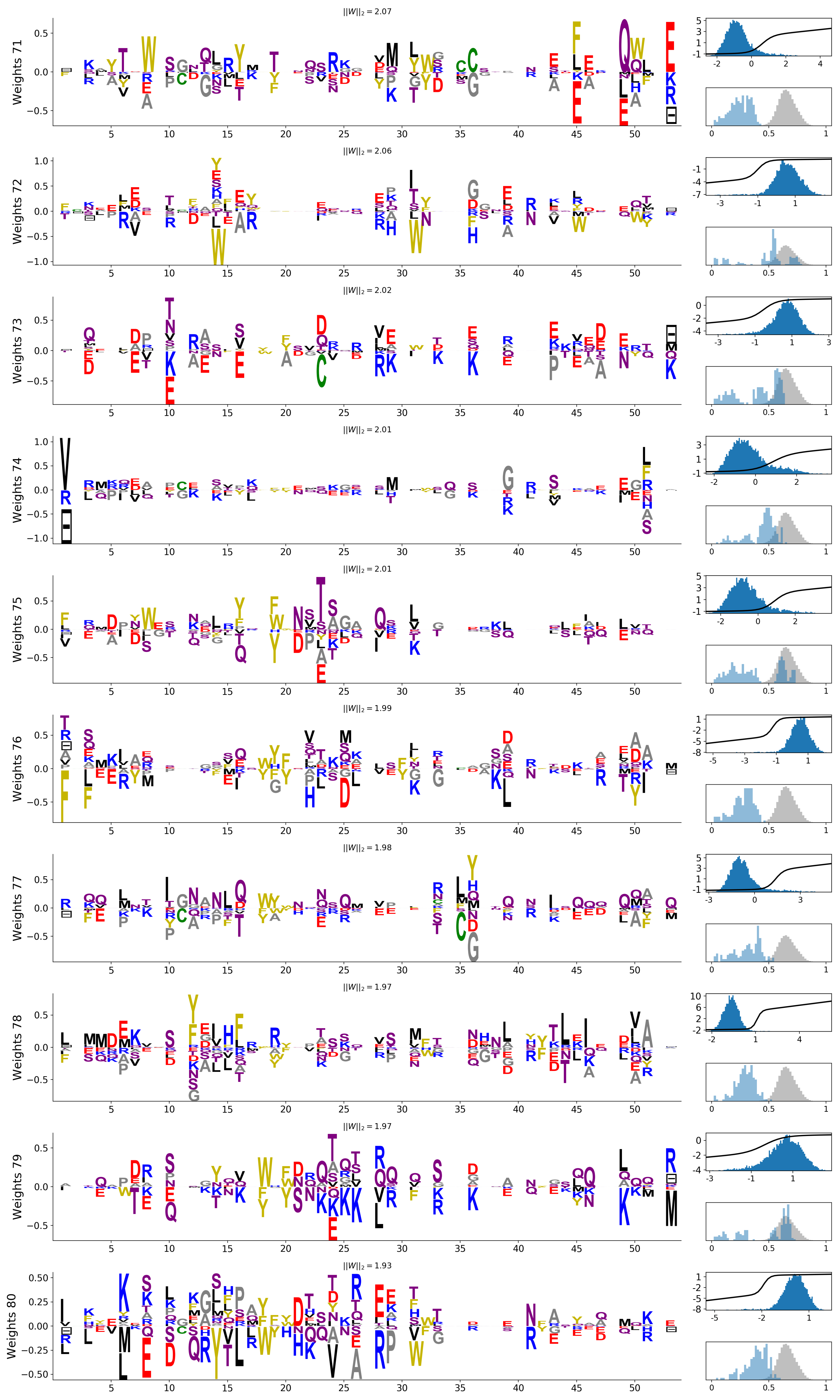

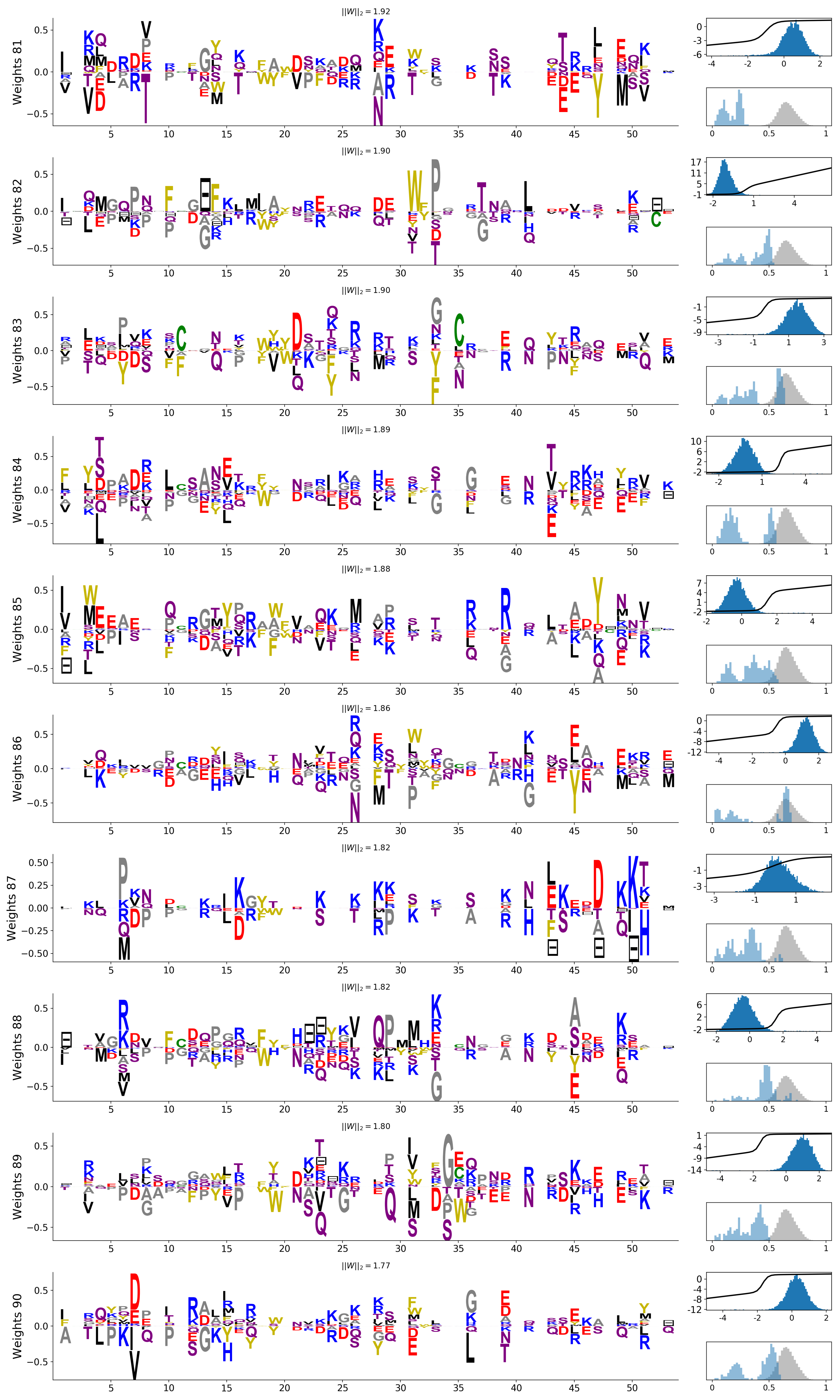

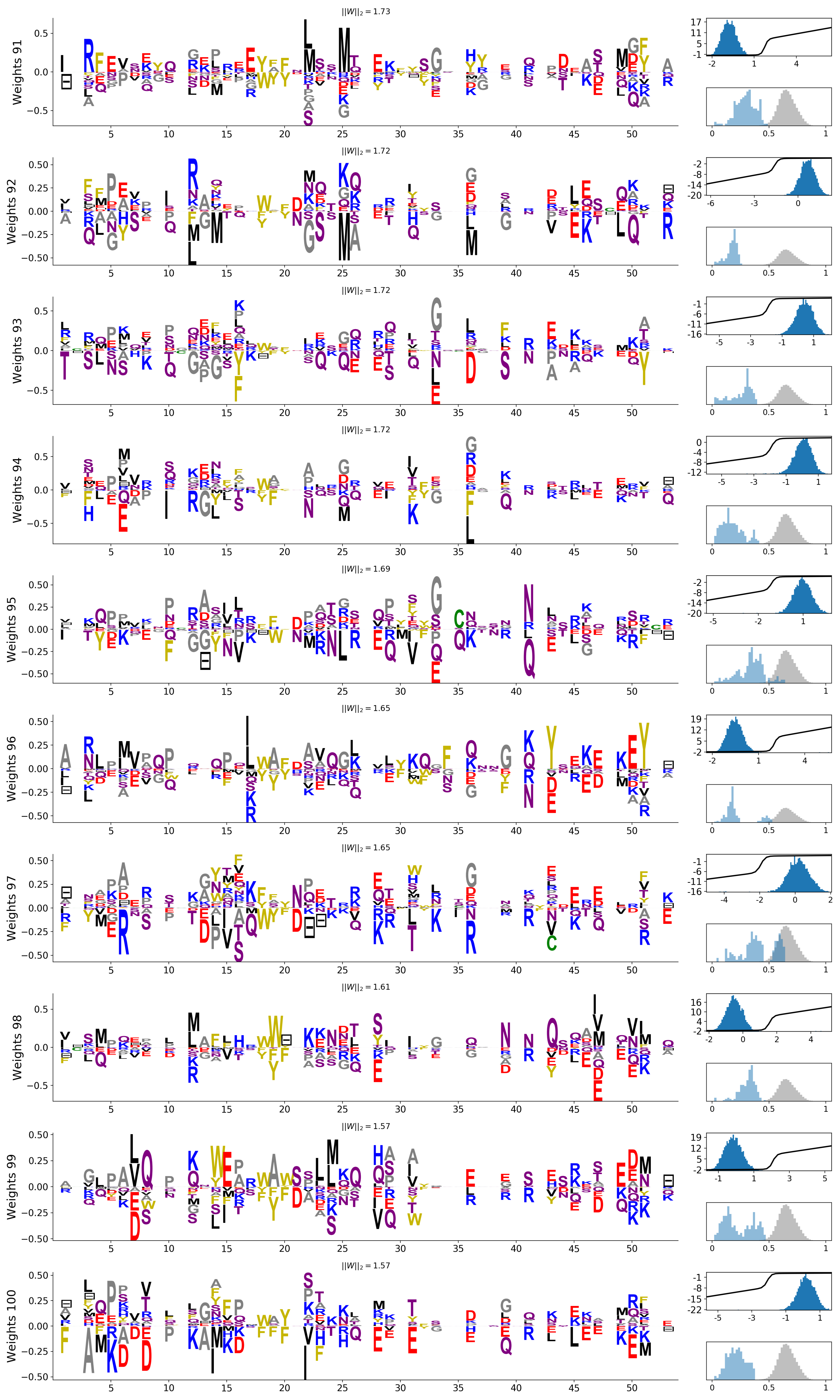

Supplement: Supplementary file 1. [file elife-39397-supp1.pdf]

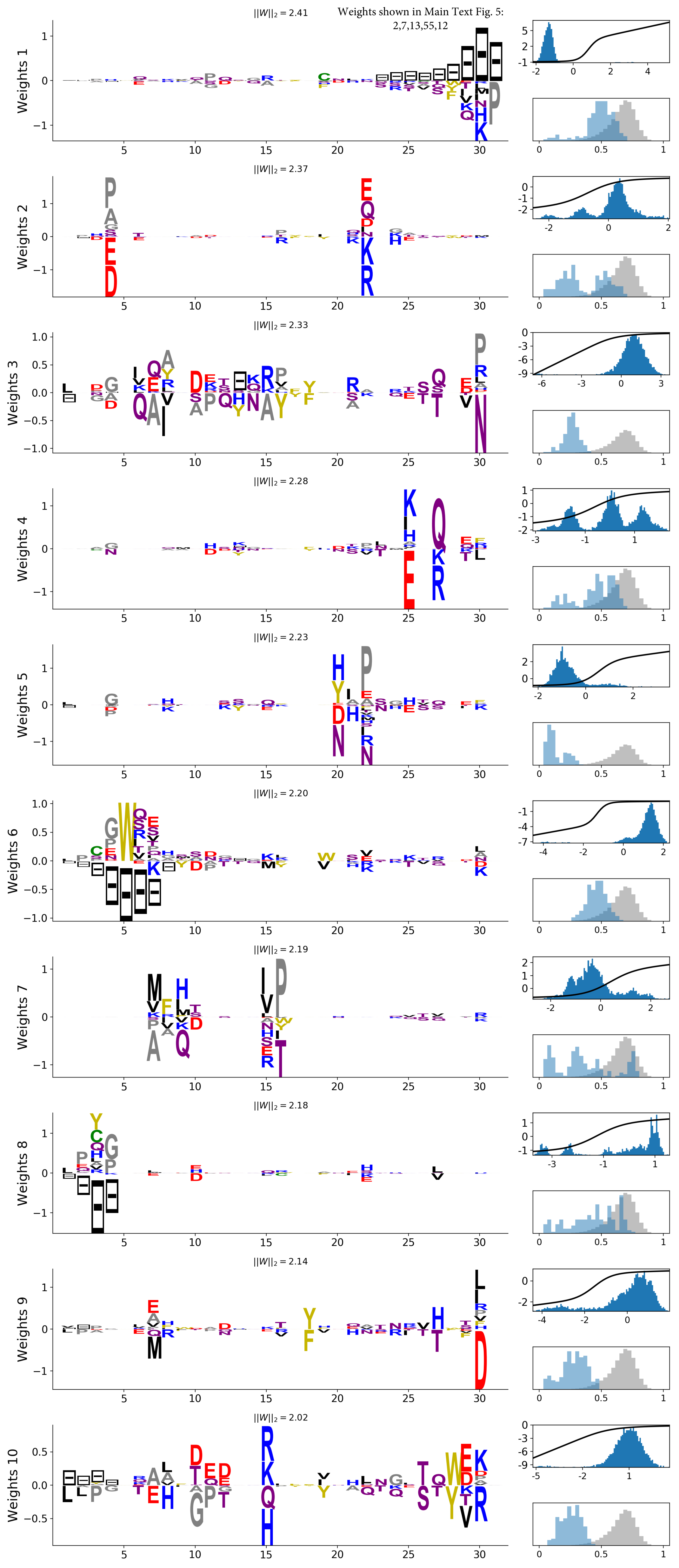

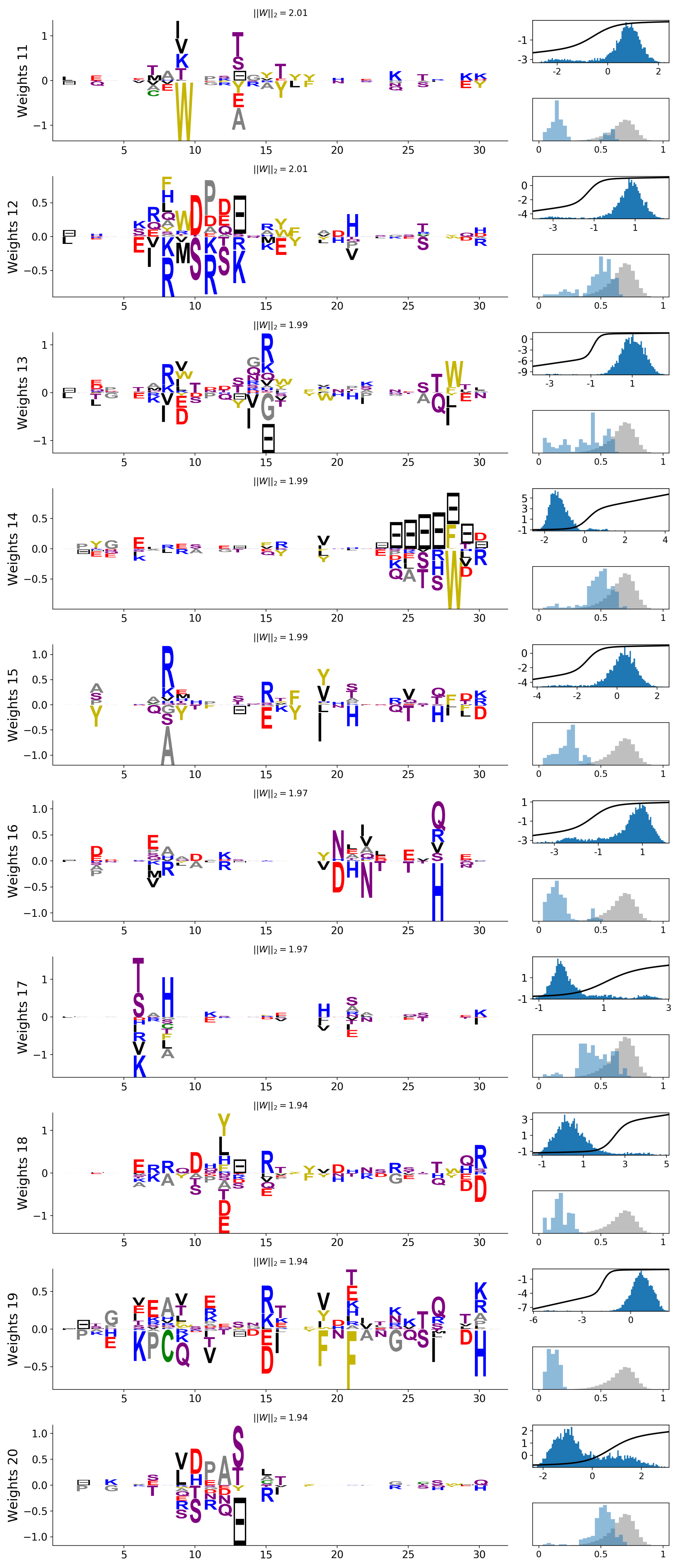

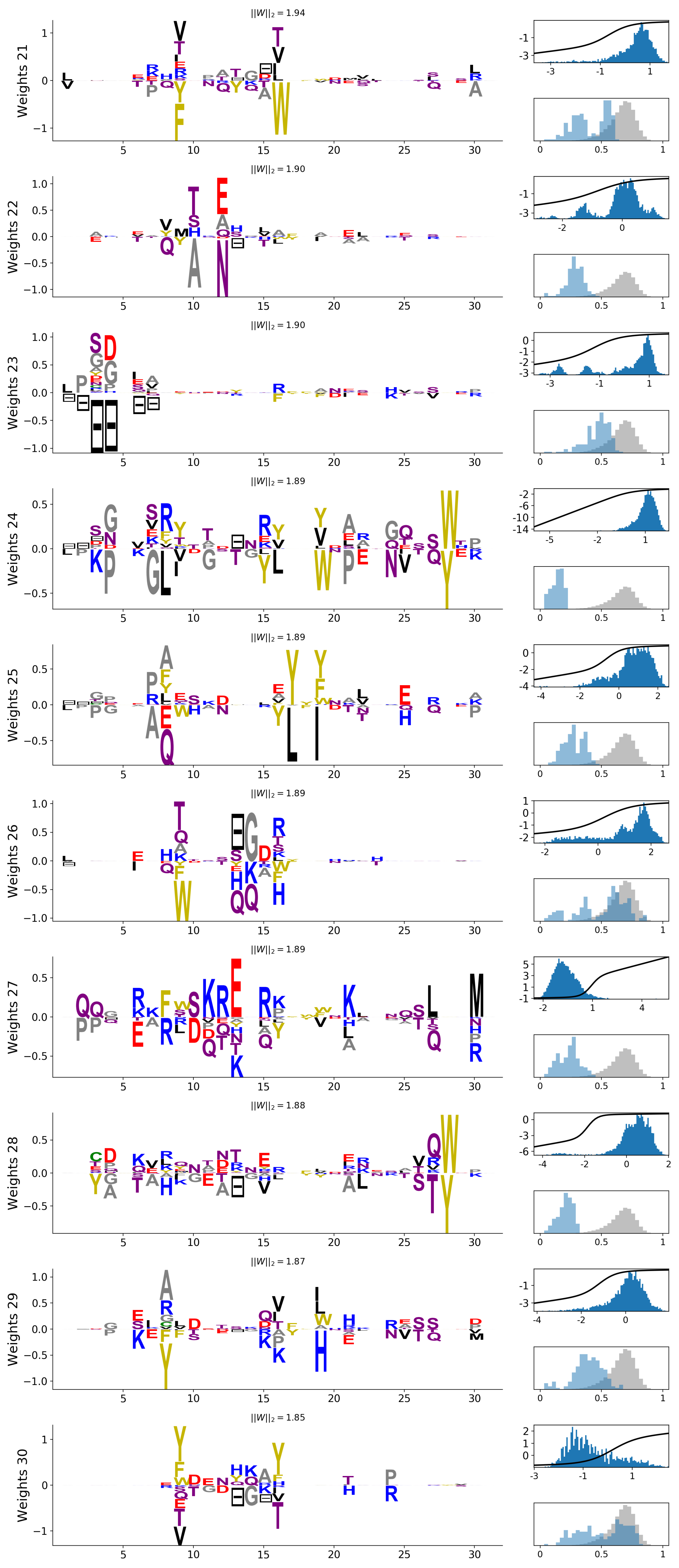

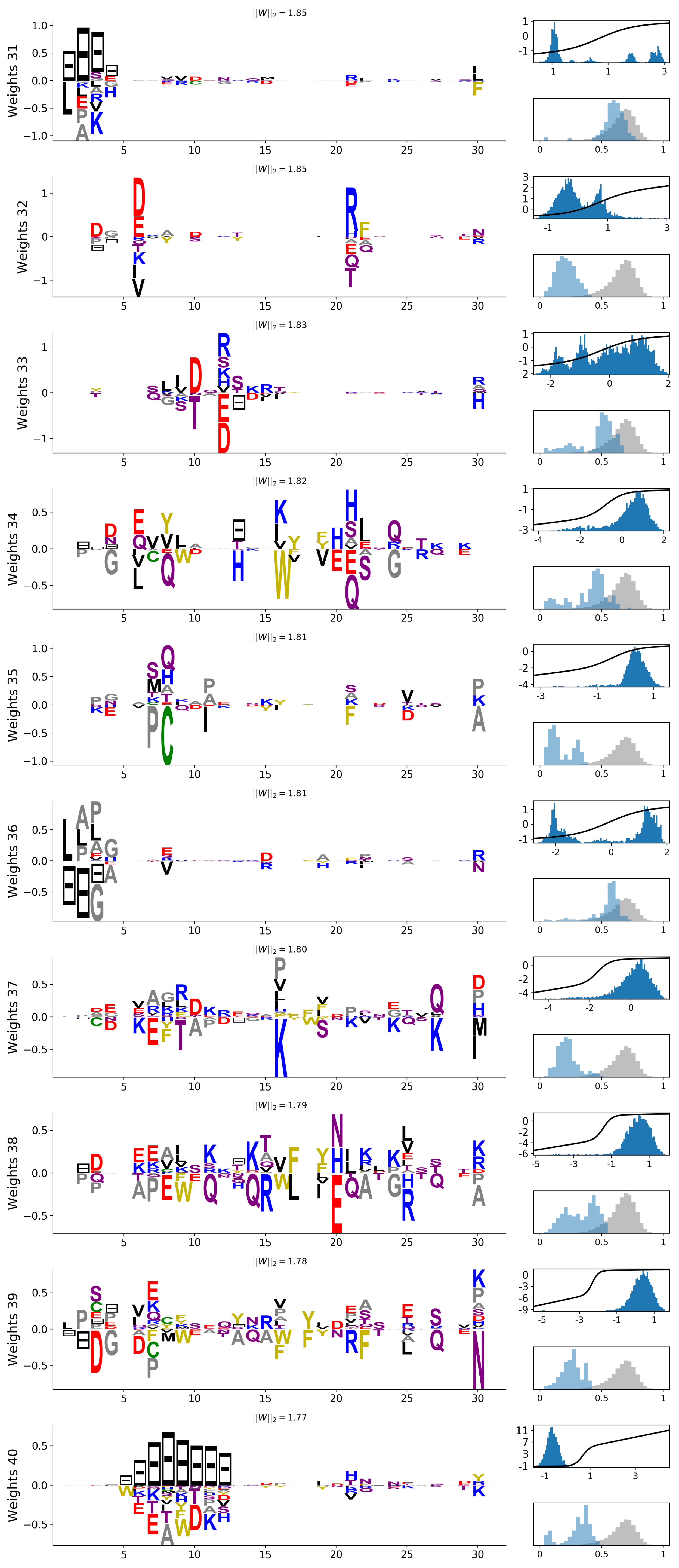

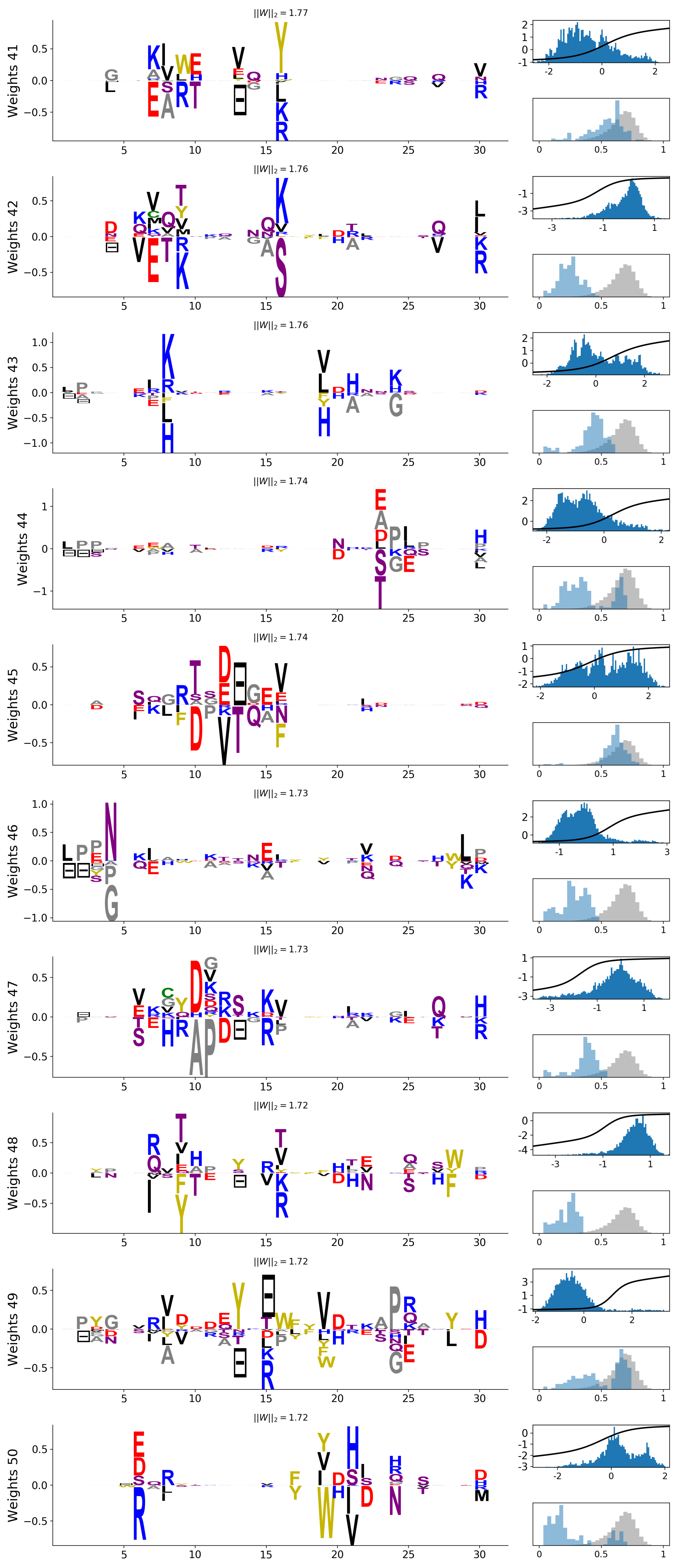

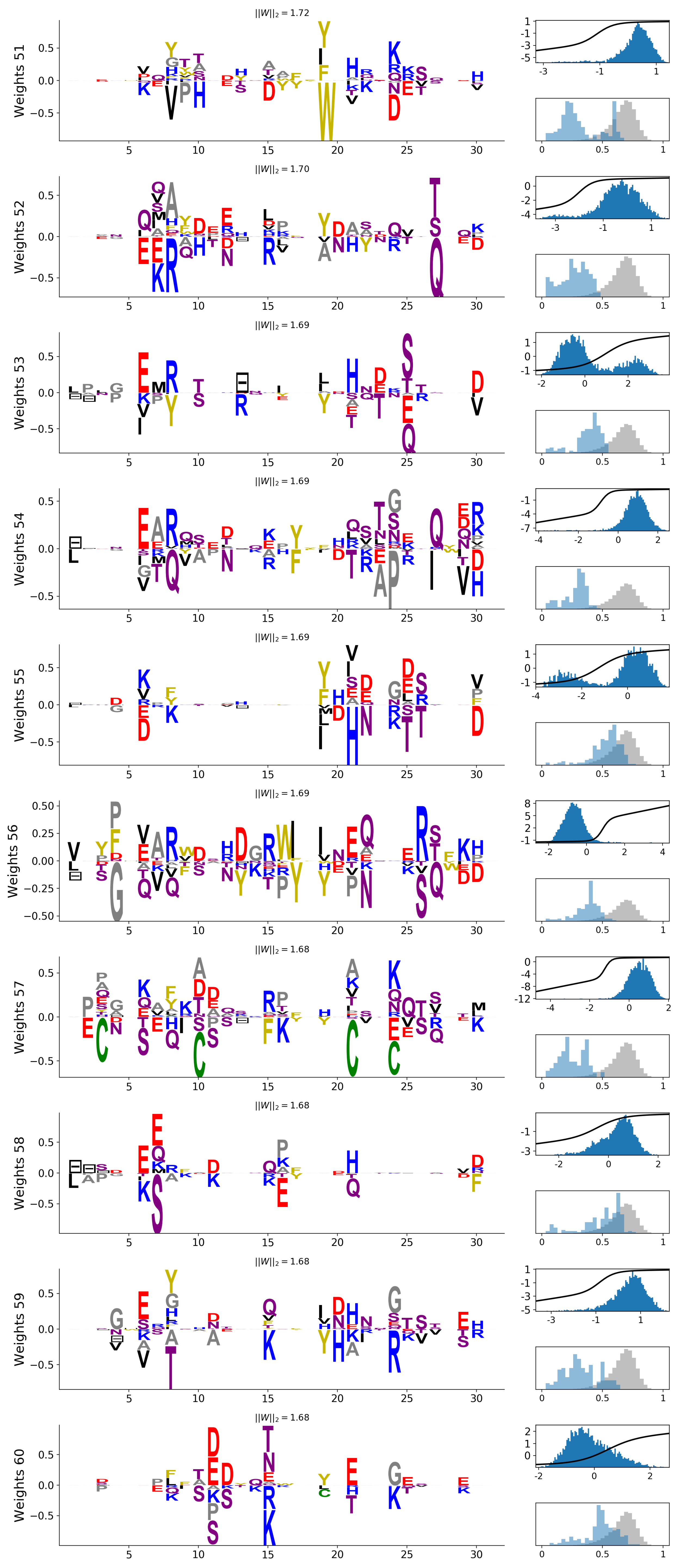

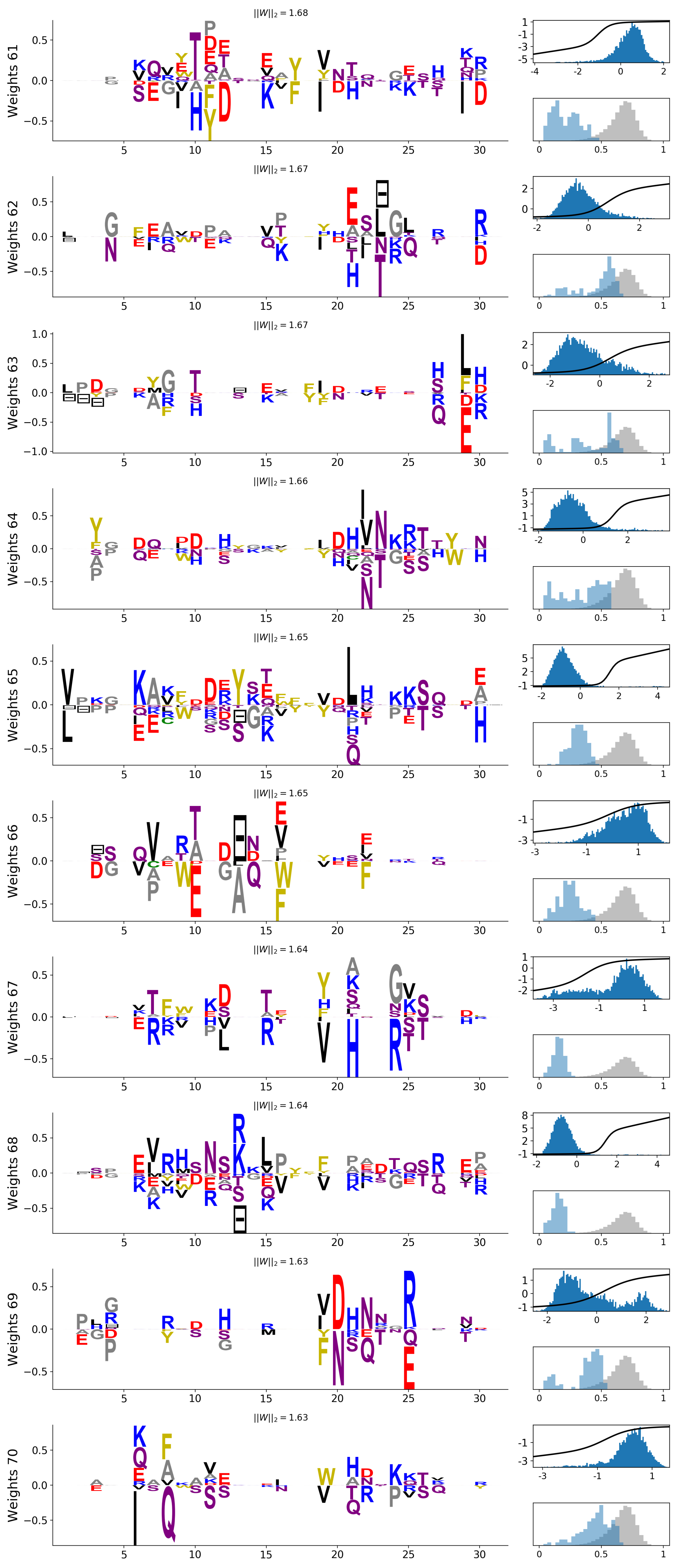

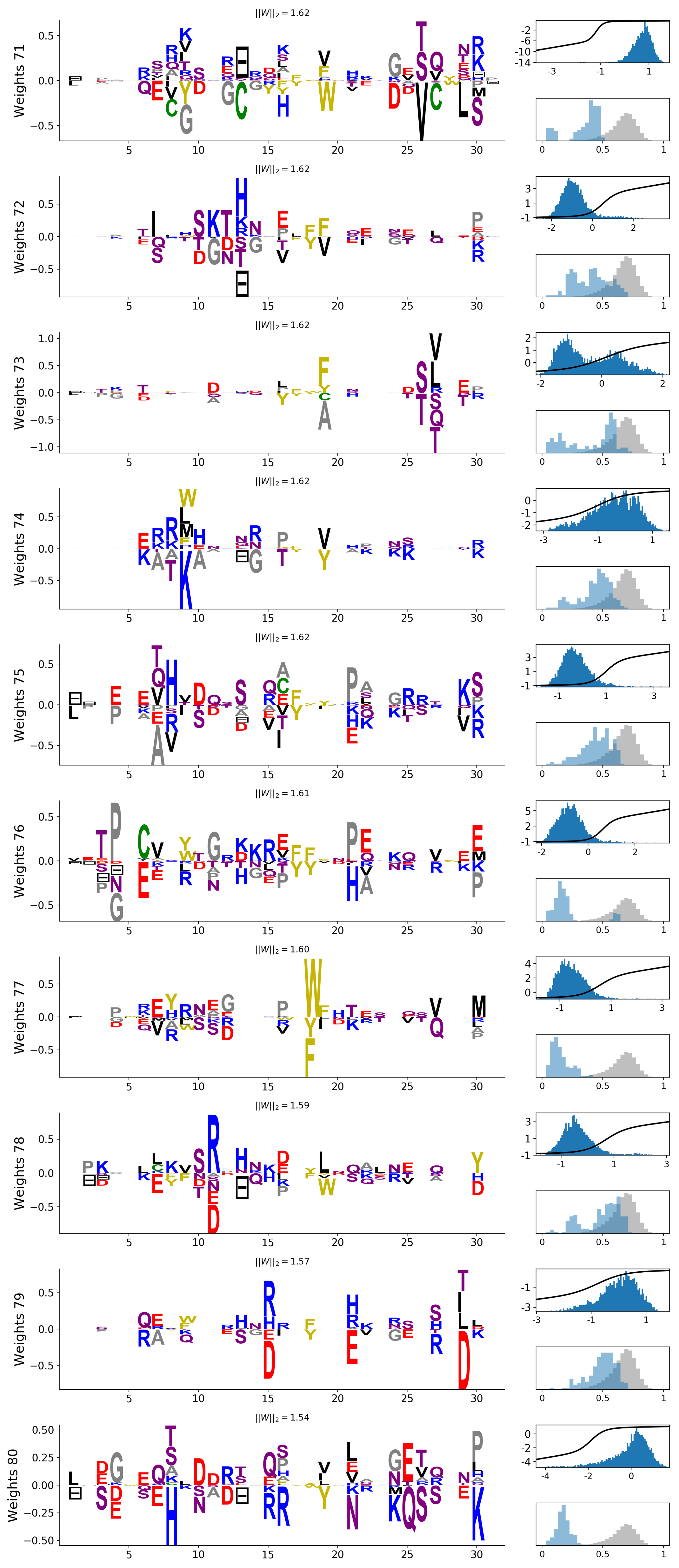

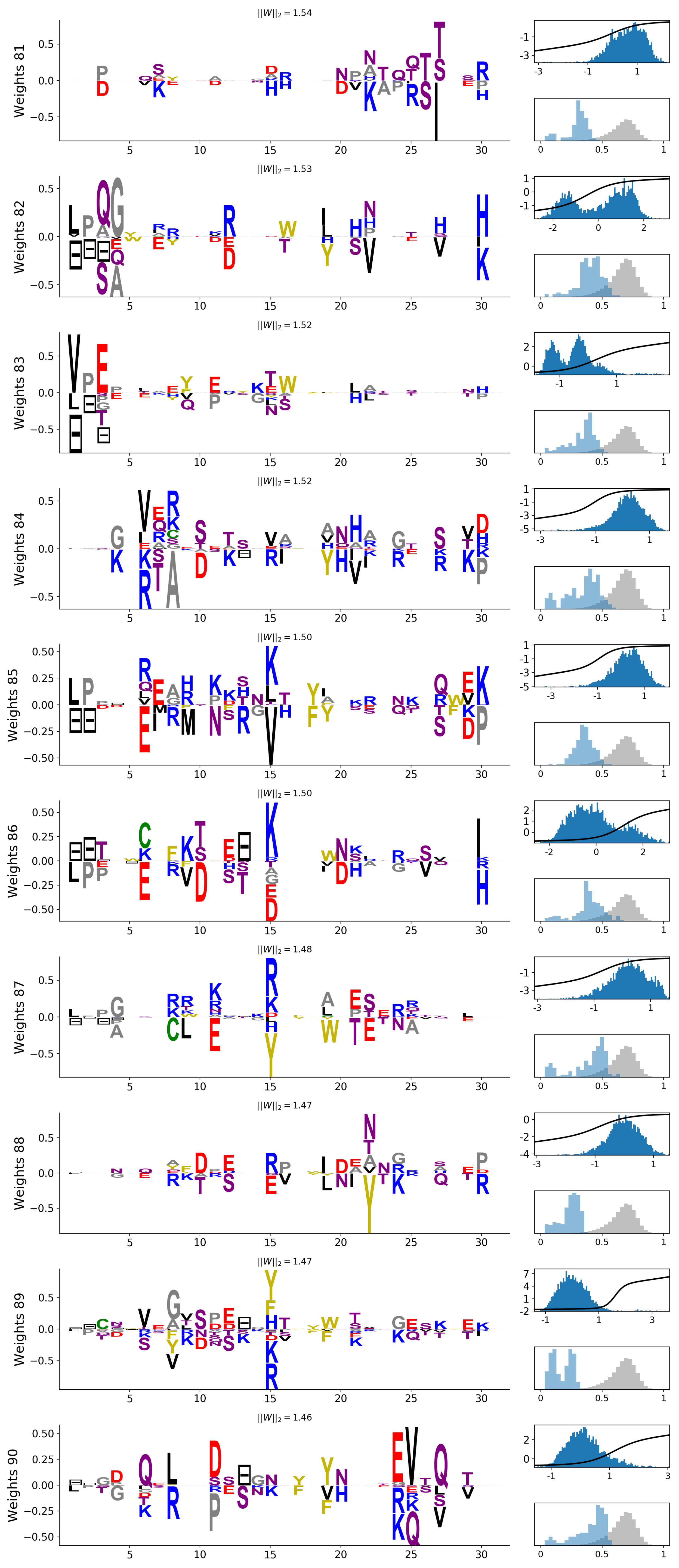

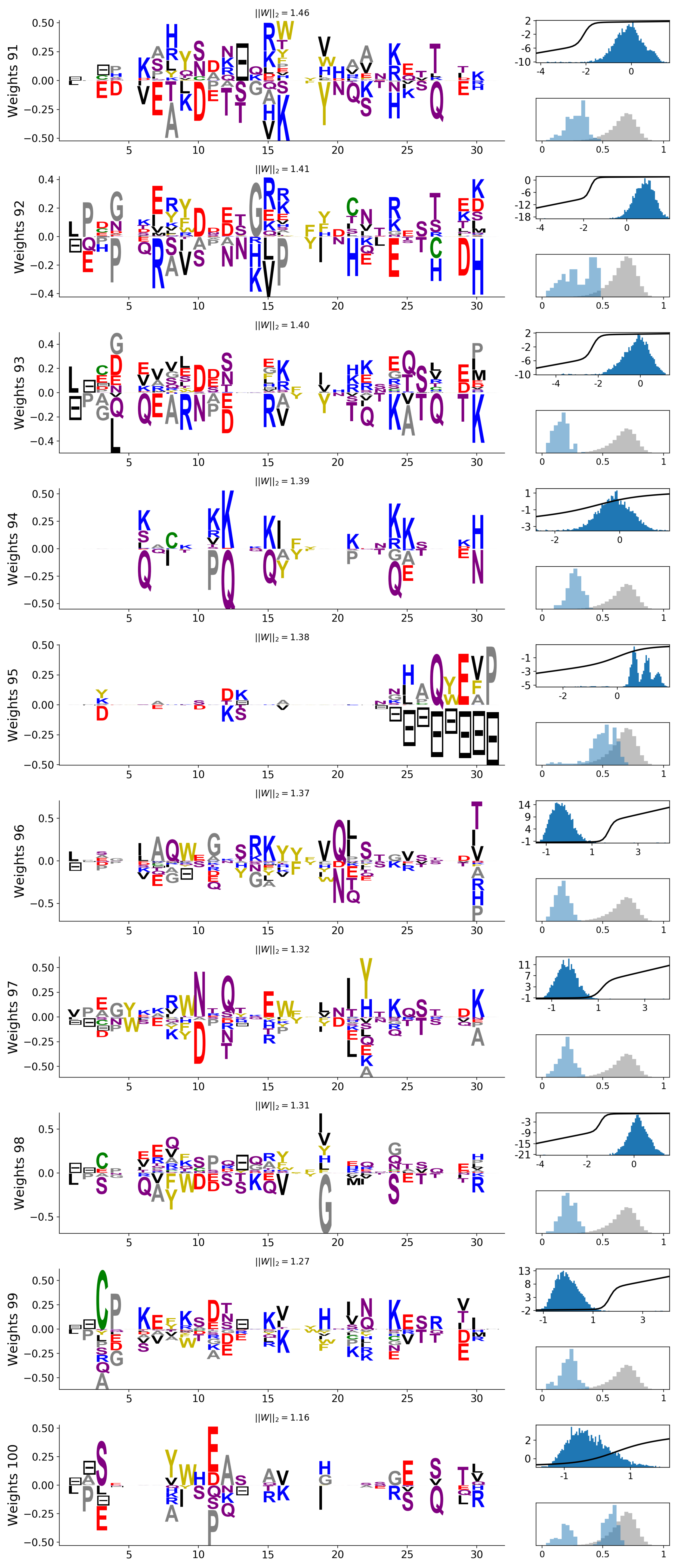

Supplement: Supplementary file 2. [file elife-39397-supp2.pdf]

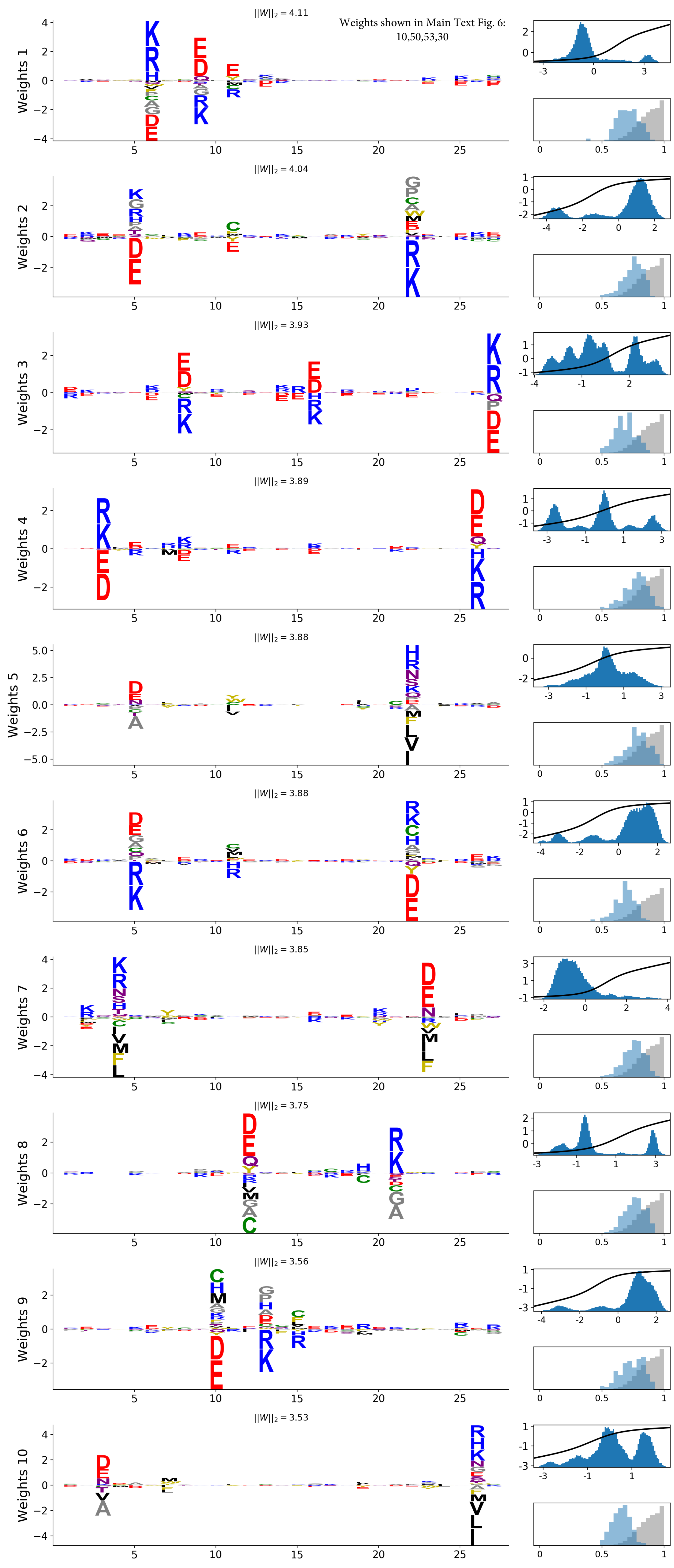

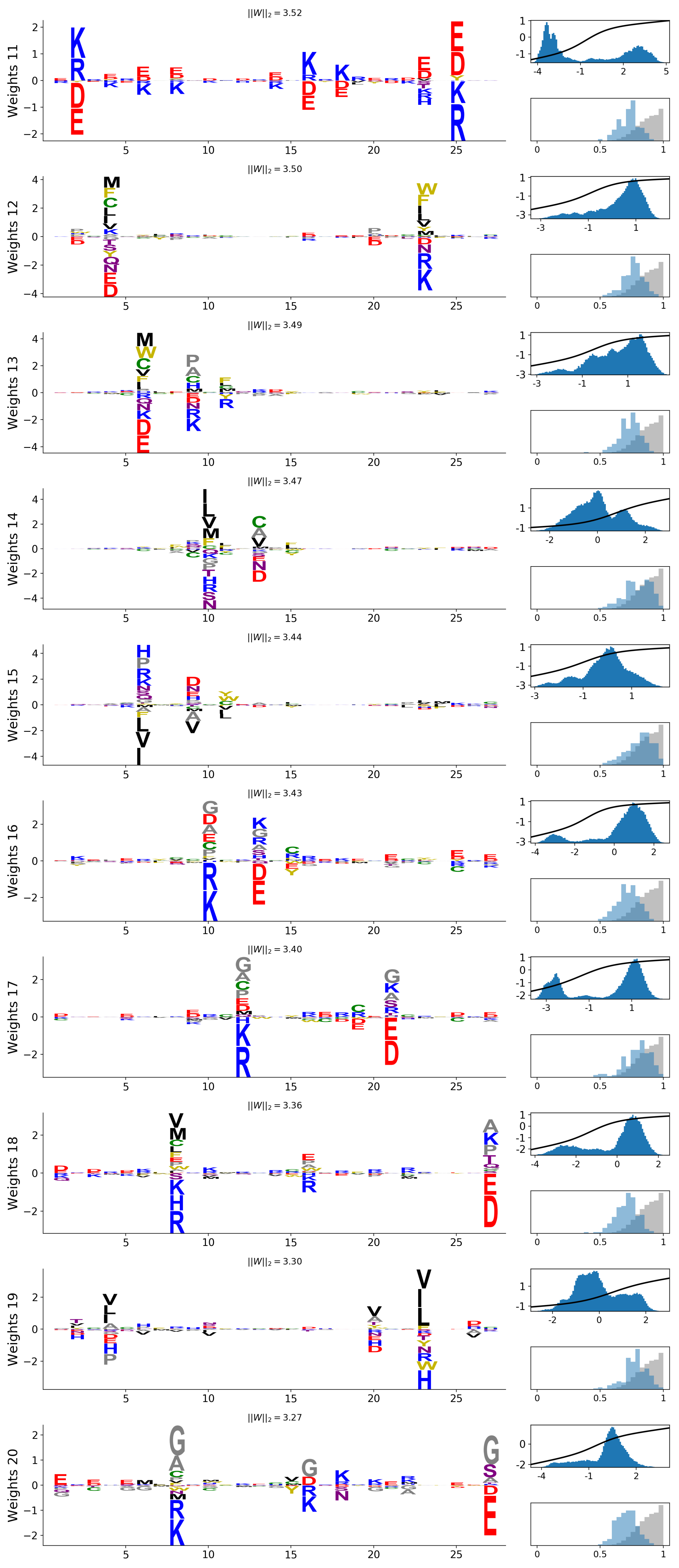

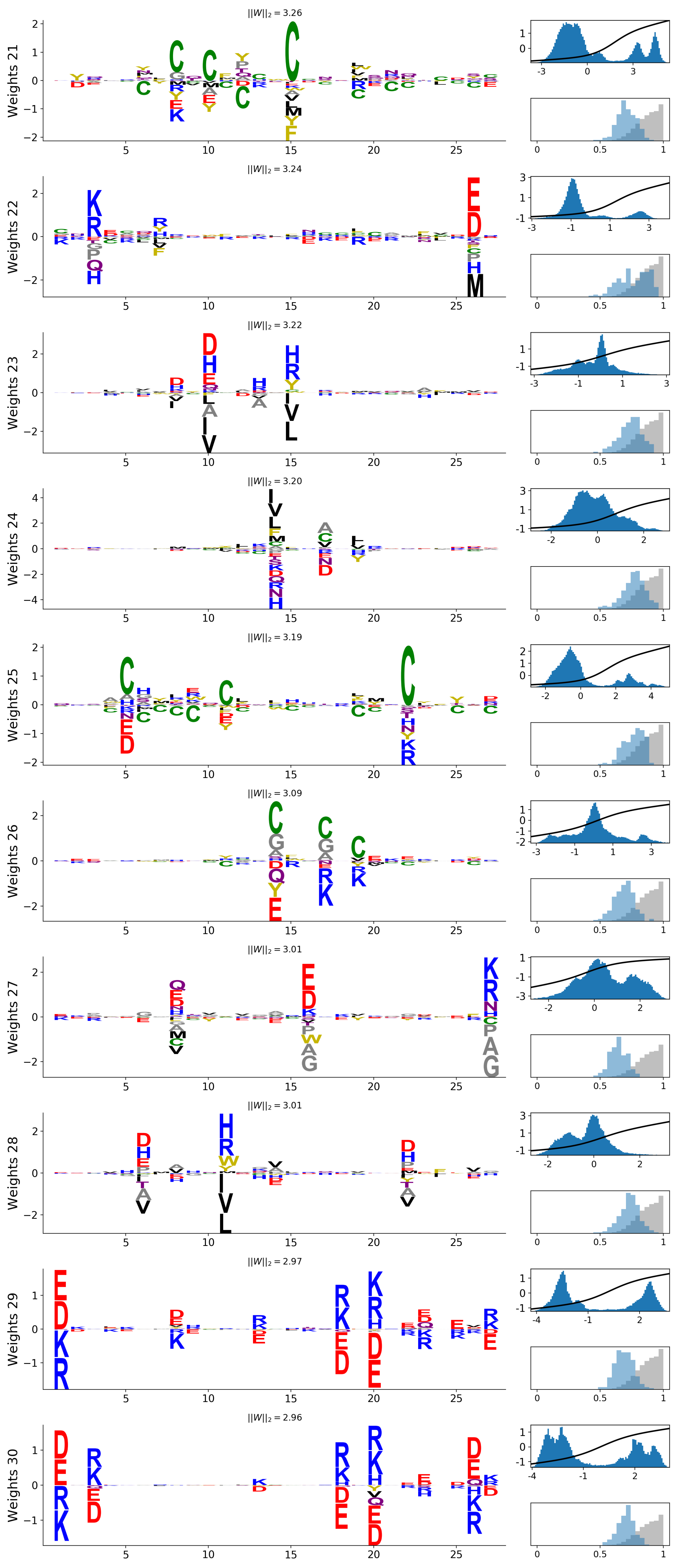

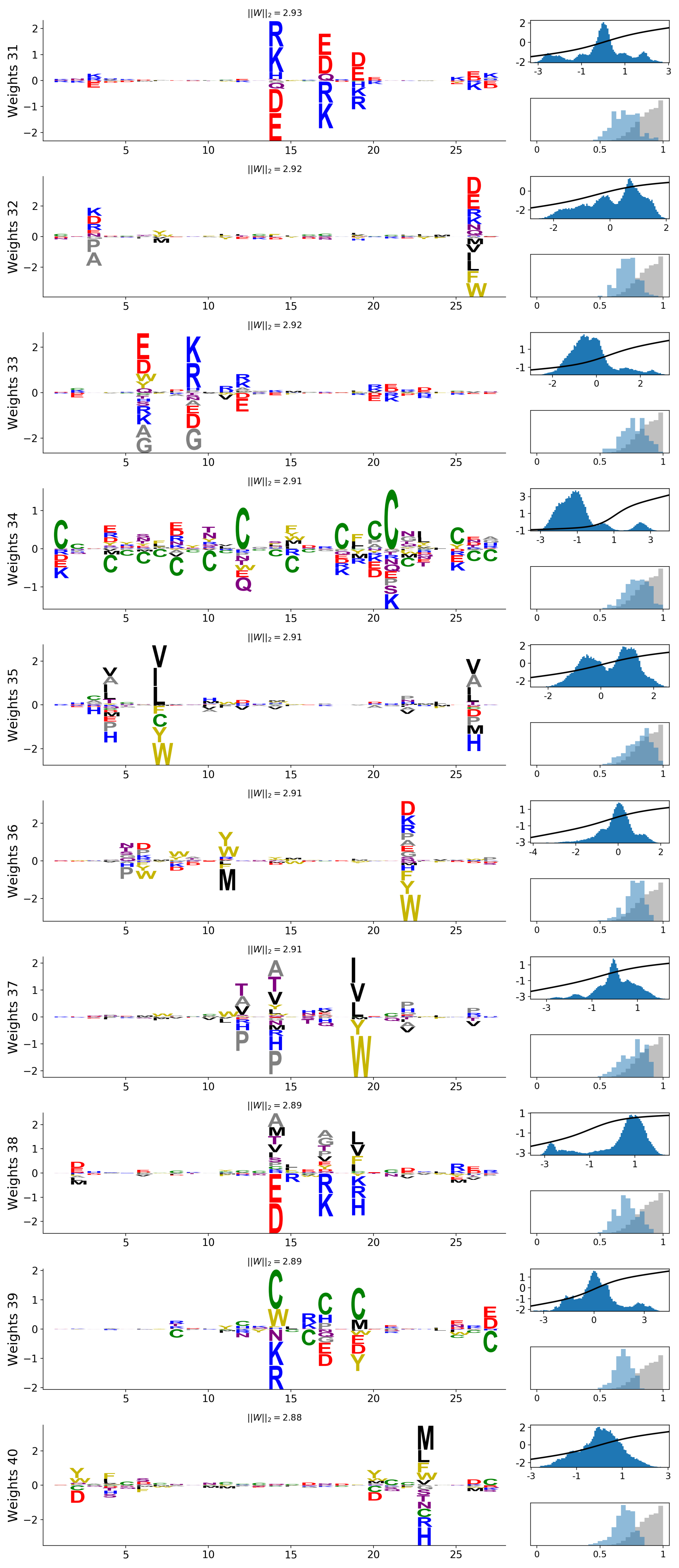

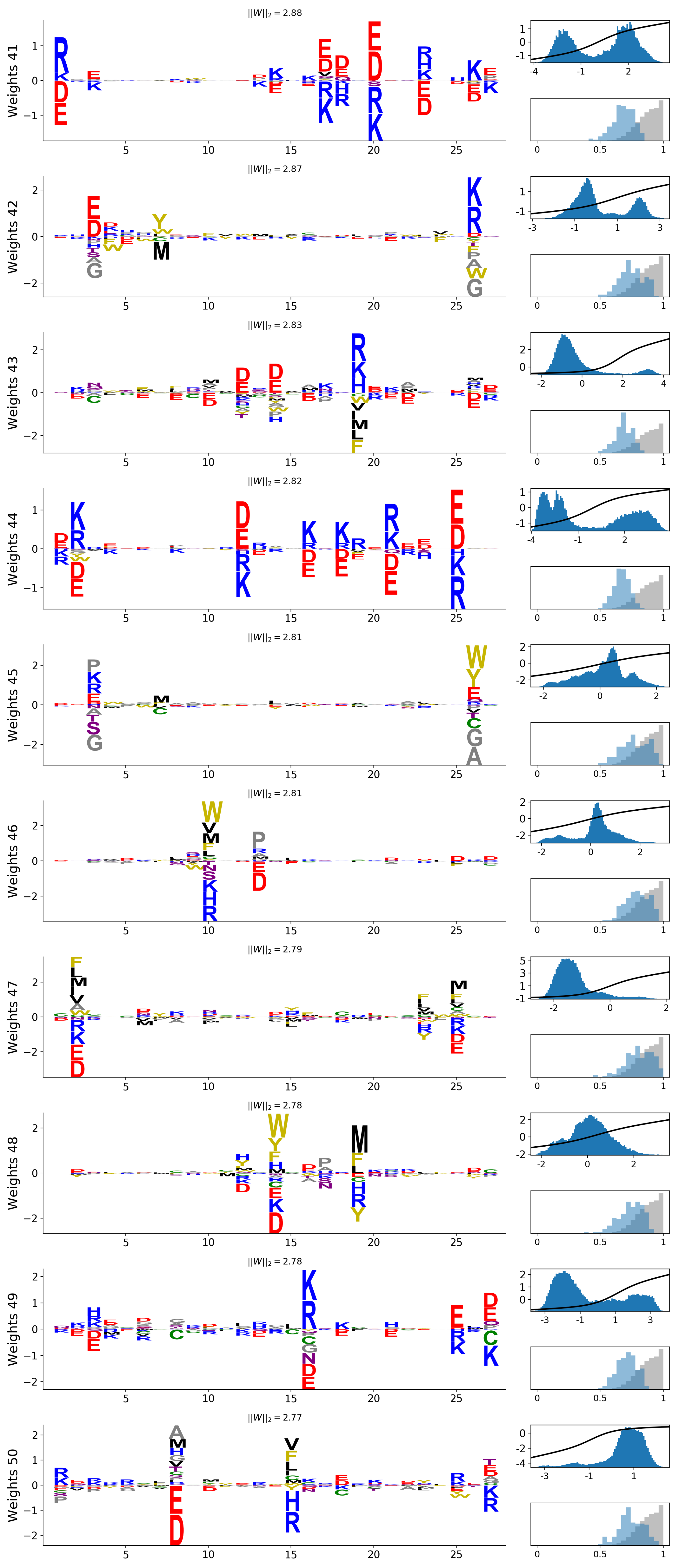

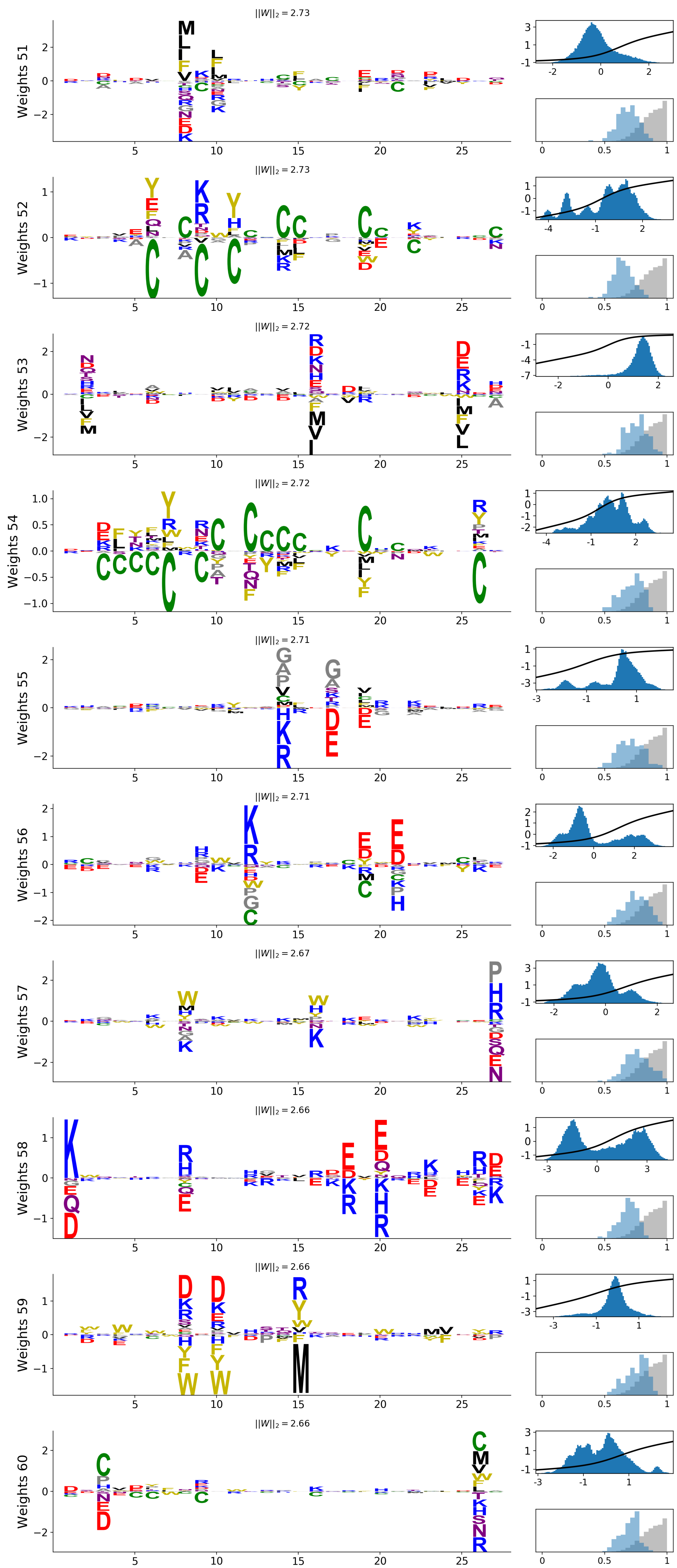

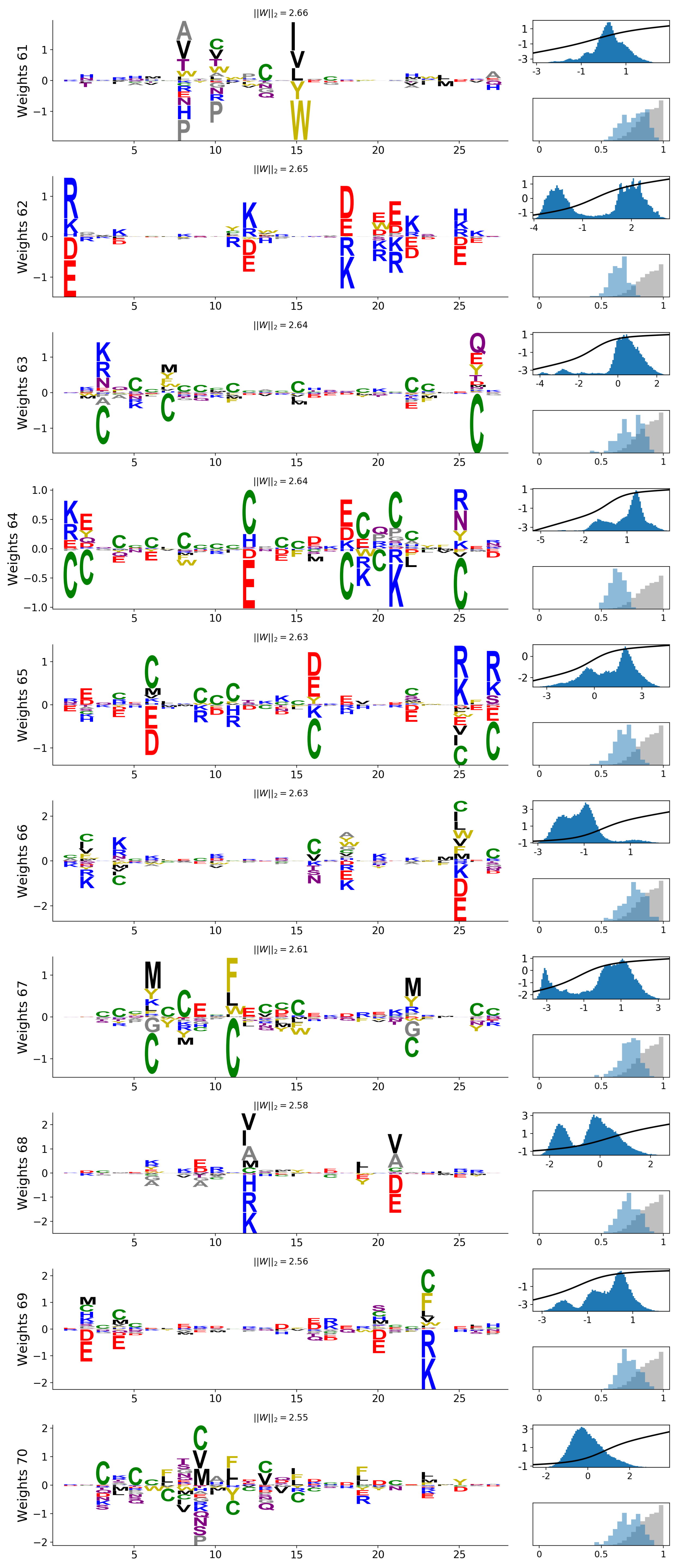

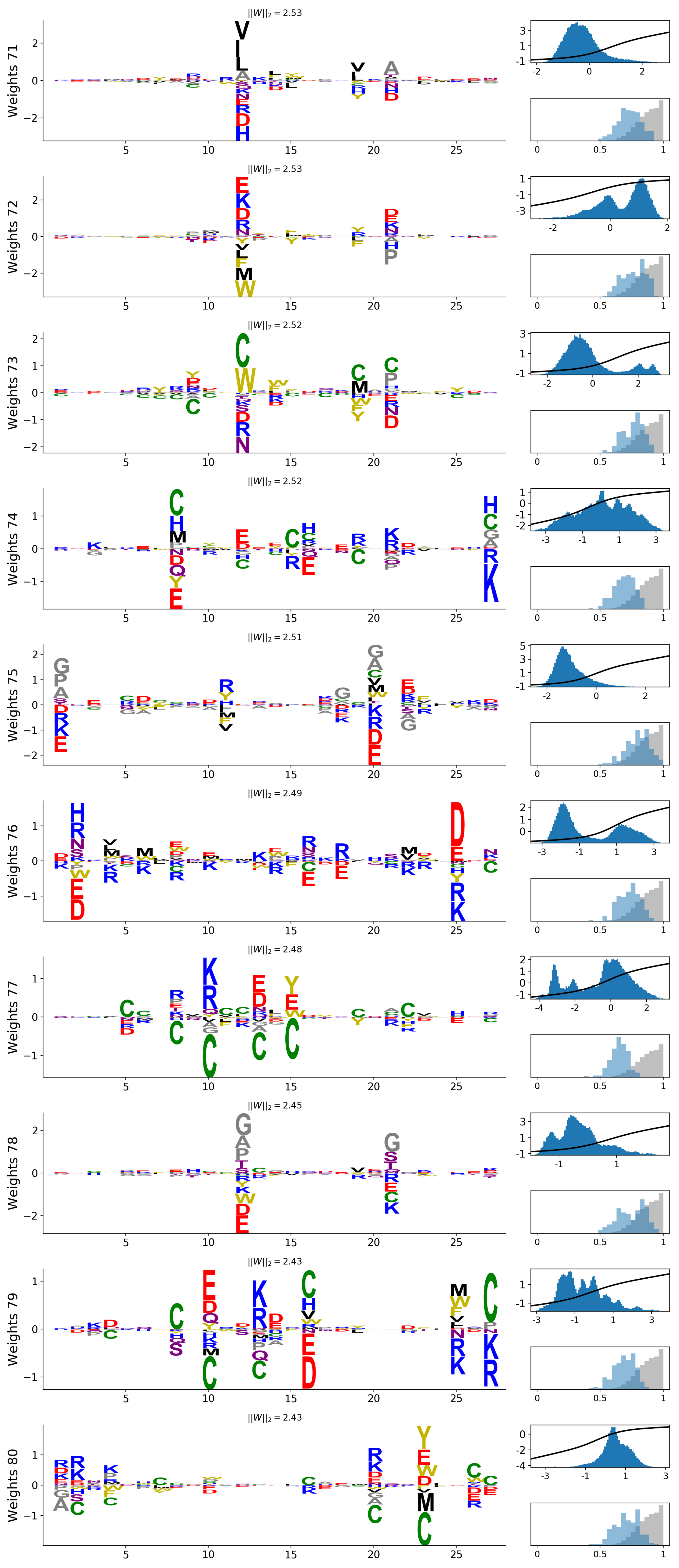

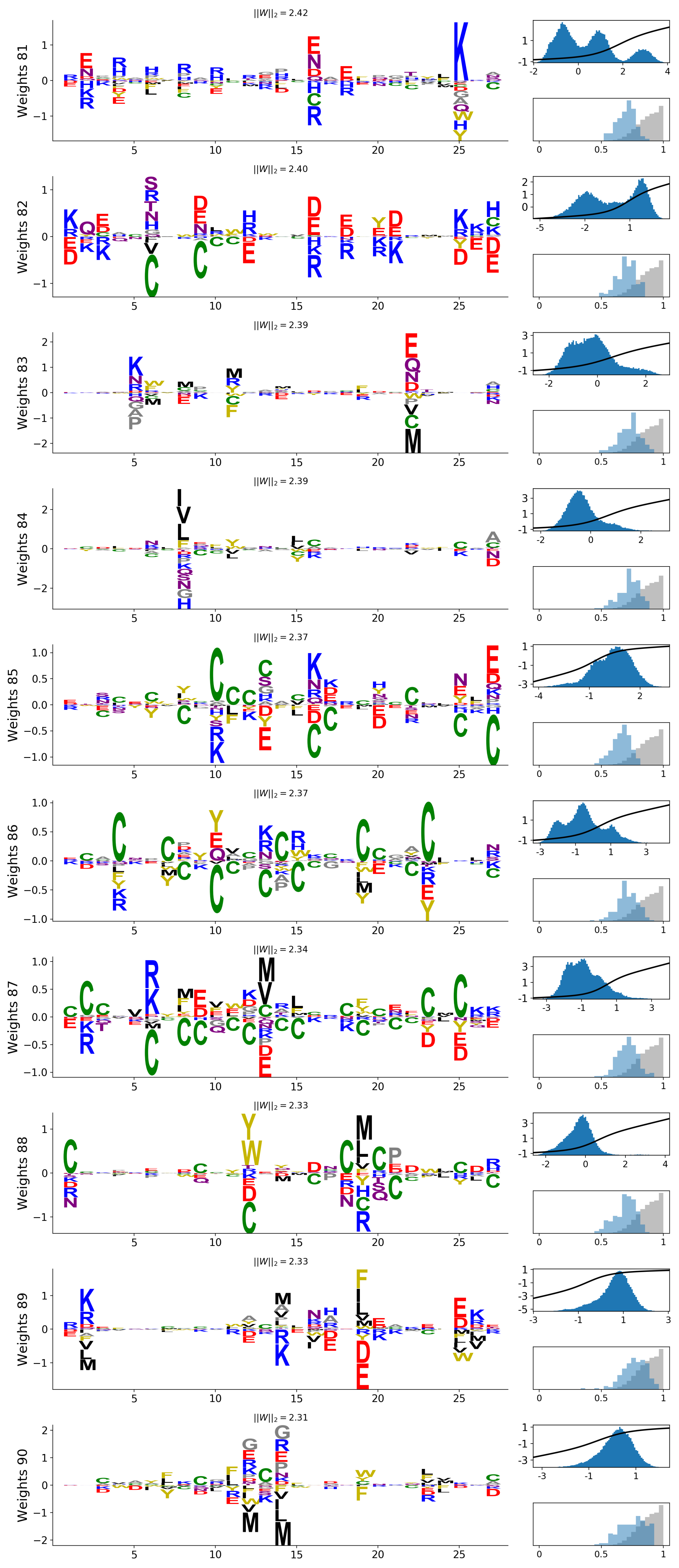

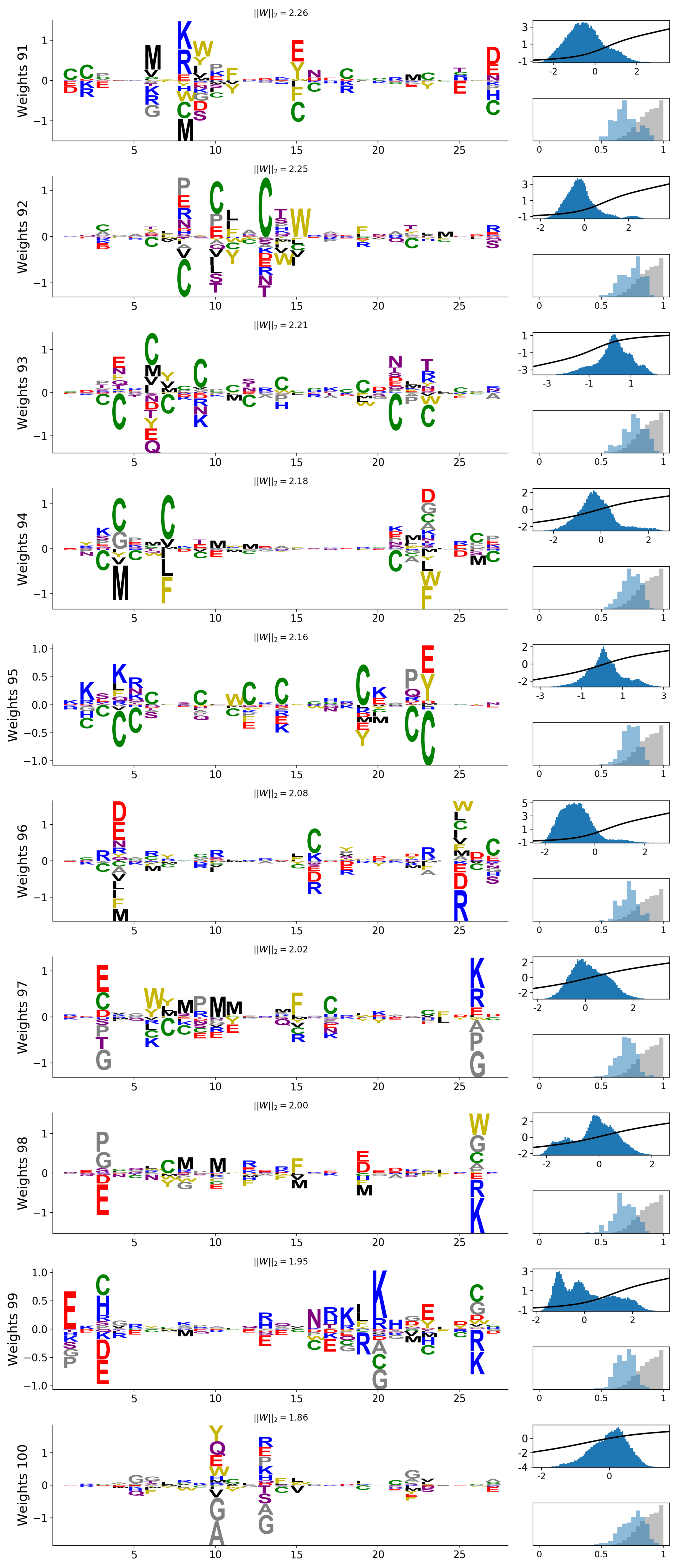

Supplement: Supplementary file 3. [file elife-39397-supp3.pdf]

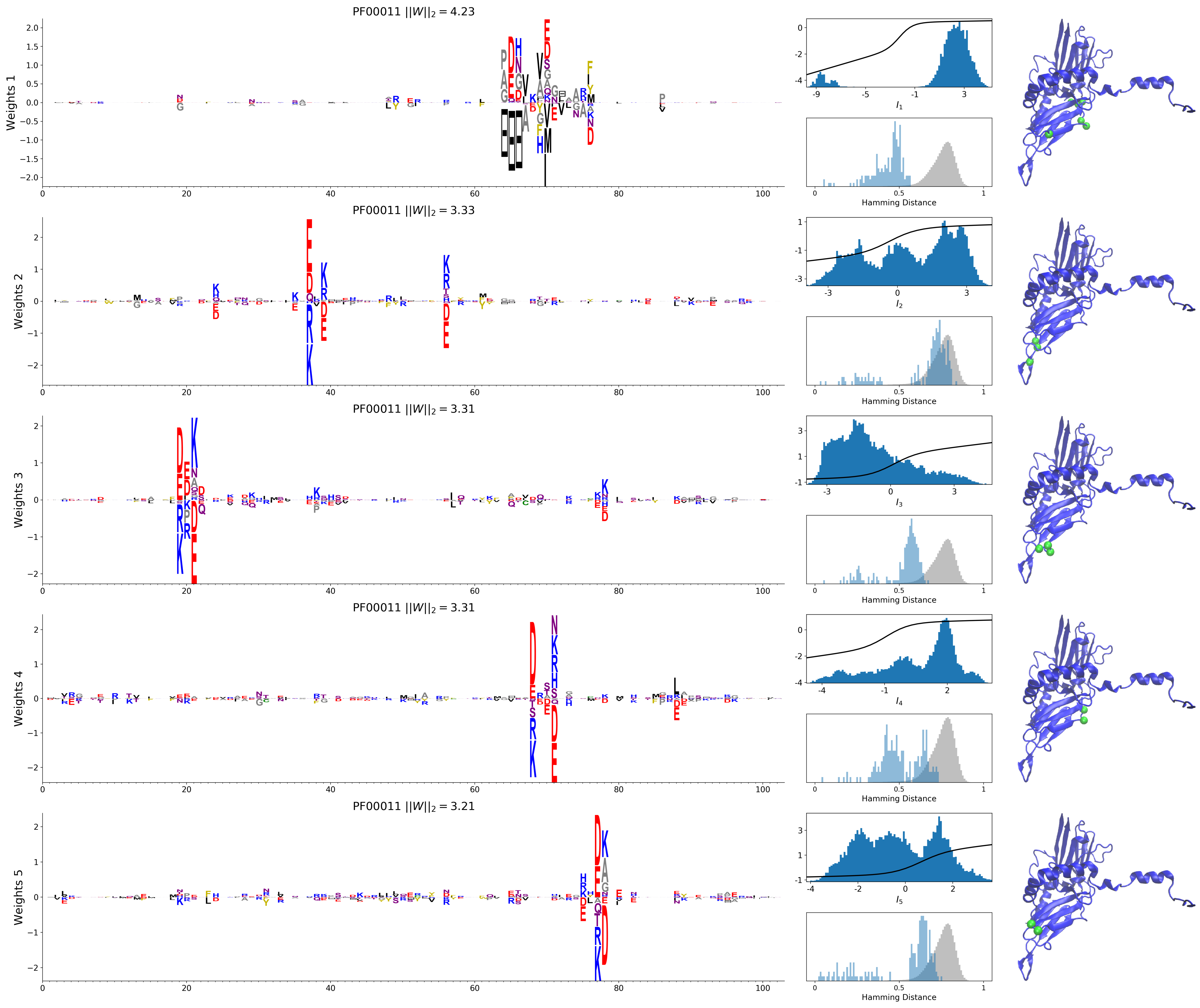

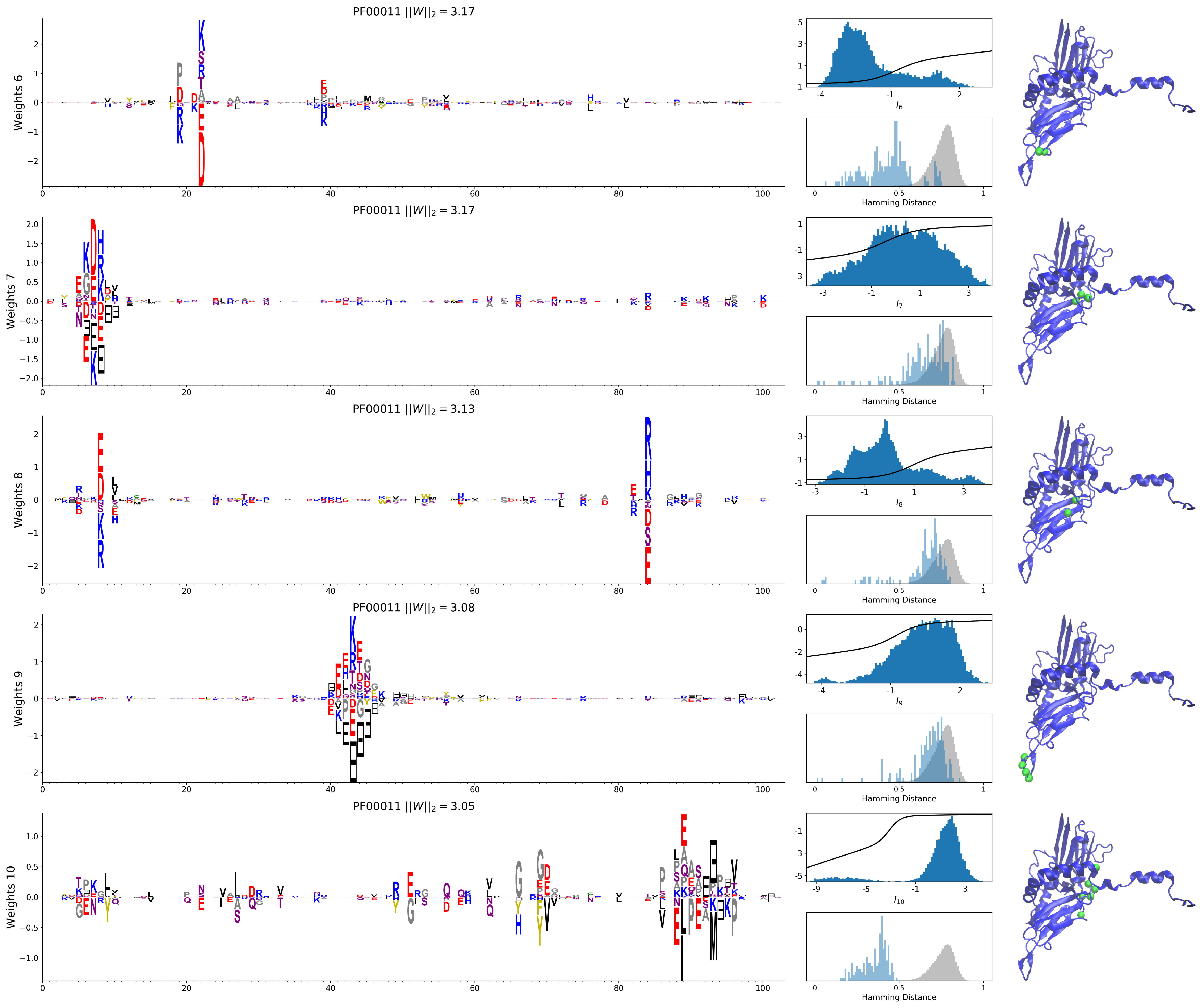

Supplement: Supplementary file 5. [file elife-39397-supp5.zip › Top_features_all/PF00011_top_features.pdf]

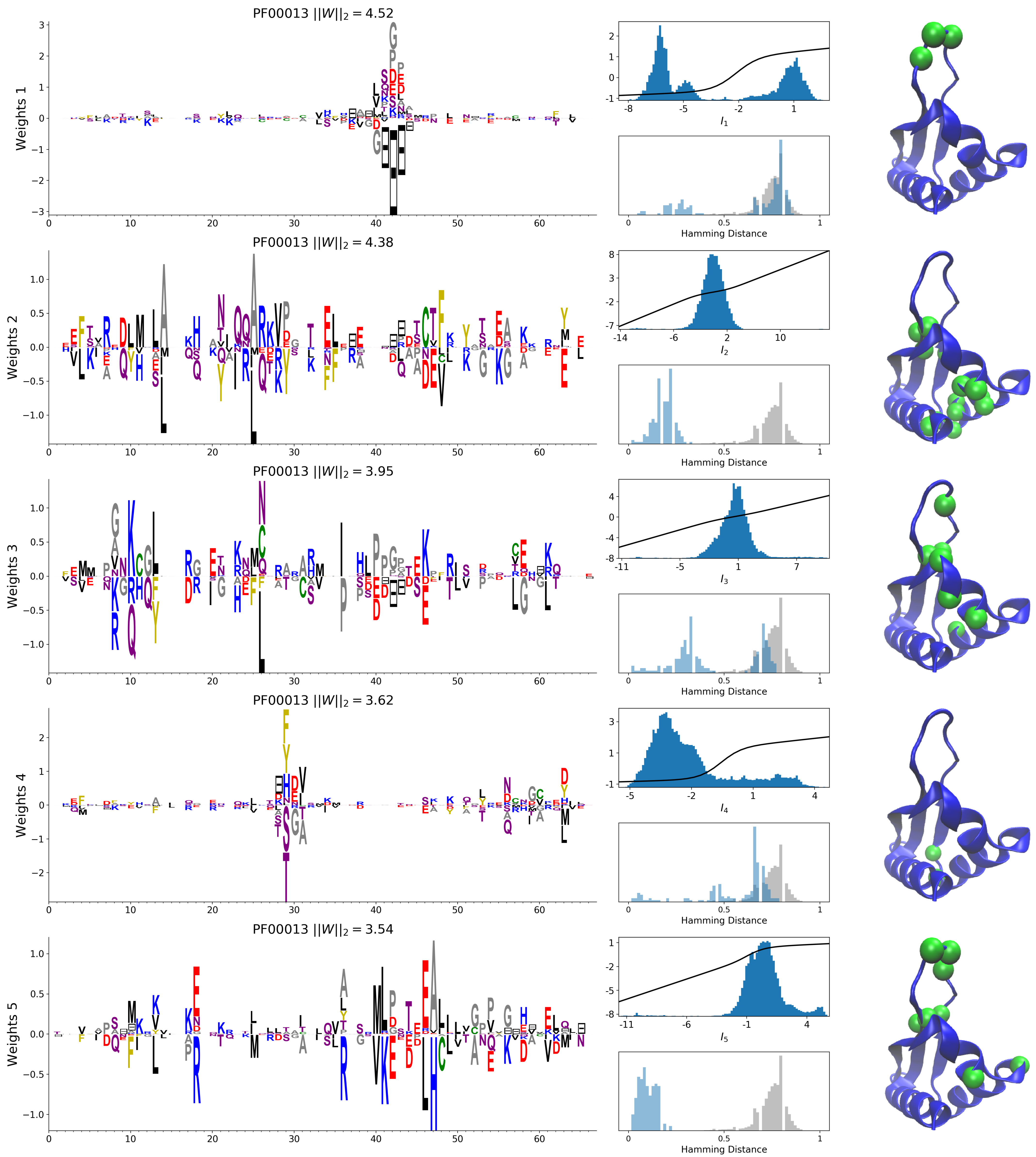

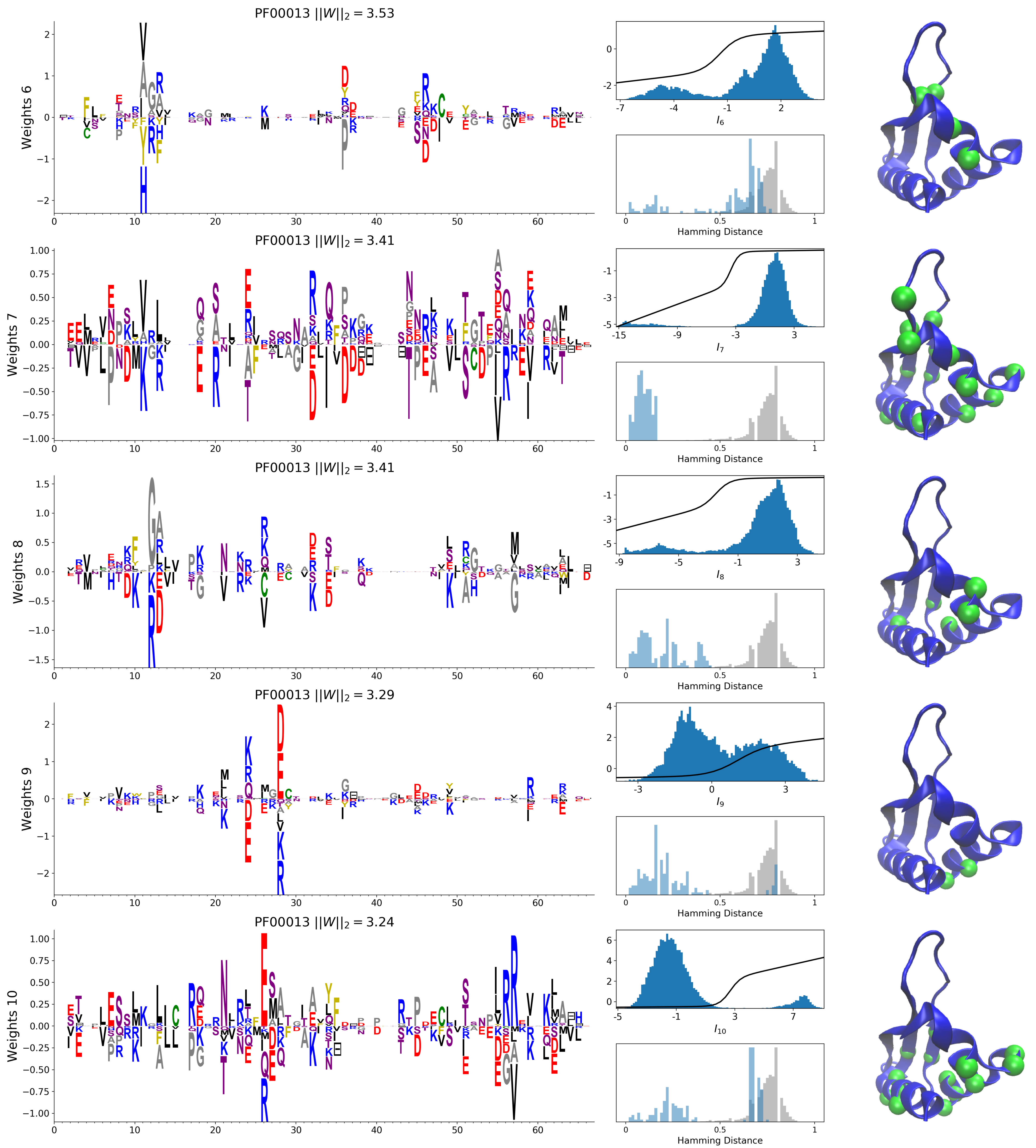

Supplement: Supplementary file 5. [file elife-39397-supp5.zip › Top_features_all/PF00013_top_features.pdf]

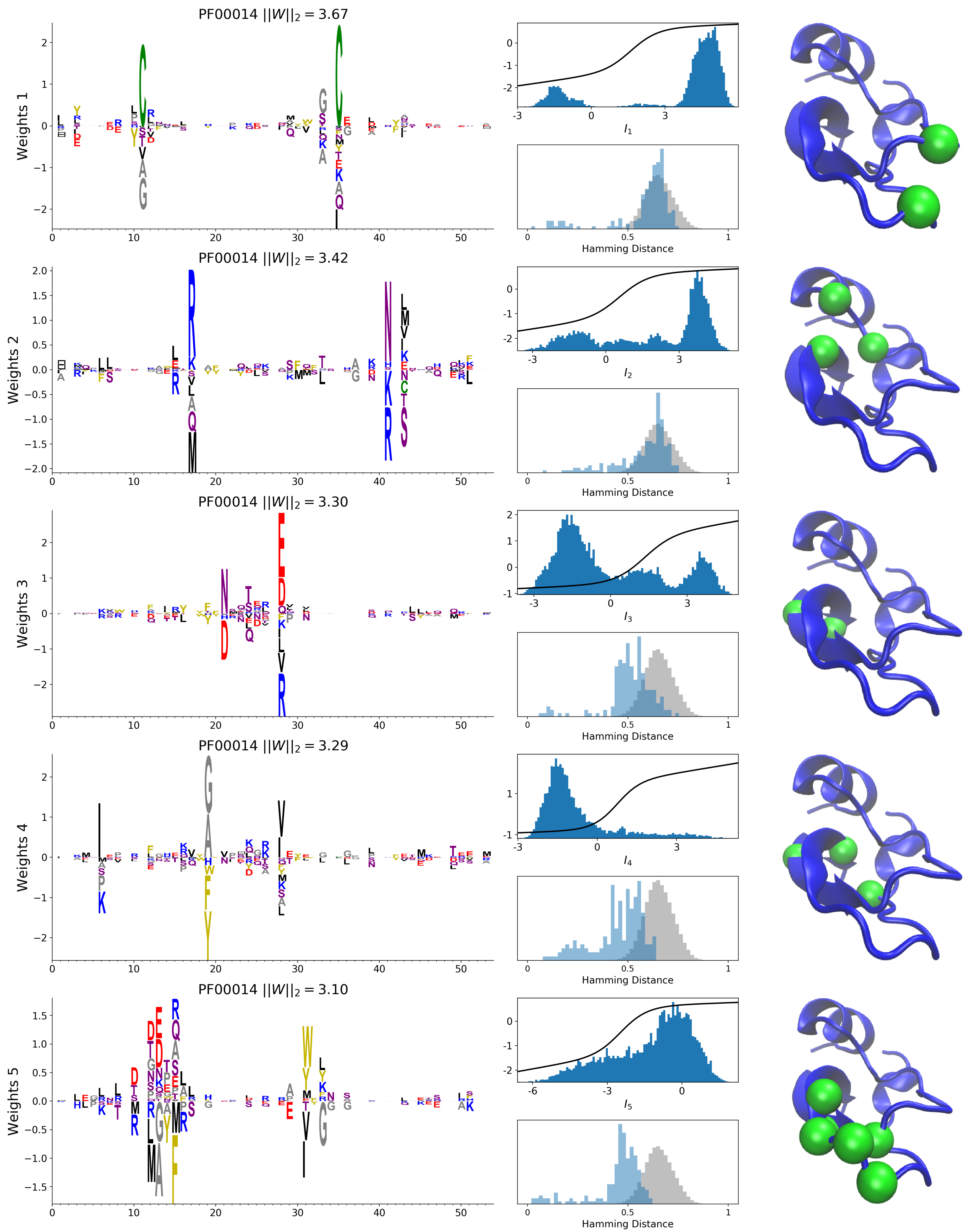

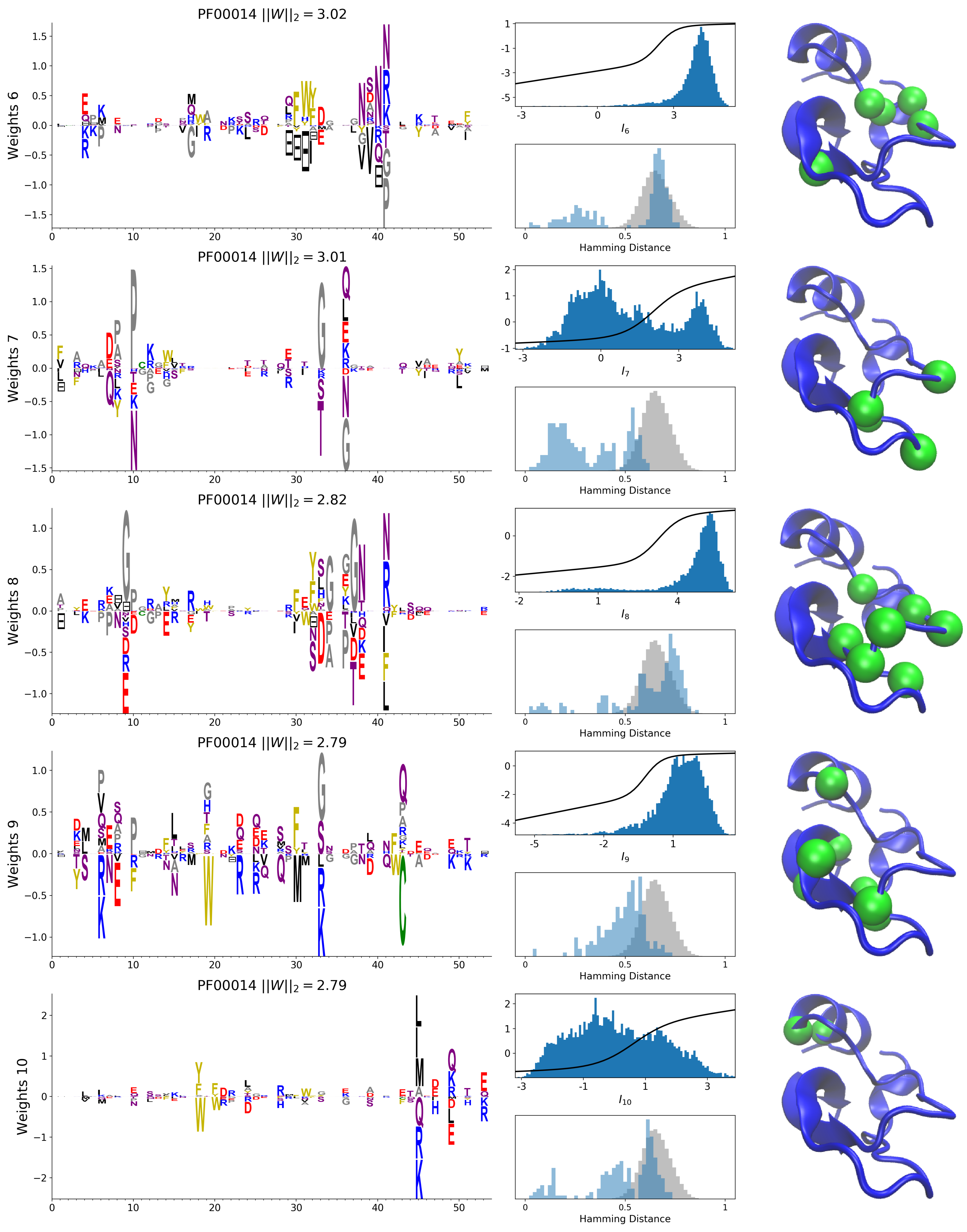

Supplement: Supplementary file 5. [file elife-39397-supp5.zip › Top_features_all/PF00014_top_features.pdf]

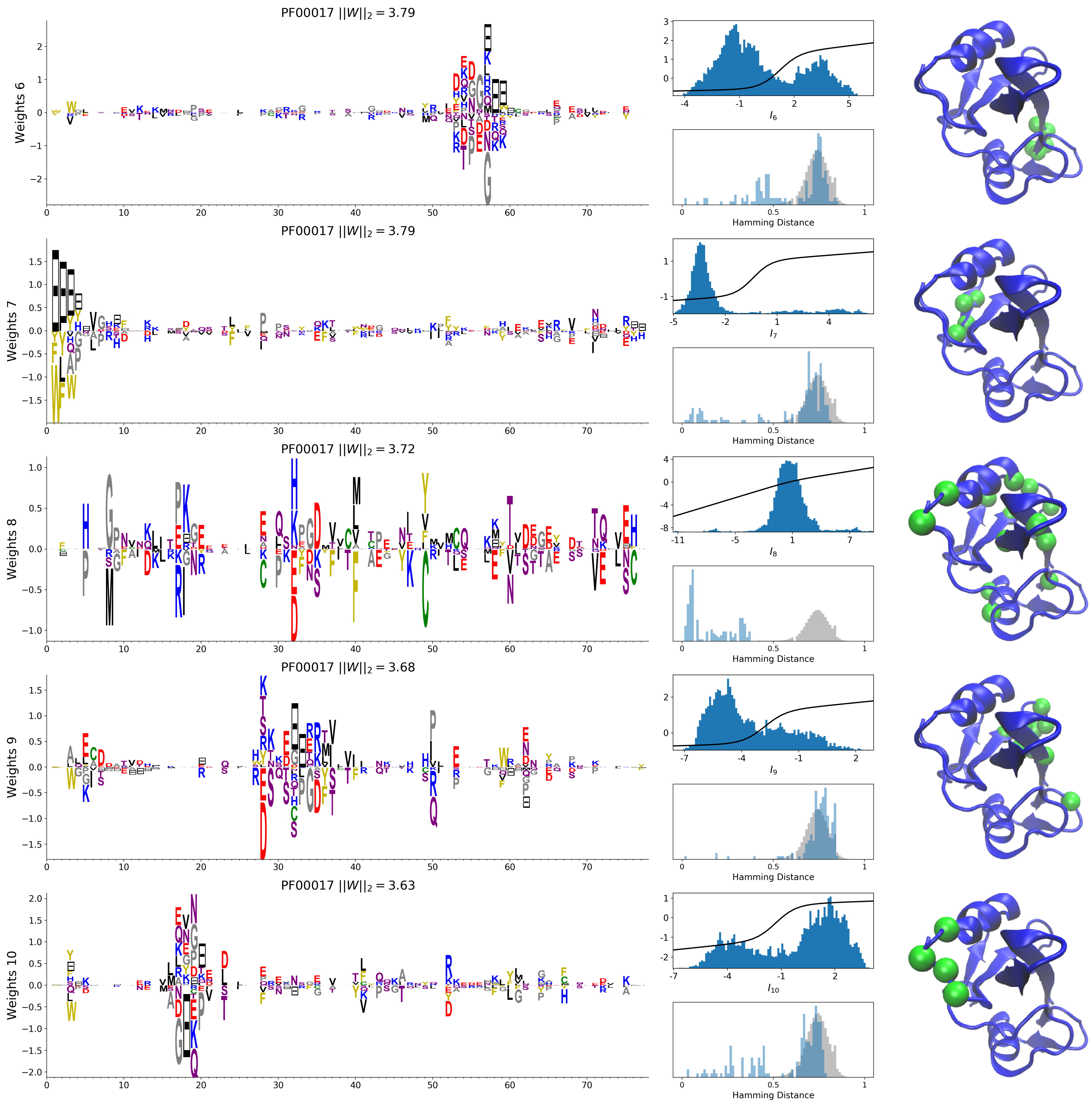

Supplement: Supplementary file 5. [file elife-39397-supp5.zip › Top_features_all/PF00017_top_features.pdf]

PF00018  $\|W\|_2 = 4.33$

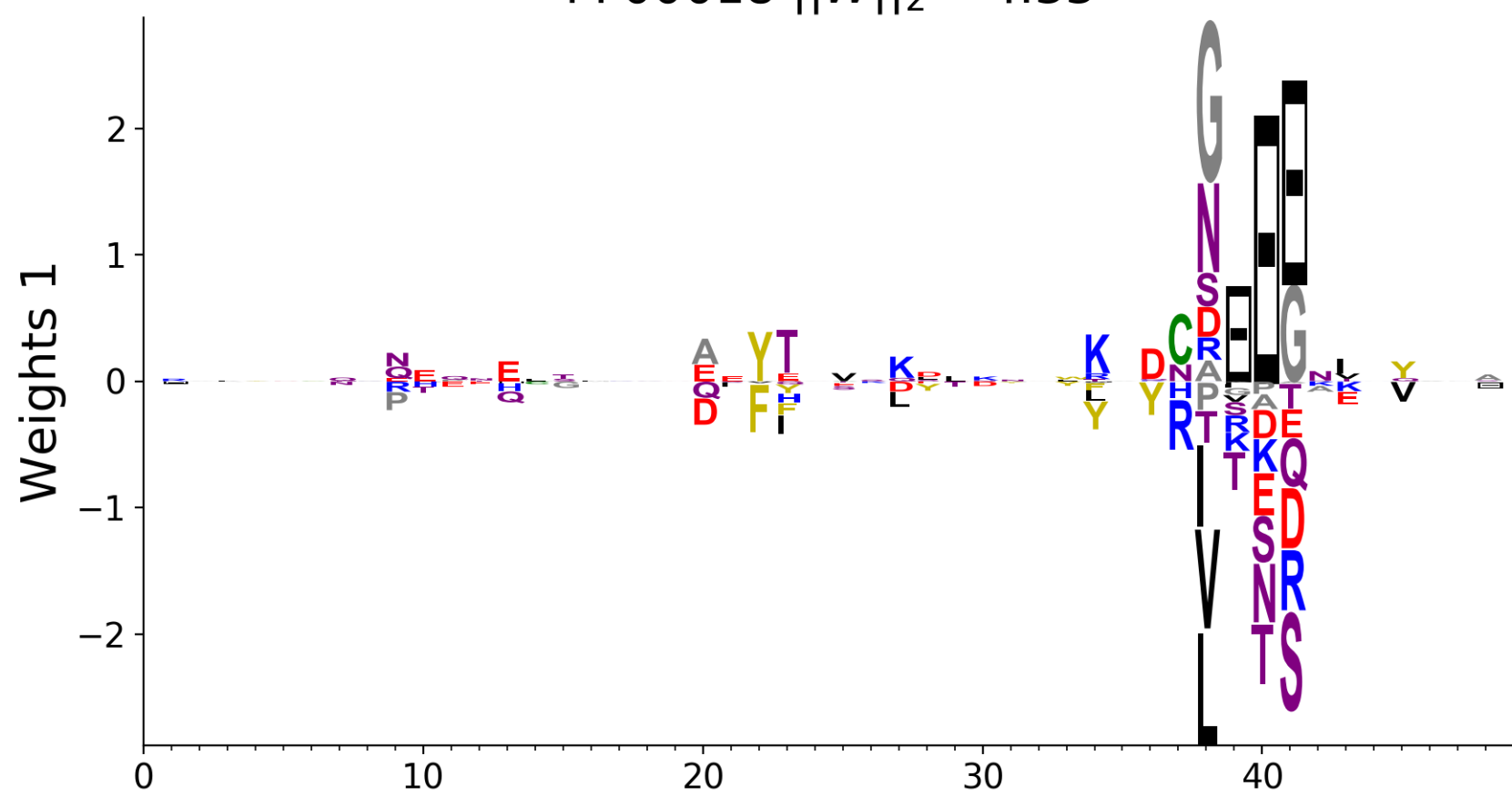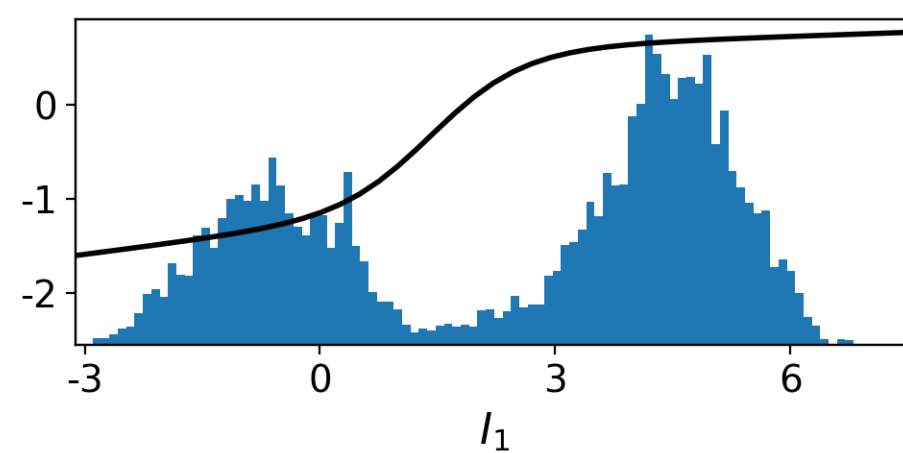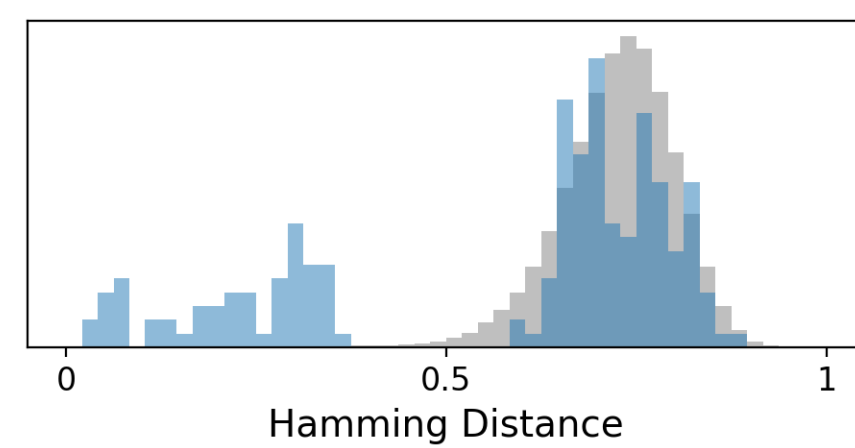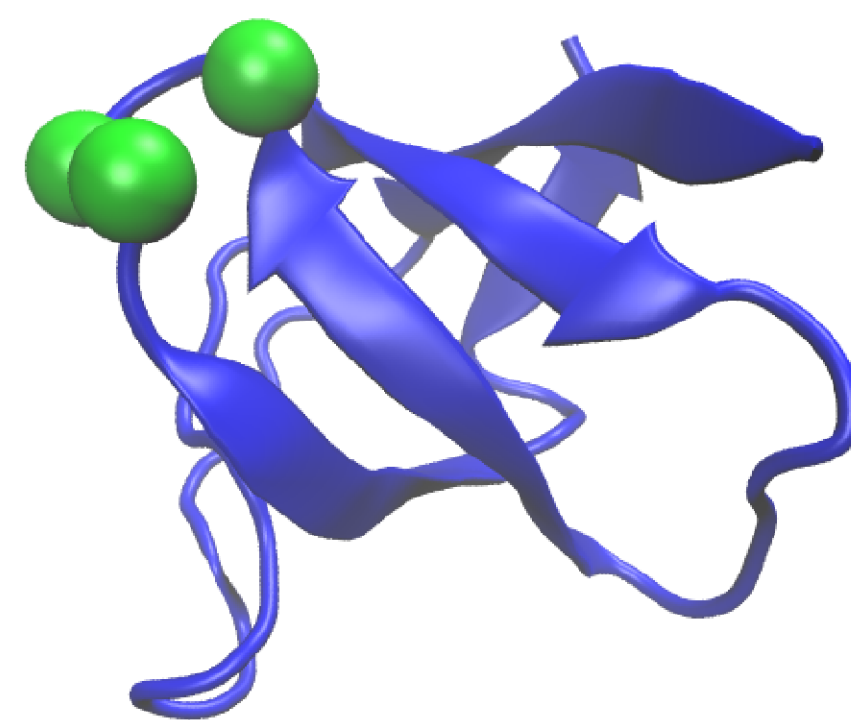

PF00018  $\|W\|_2 = 4.29$

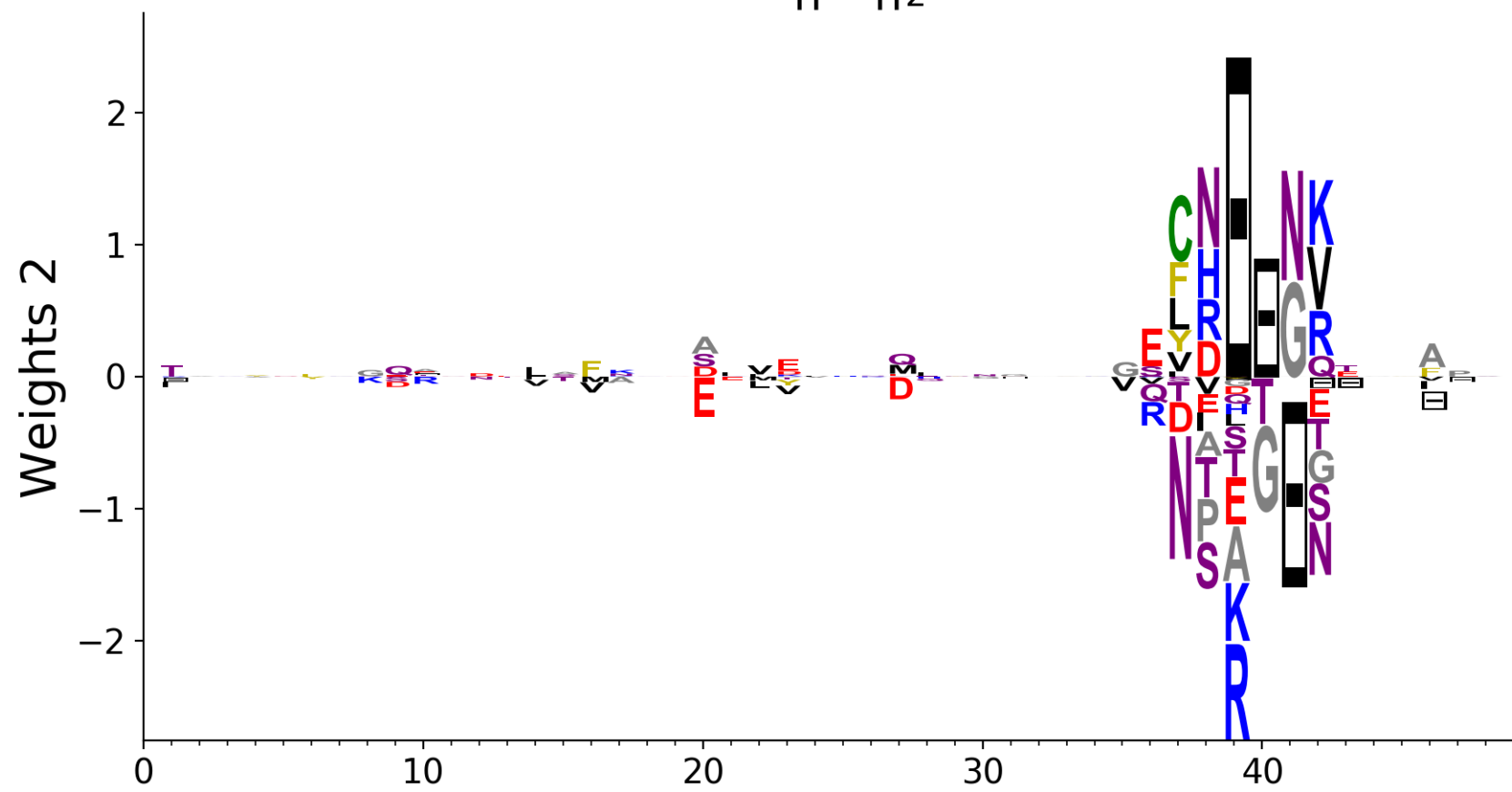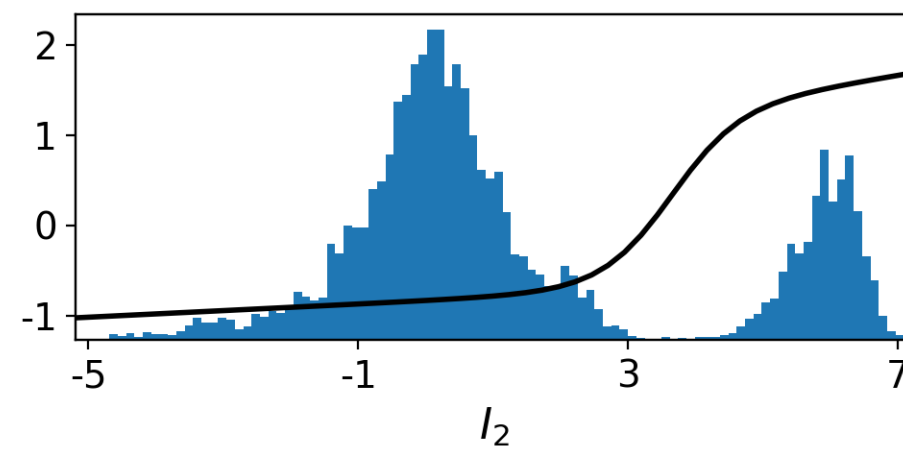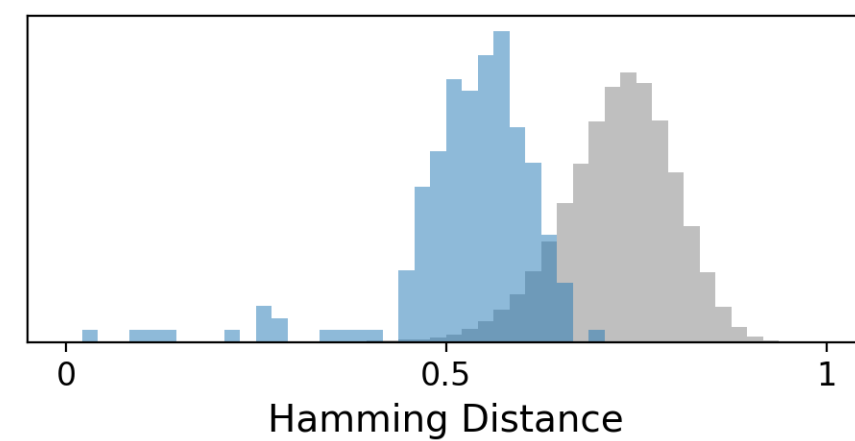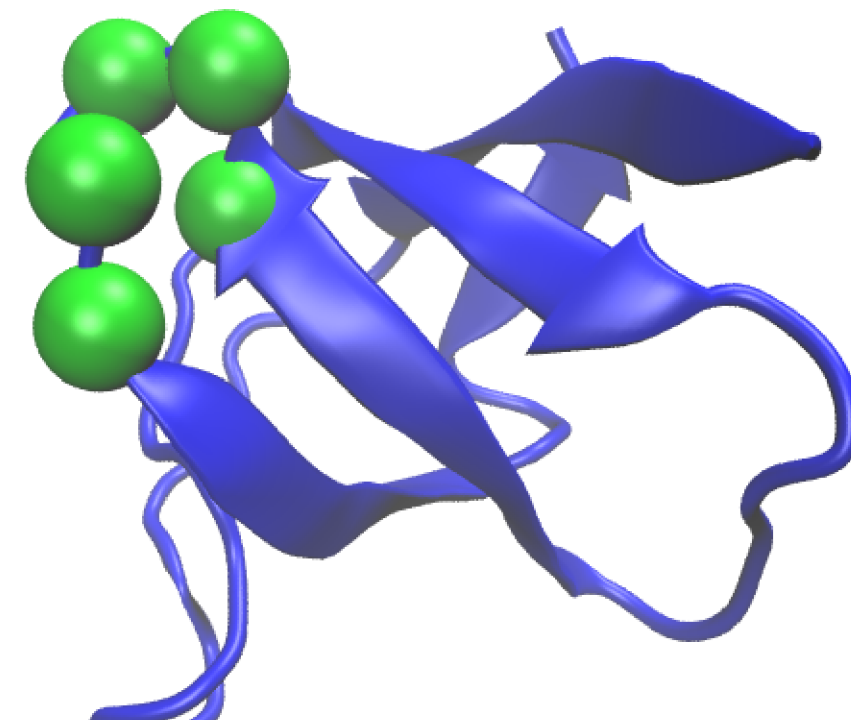

PF00018  $\|W\|_2 = 4.09$

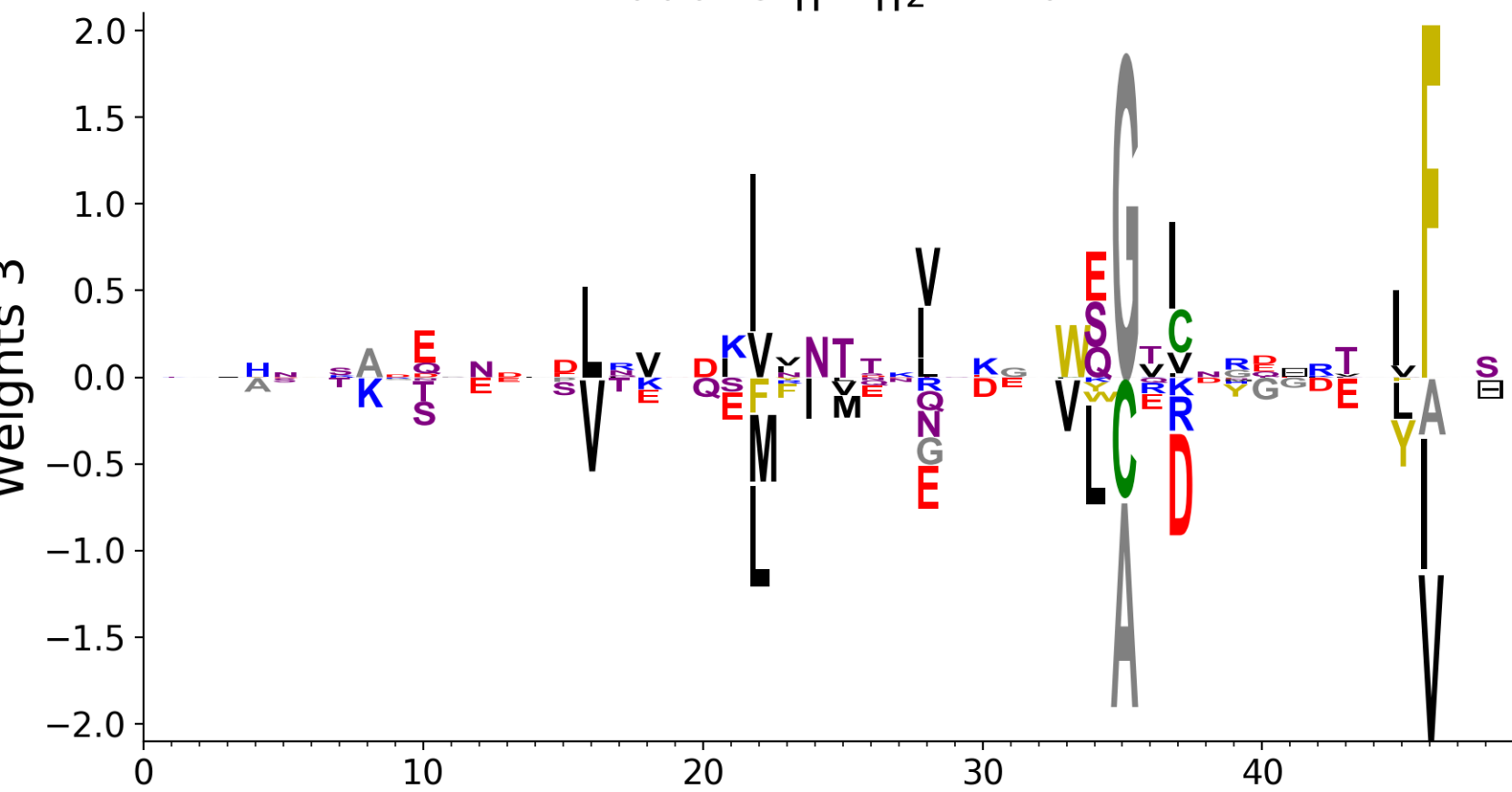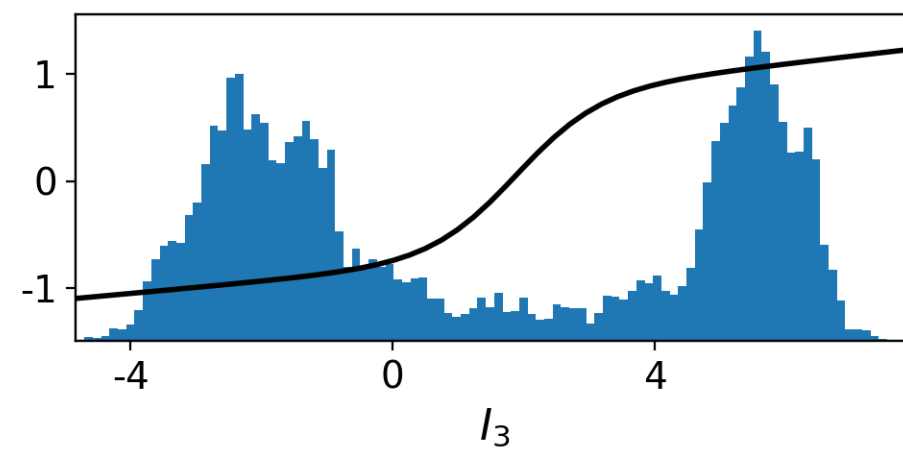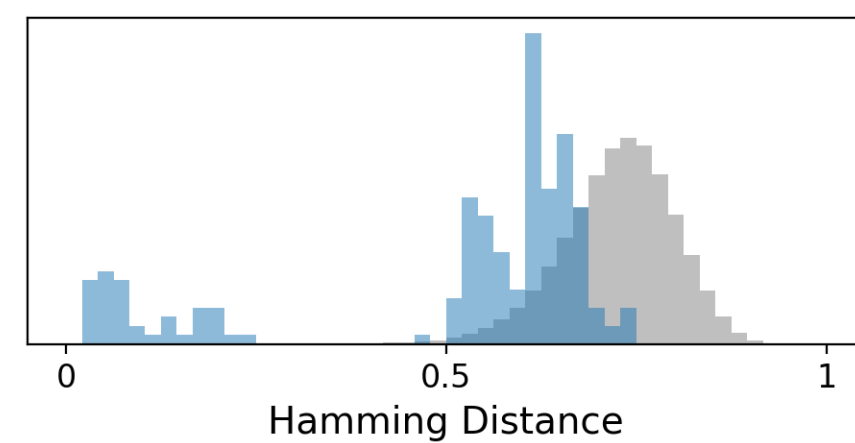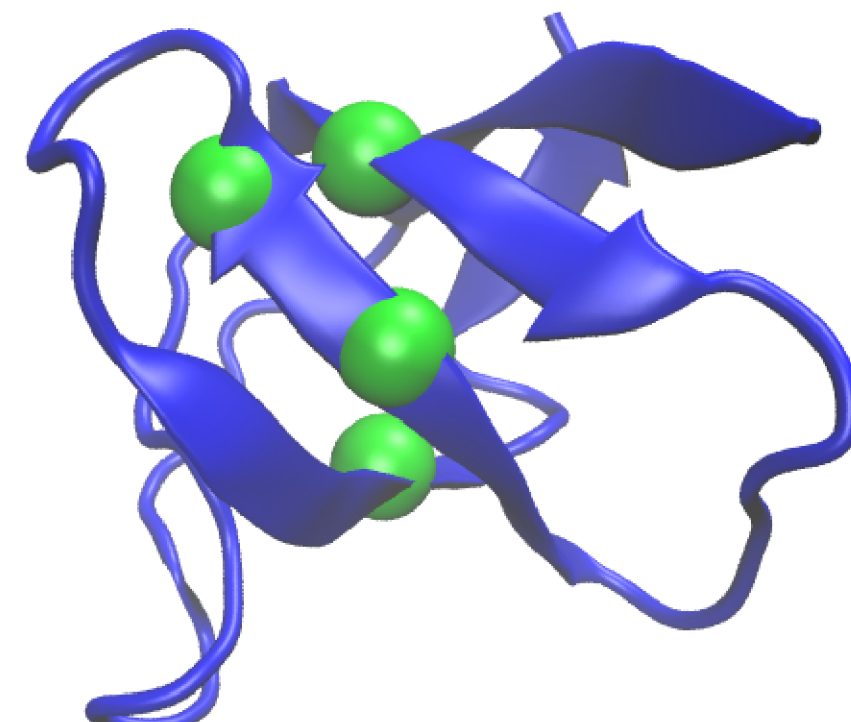

PF00018  $\|W\|_2 = 3.97$

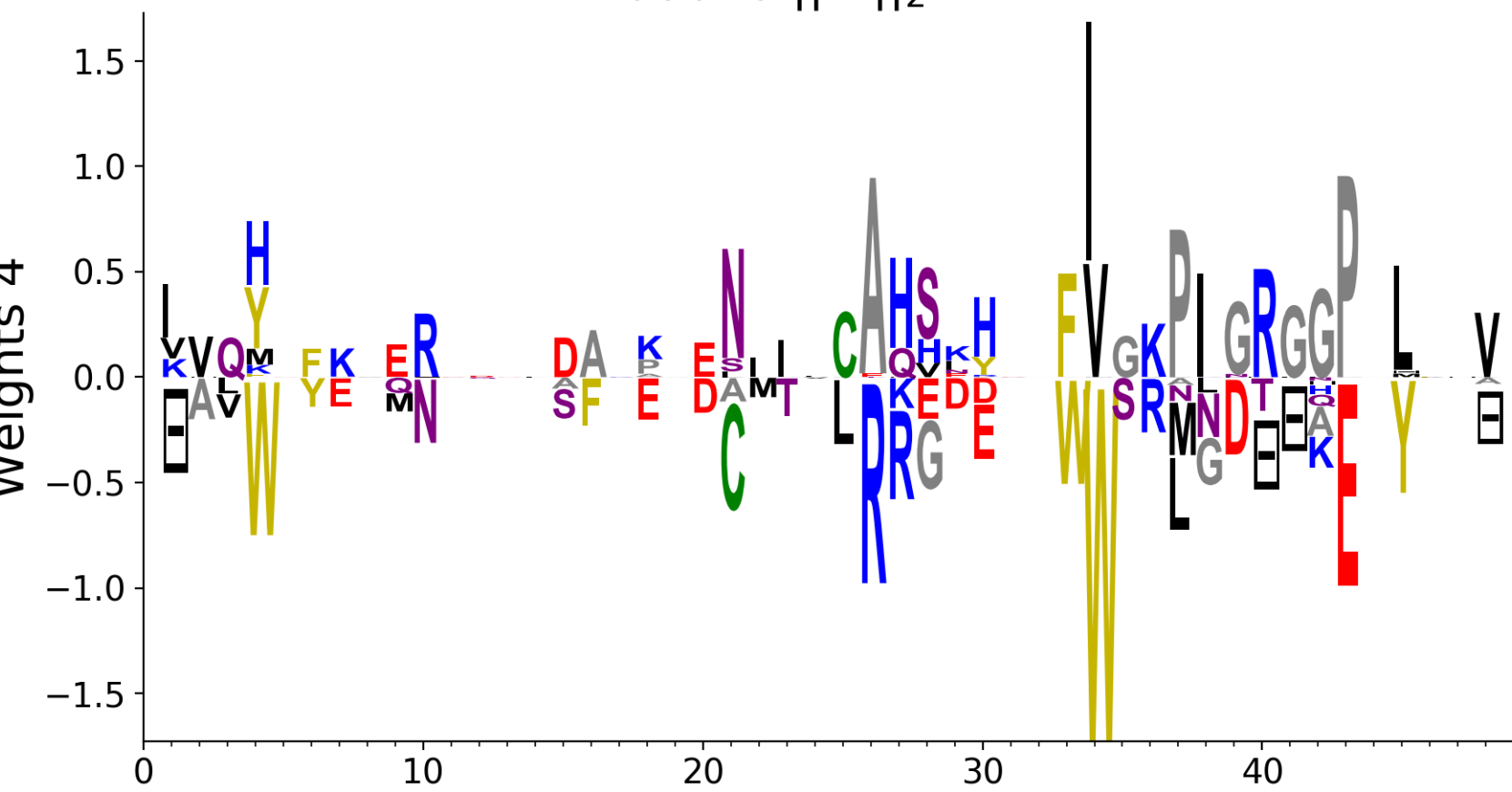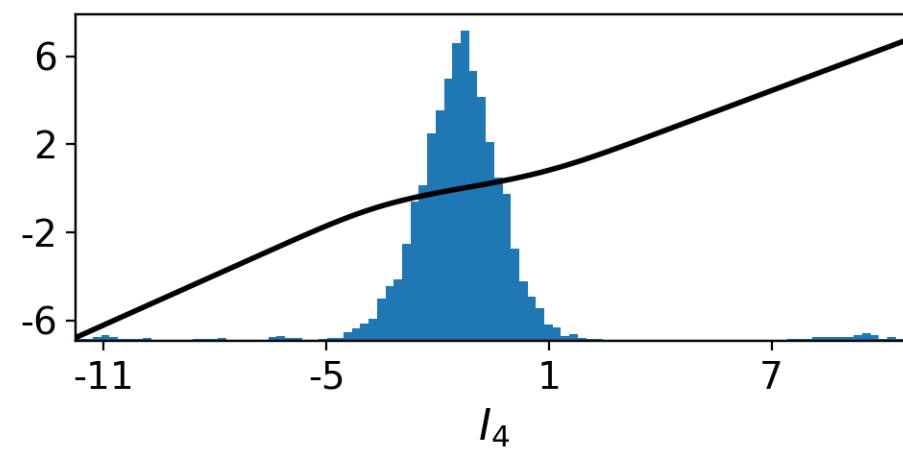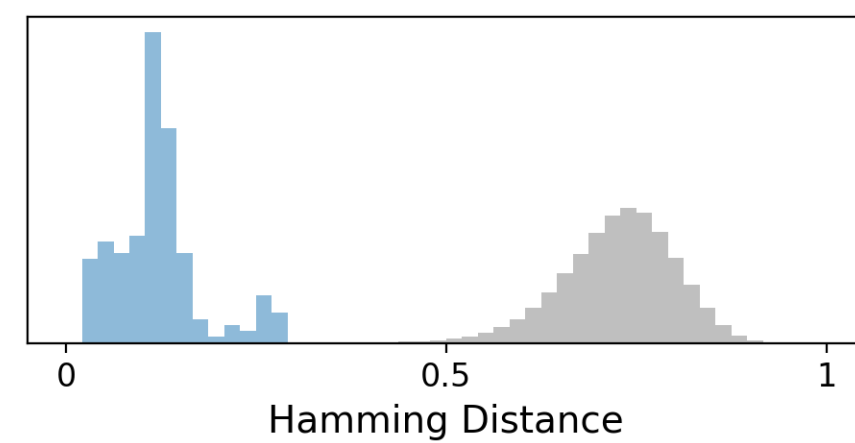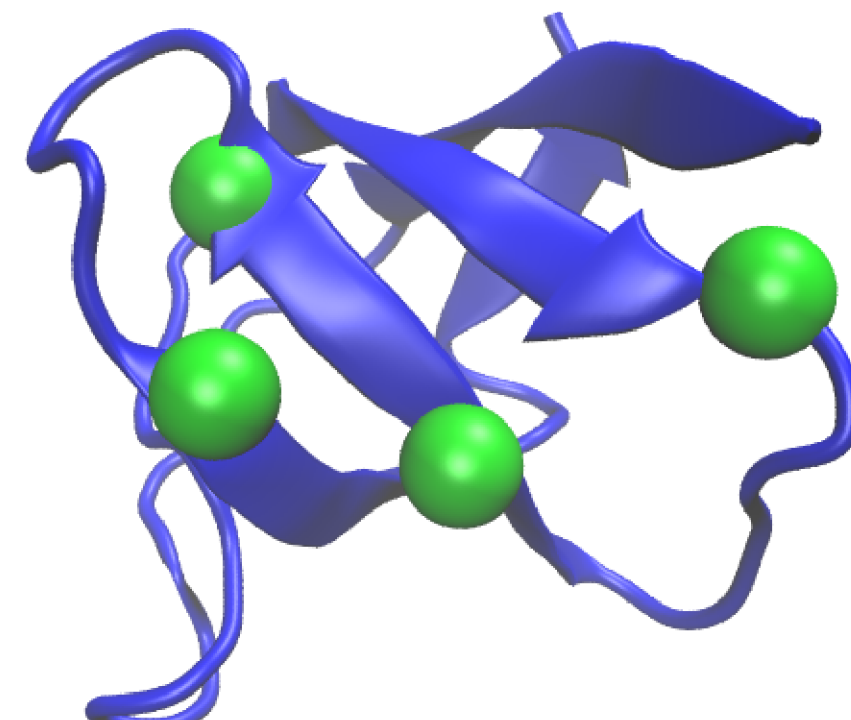

PF00018  $\|W\|_2 = 3.87$

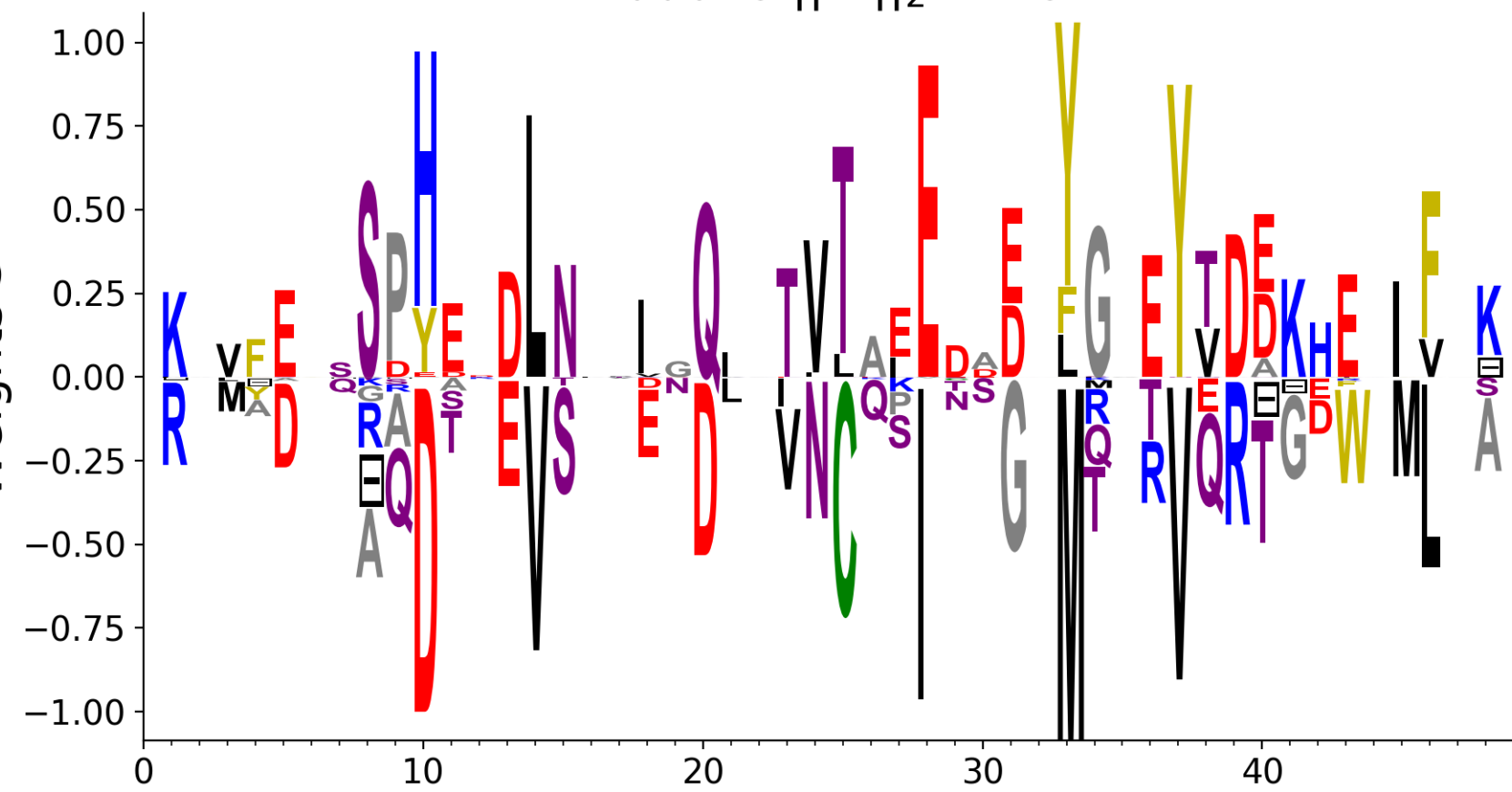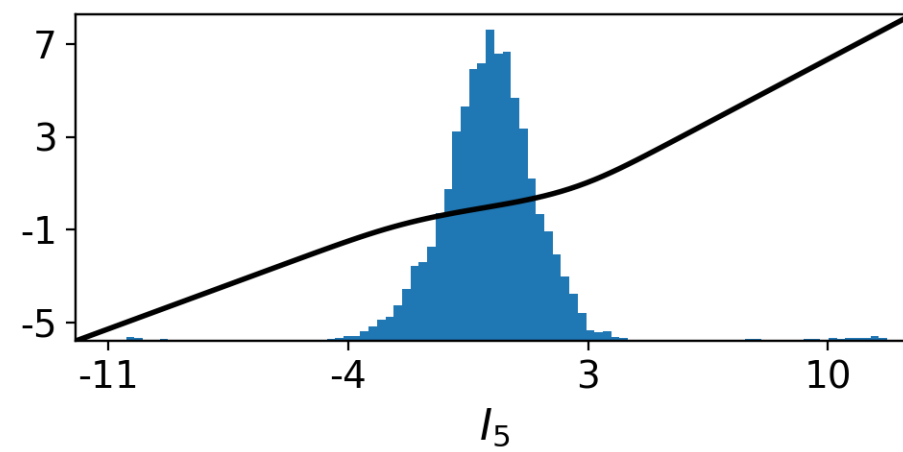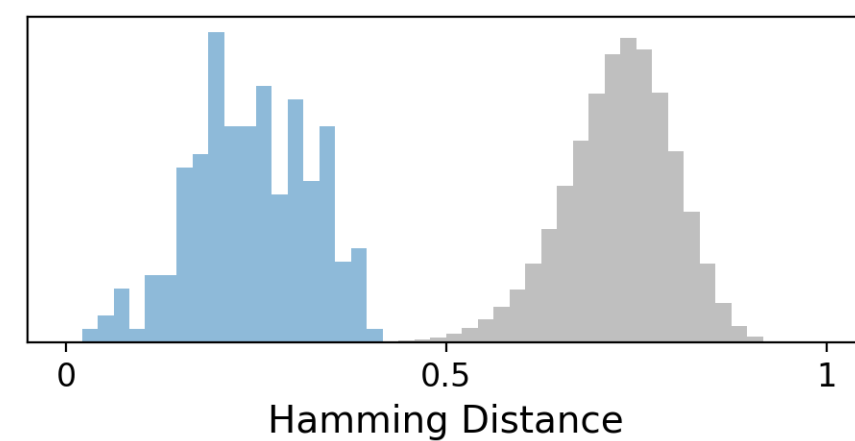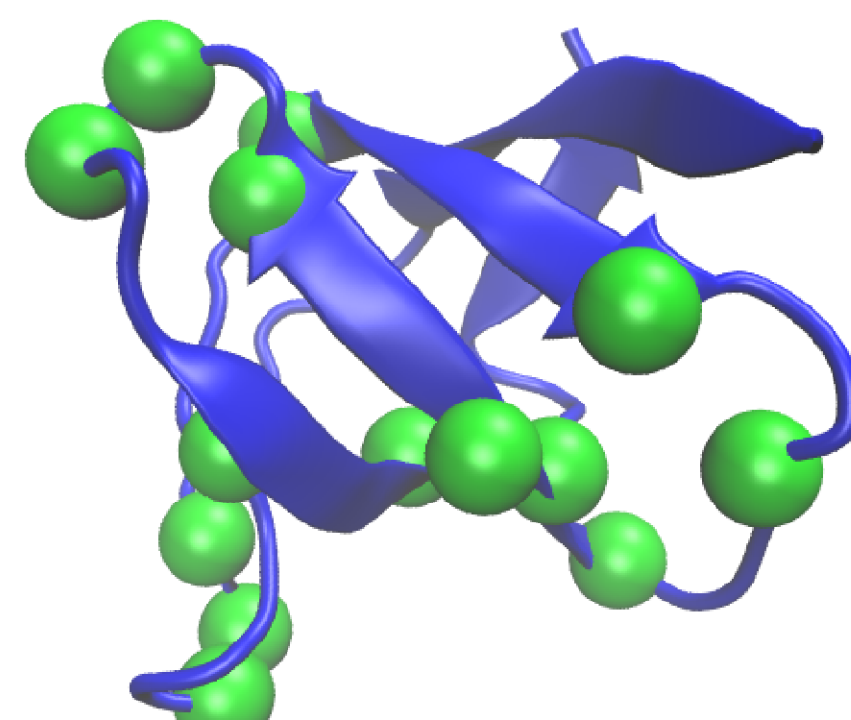

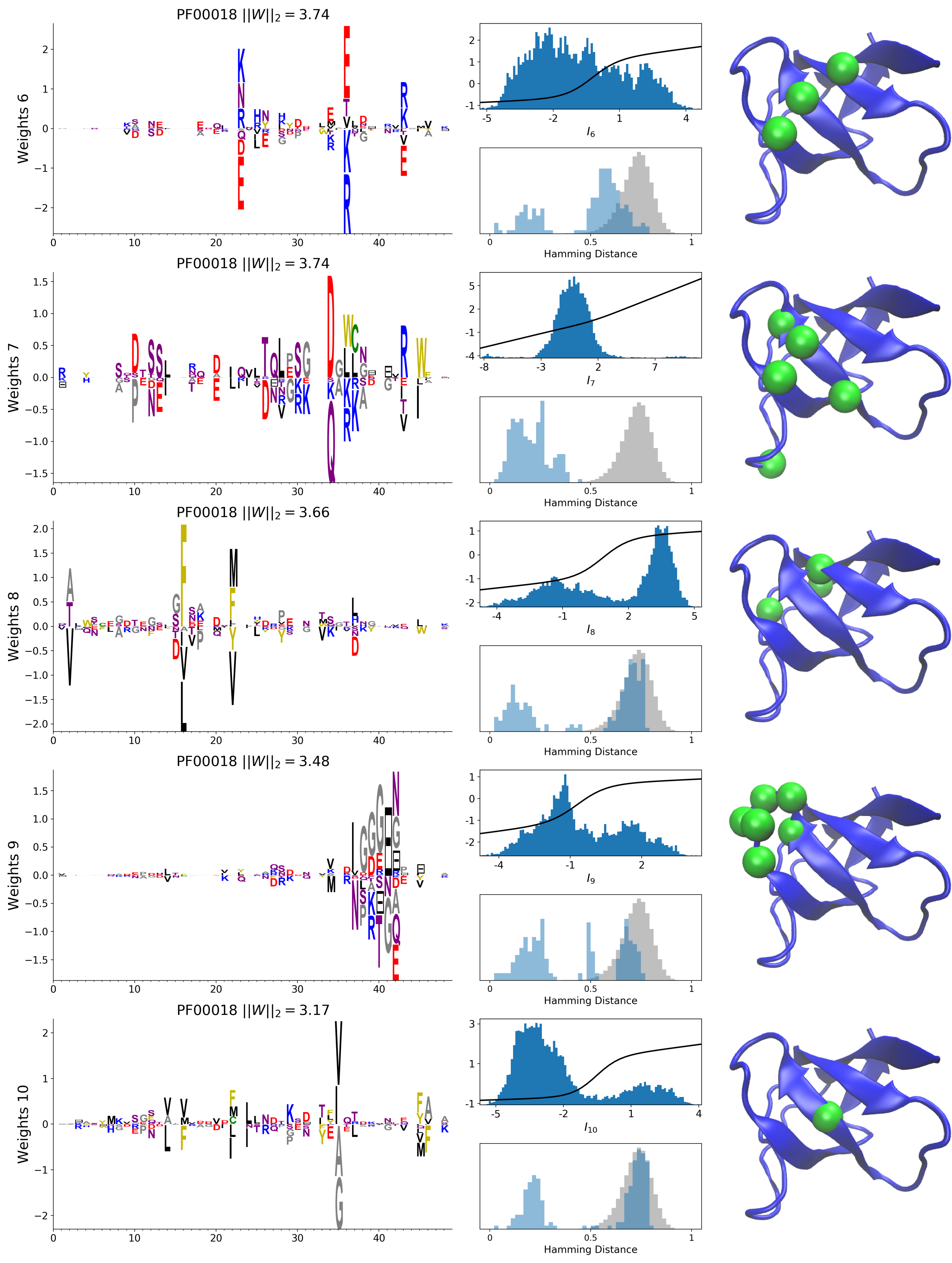

Supplement: Supplementary file 5. [file elife-39397-supp5.zip › Top_features_all/PF00018_top_features.pdf]

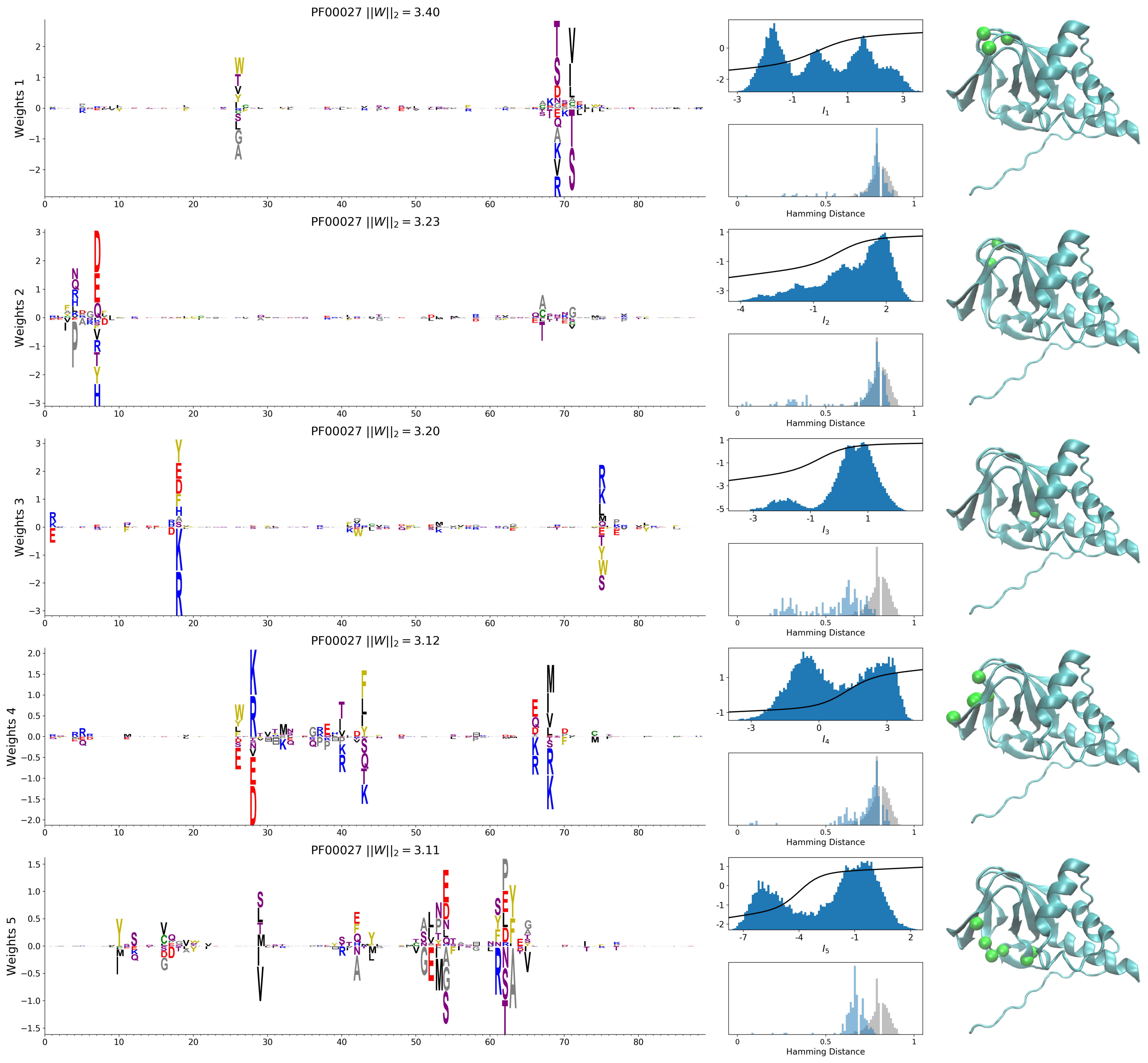

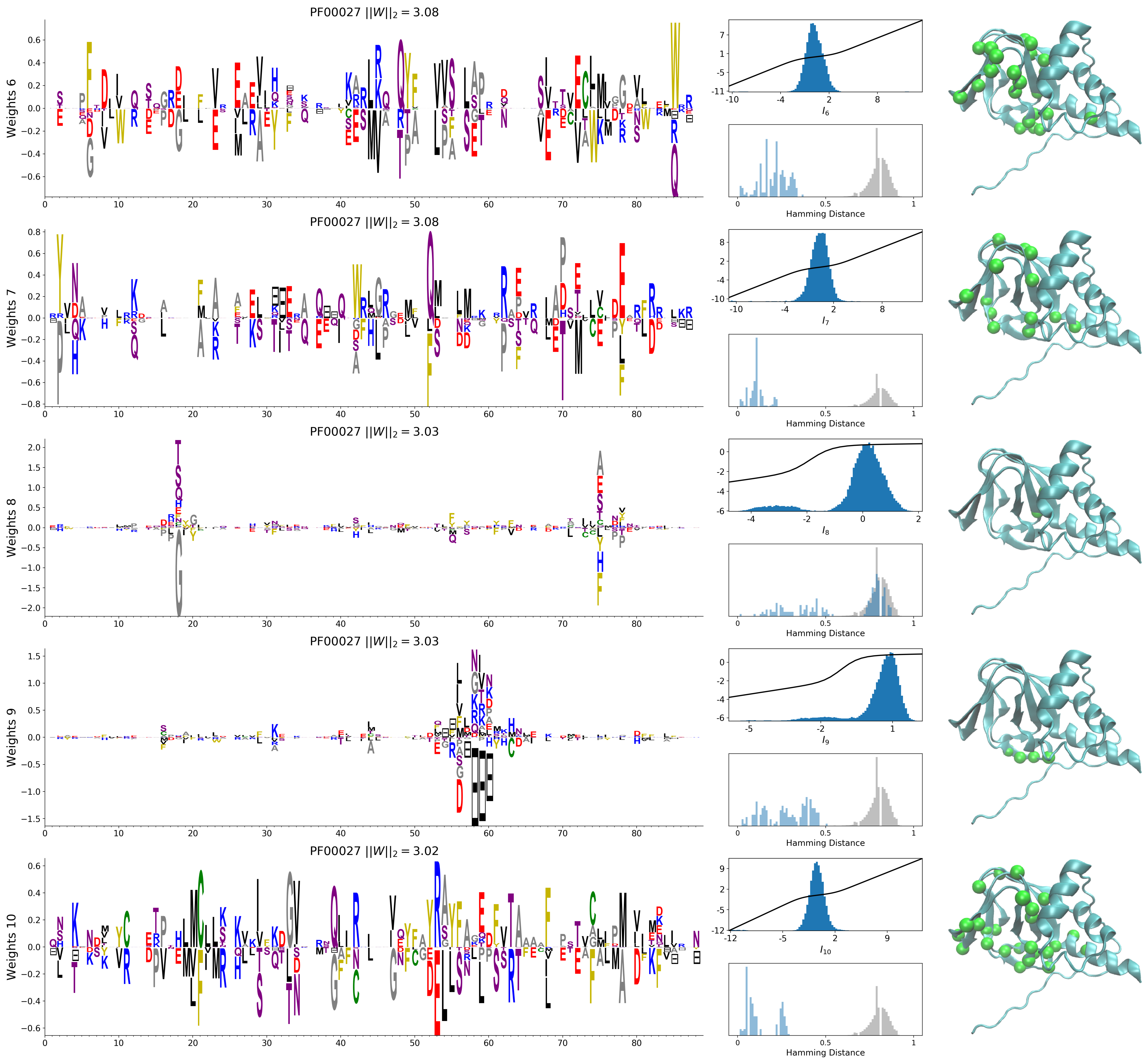

Supplement: Supplementary file 5. [file elife-39397-supp5.zip › Top_features_all/PF00027_top_features.pdf]

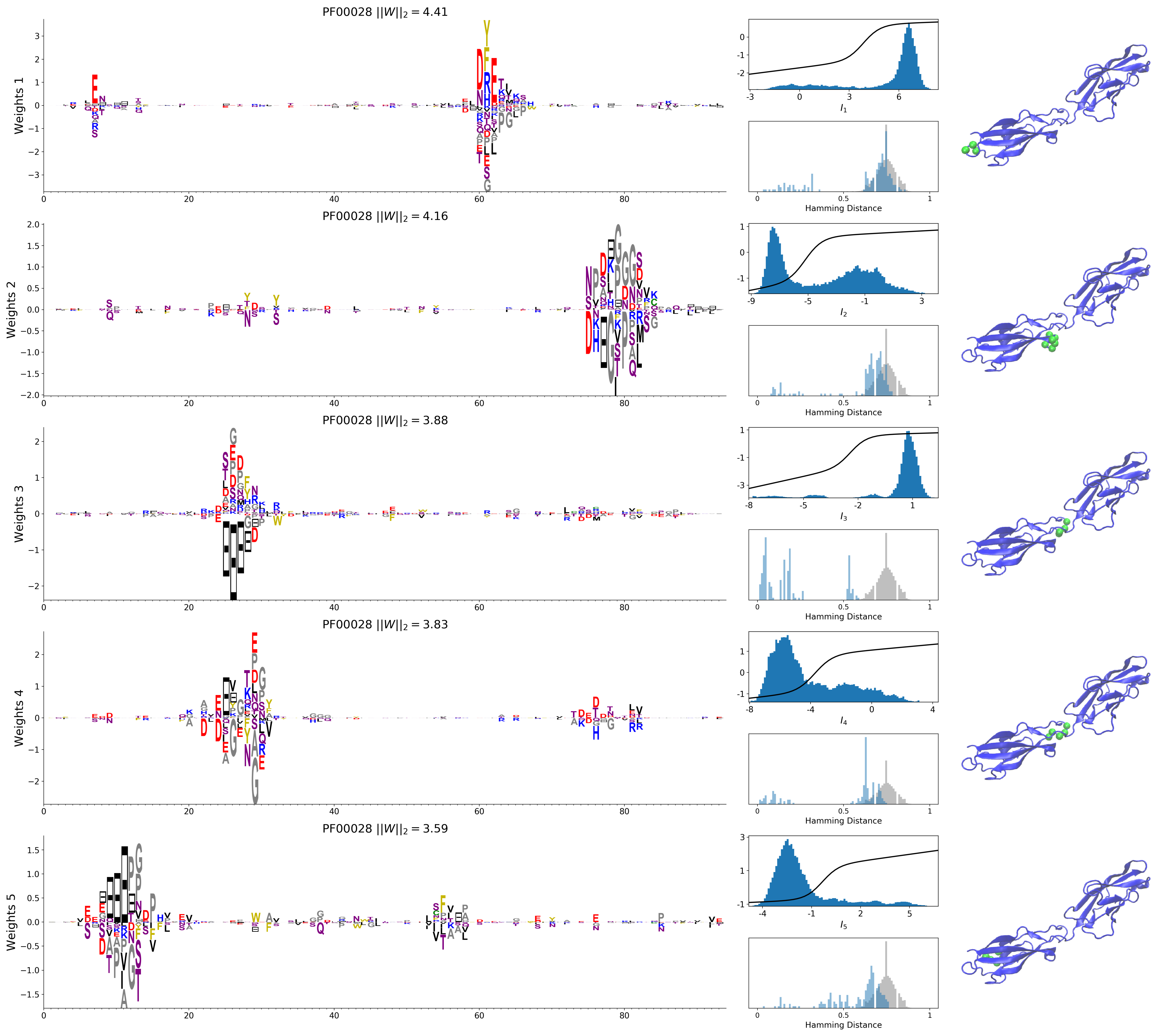

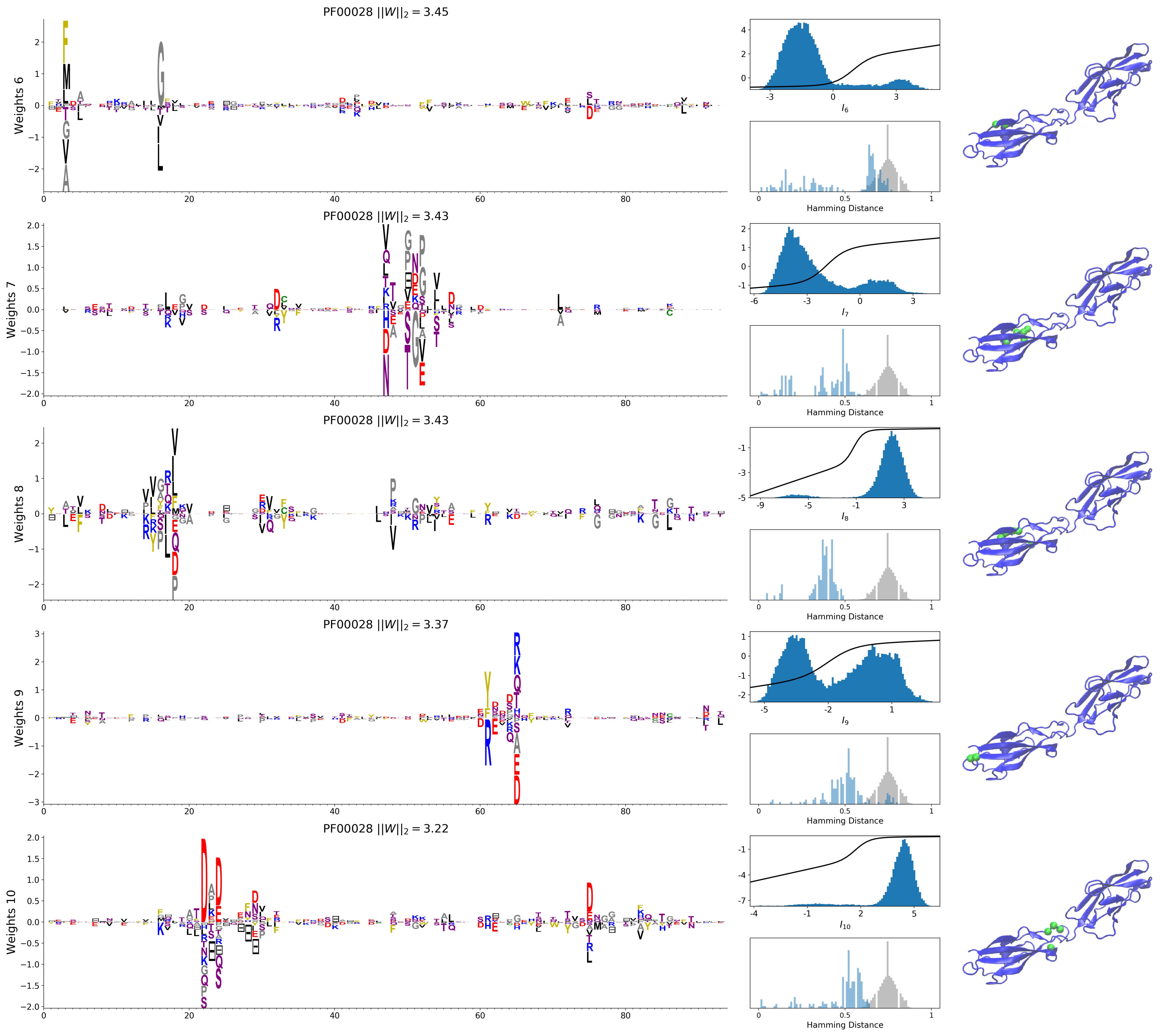

Supplement: Supplementary file 5. [file elife-39397-supp5.zip › Top_features_all/PF00028_top_features.pdf]

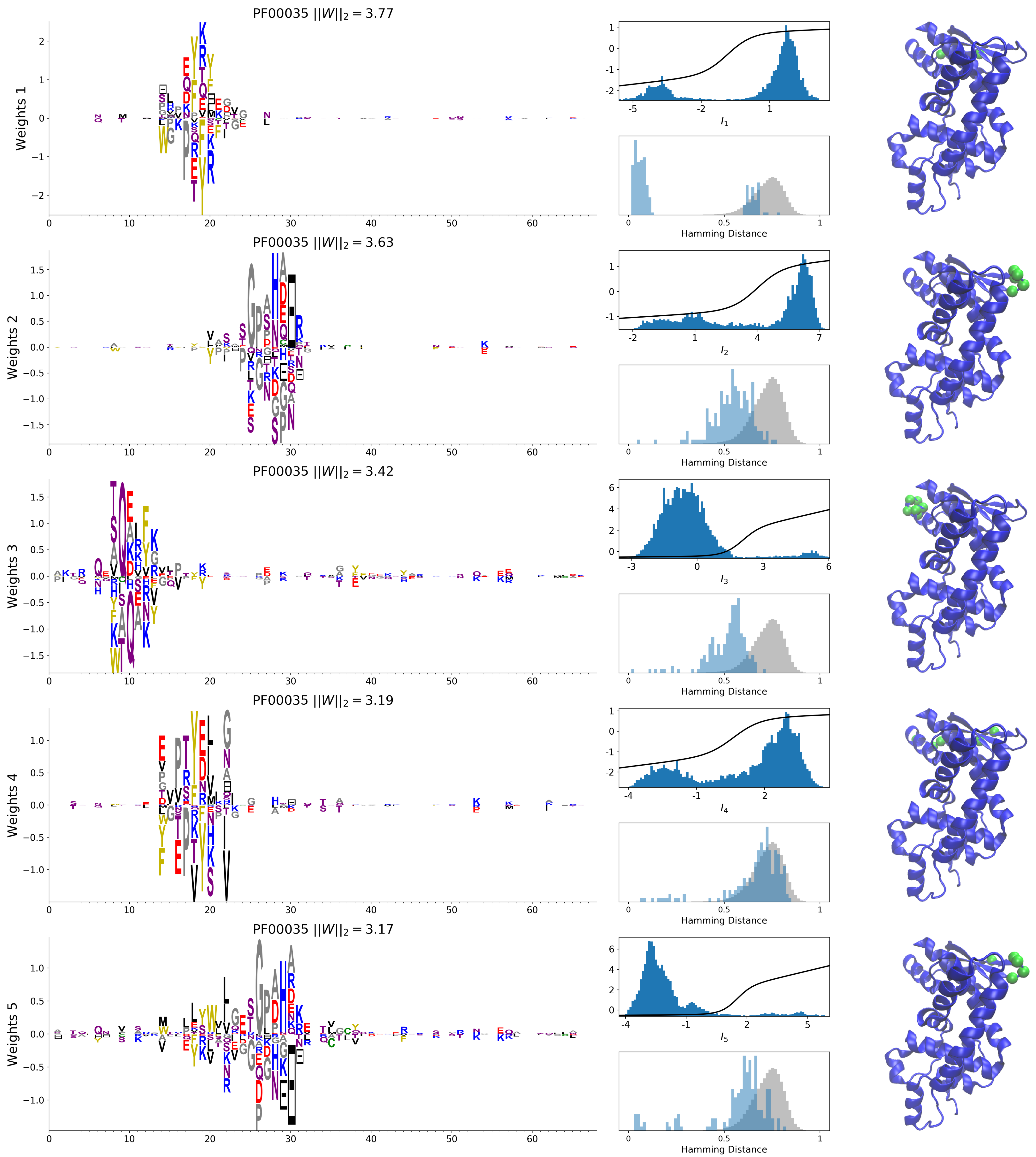

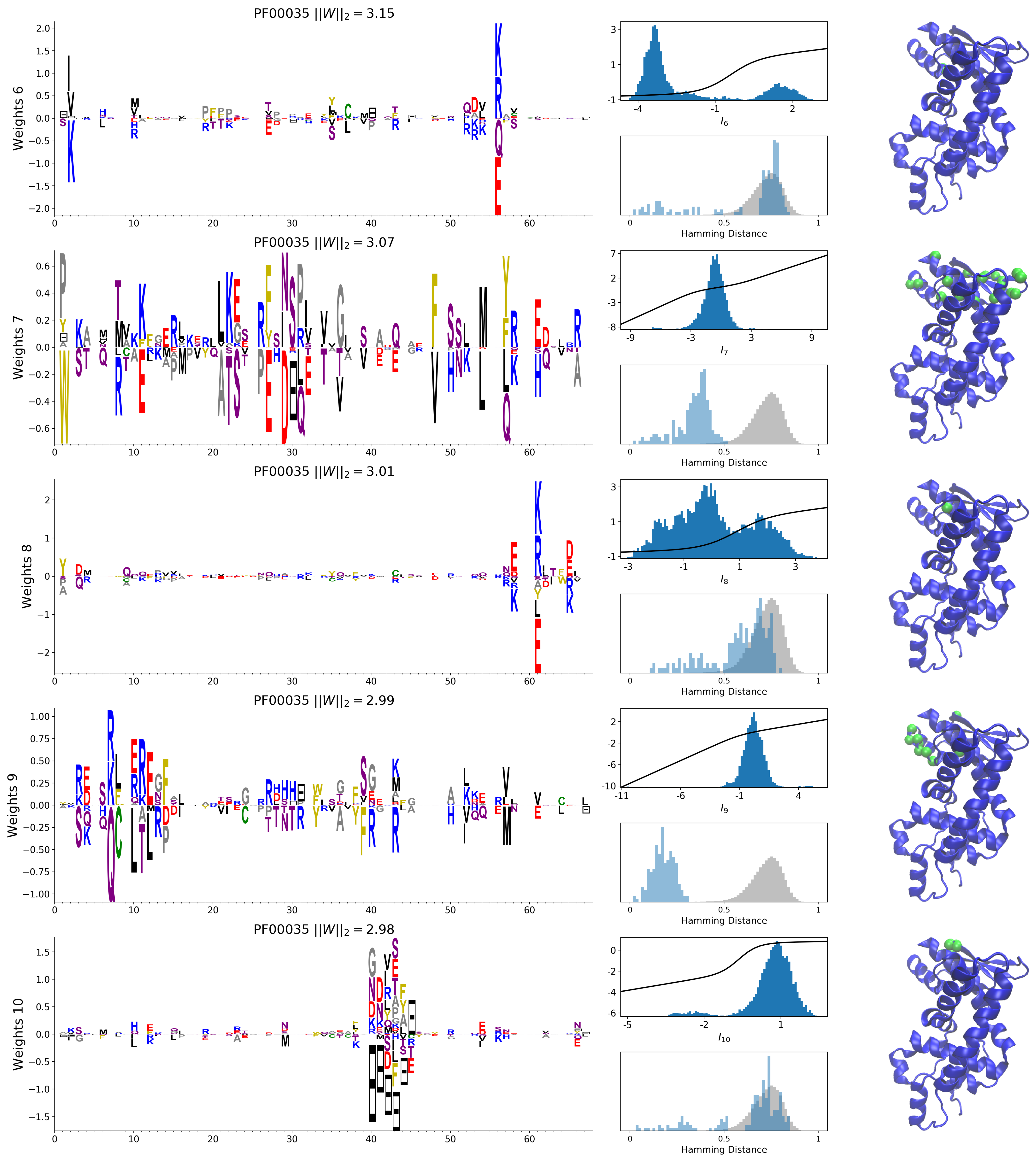

Supplement: Supplementary file 5. [file elife-39397-supp5.zip › Top_features_all/PF00035_top_features.pdf]

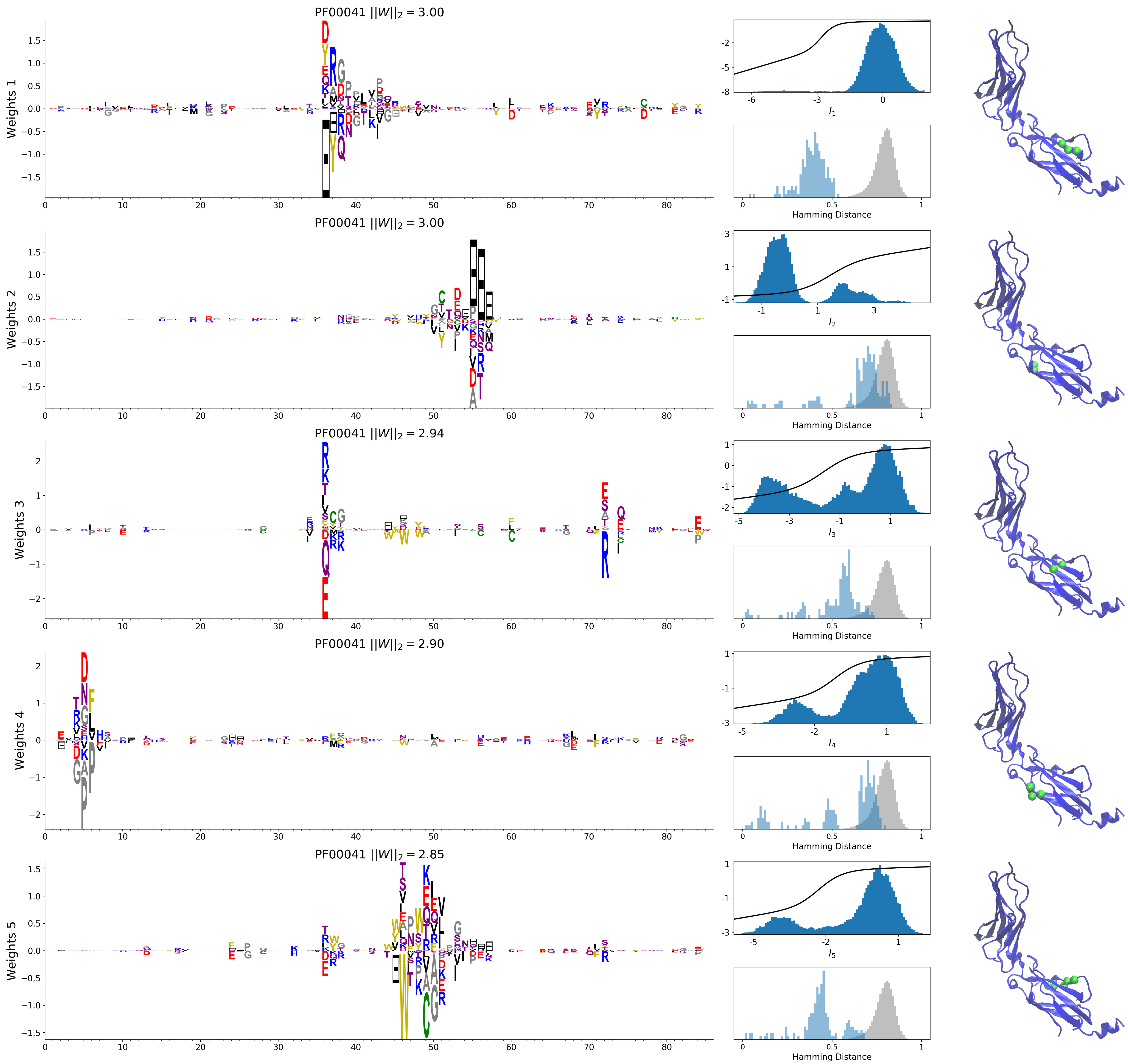

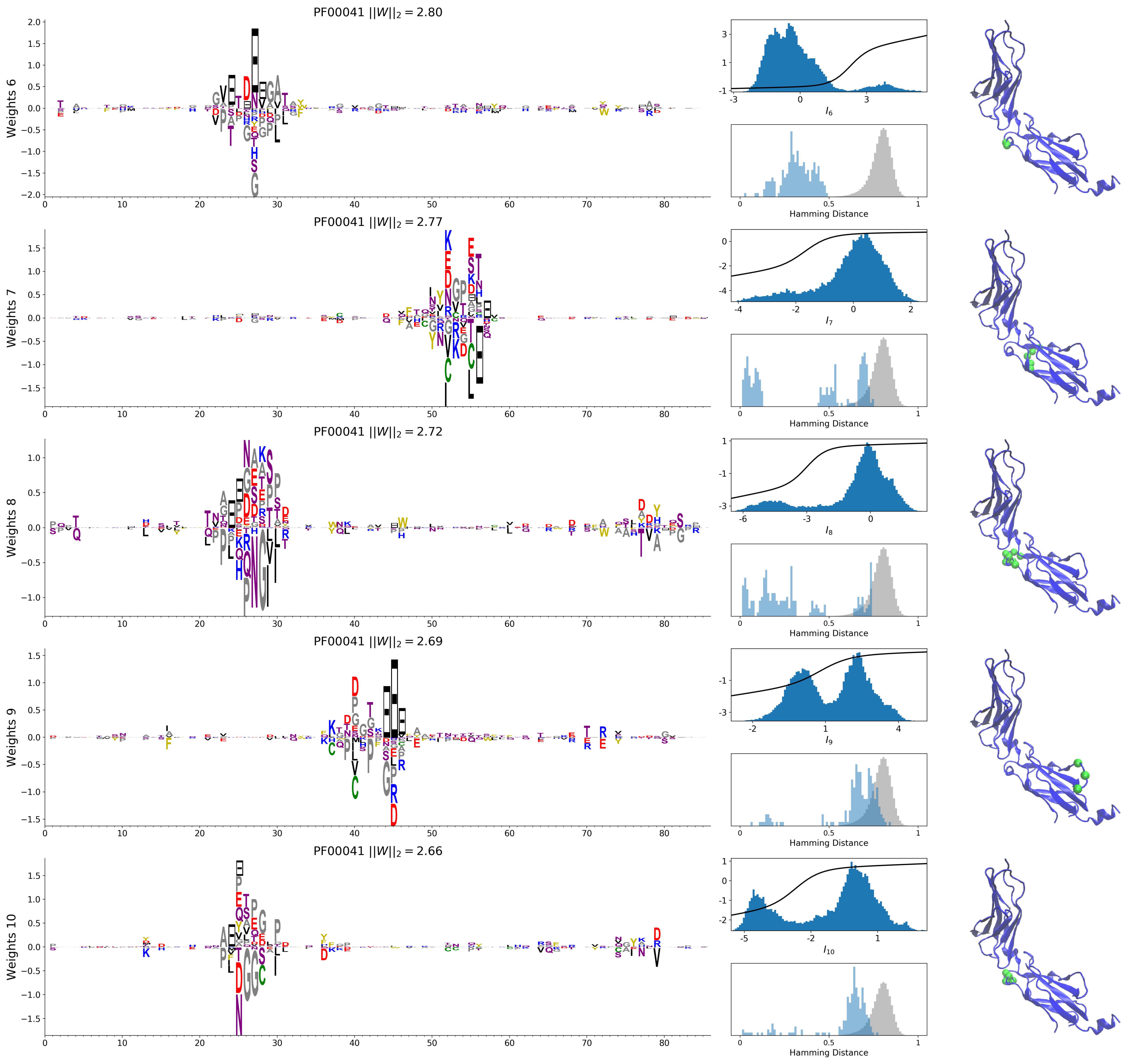

Supplement: Supplementary file 5. [file elife-39397-supp5.zip › Top_features_all/PF00041_top_features.pdf]

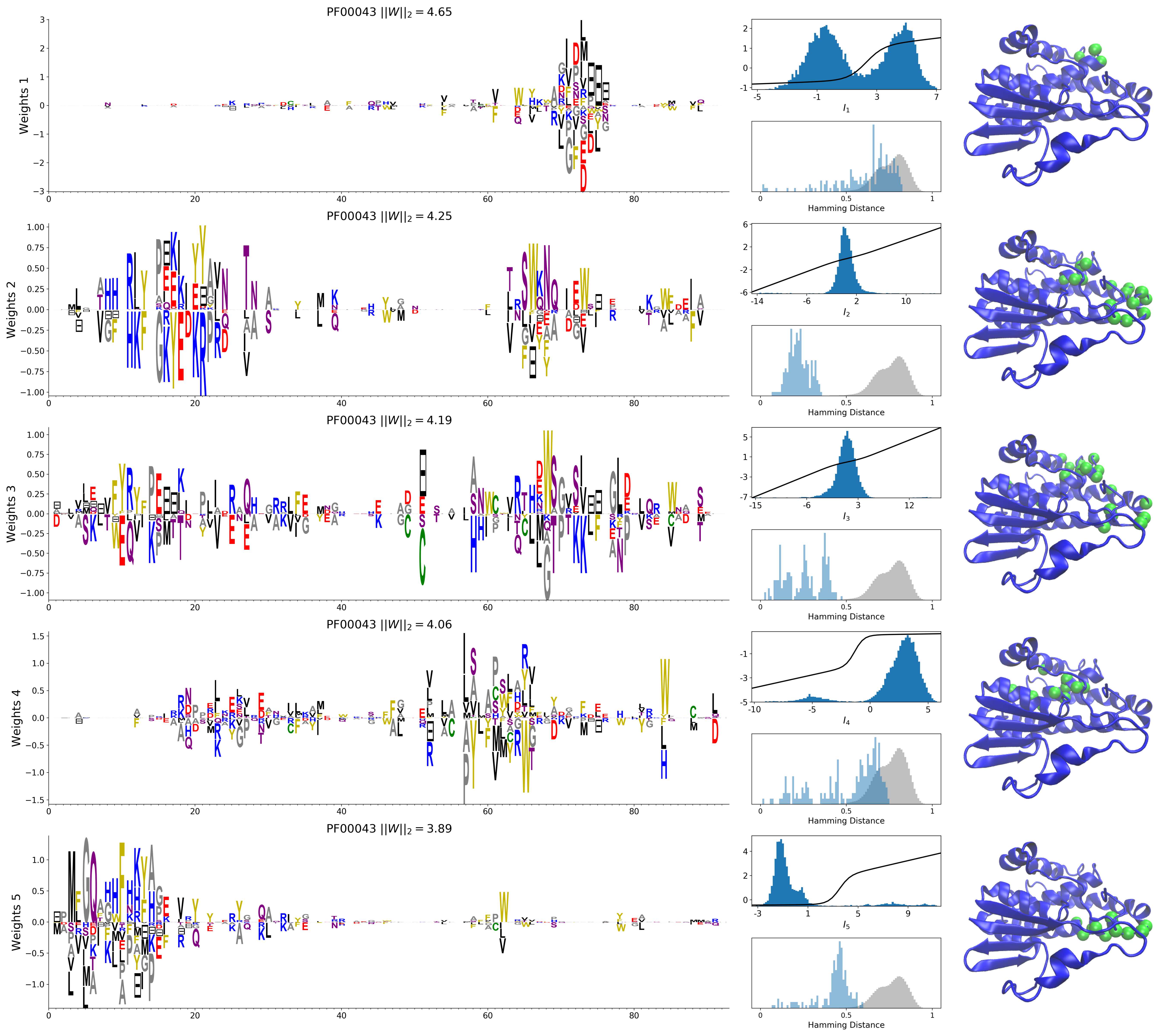

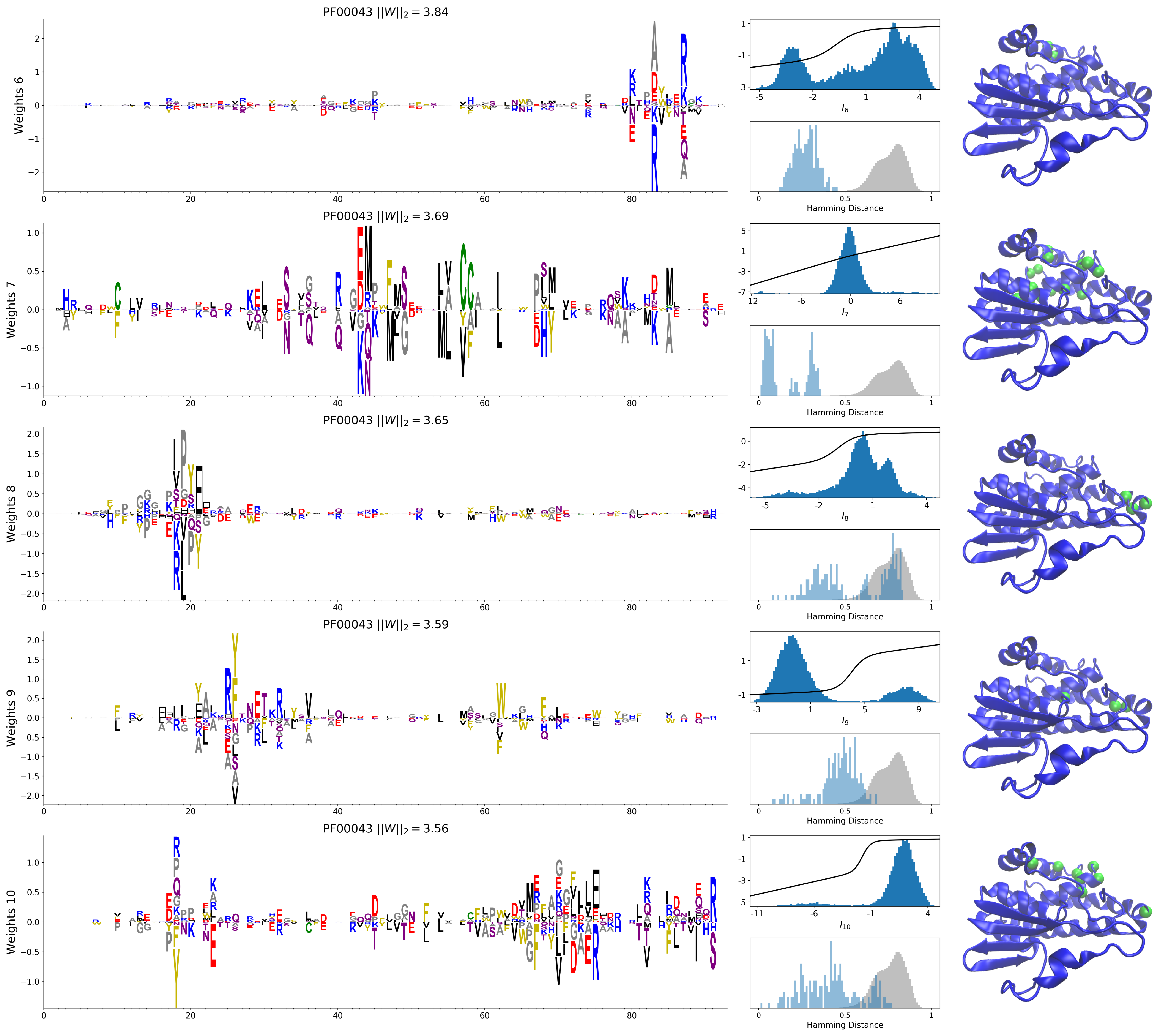

Supplement: Supplementary file 5. [file elife-39397-supp5.zip › Top_features_all/PF00043_top_features.pdf]

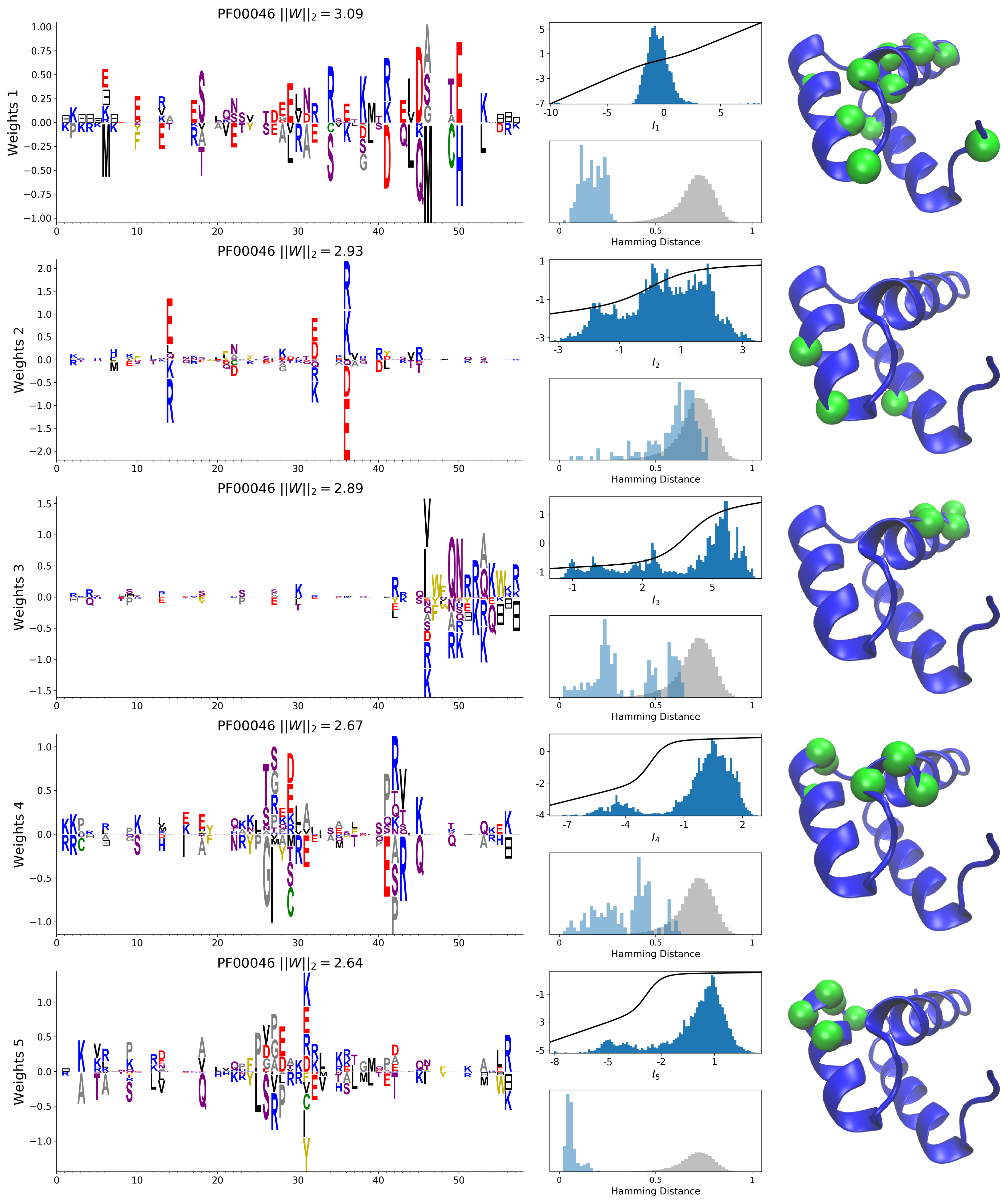

Supplement: Supplementary file 5. [file elife-39397-supp5.zip › Top_features_all/PF00046_top_features.pdf]

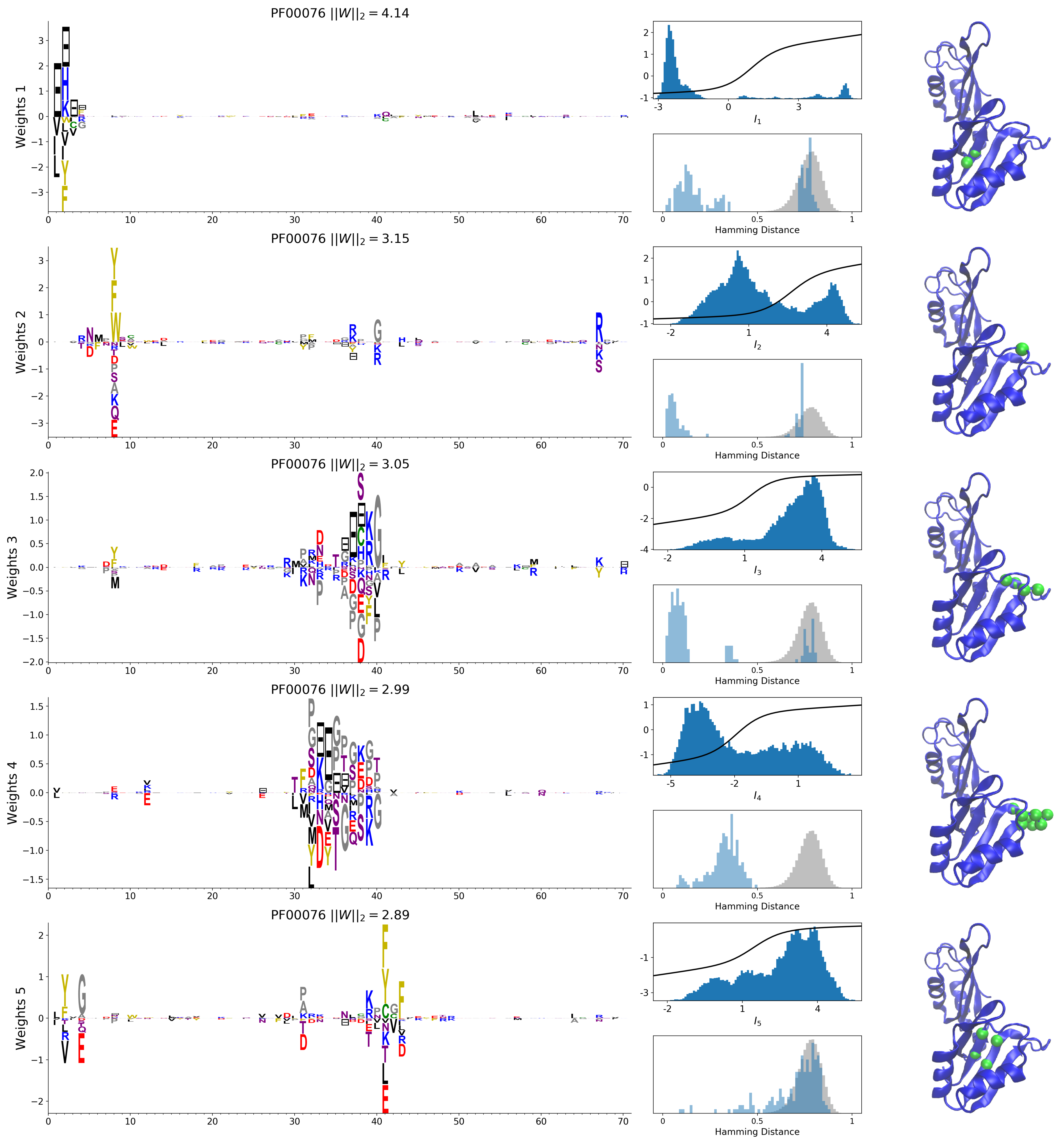

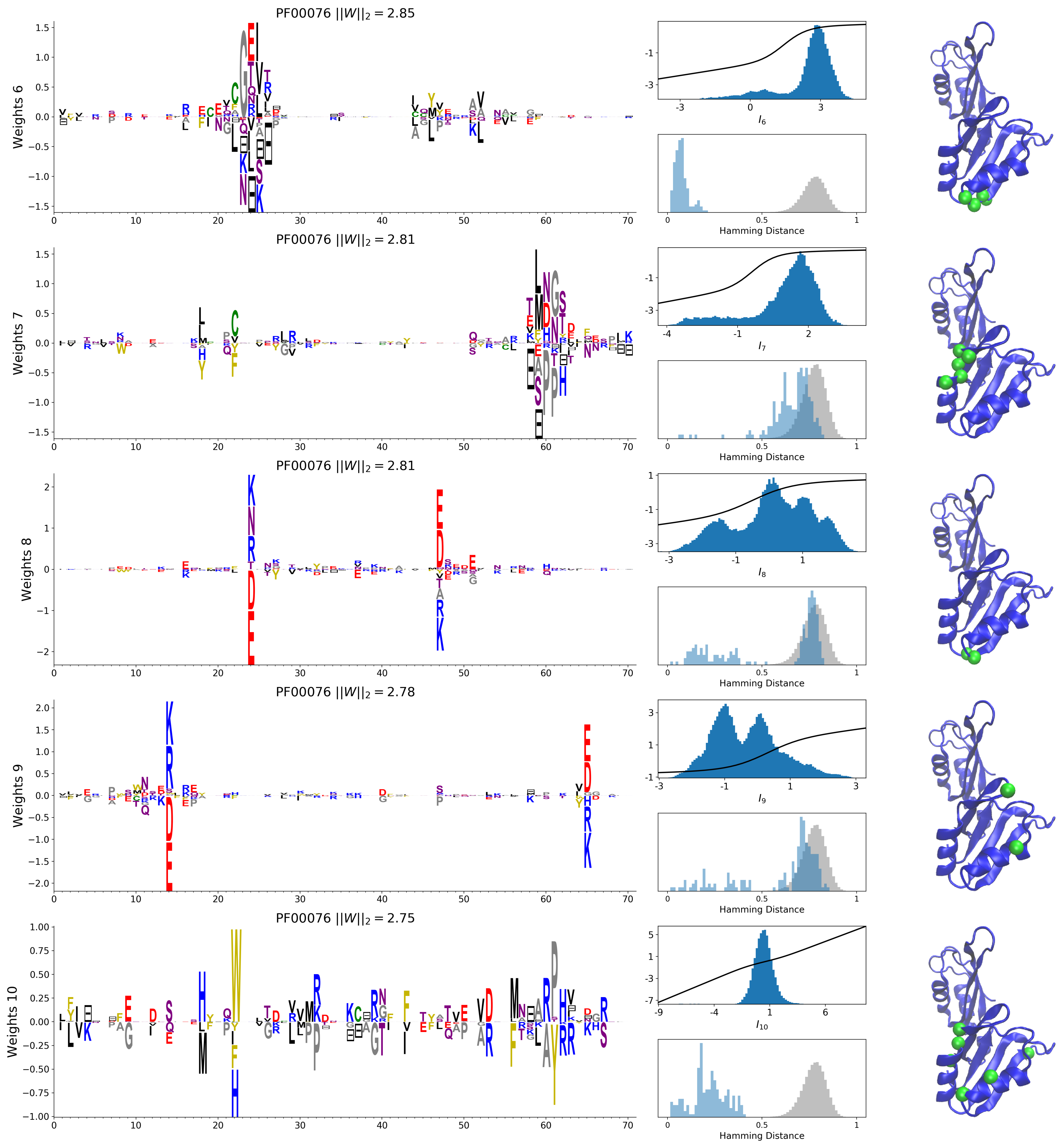

Supplement: Supplementary file 5. [file elife-39397-supp5.zip › Top_features_all/PF00076_top_features.pdf]

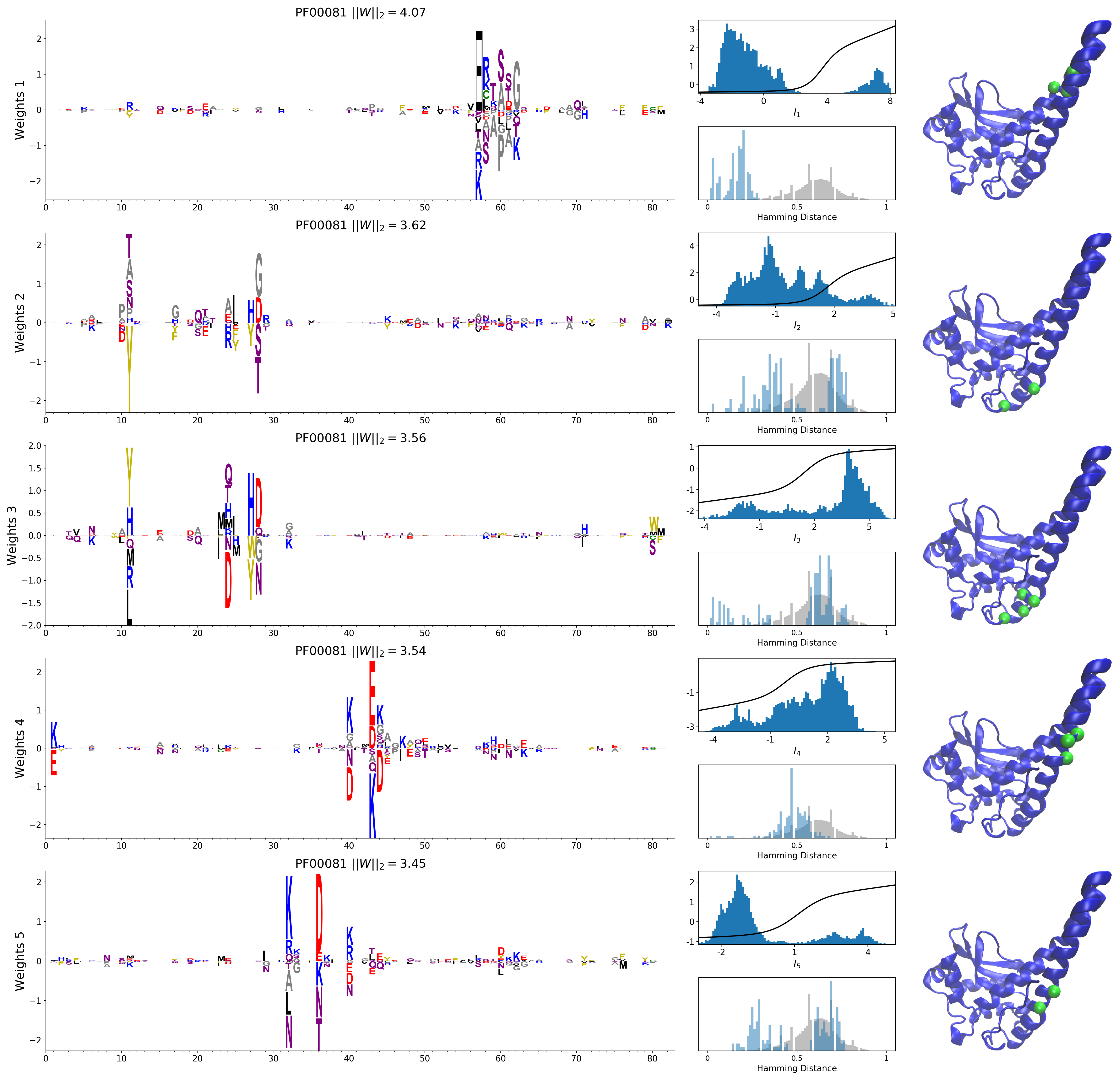

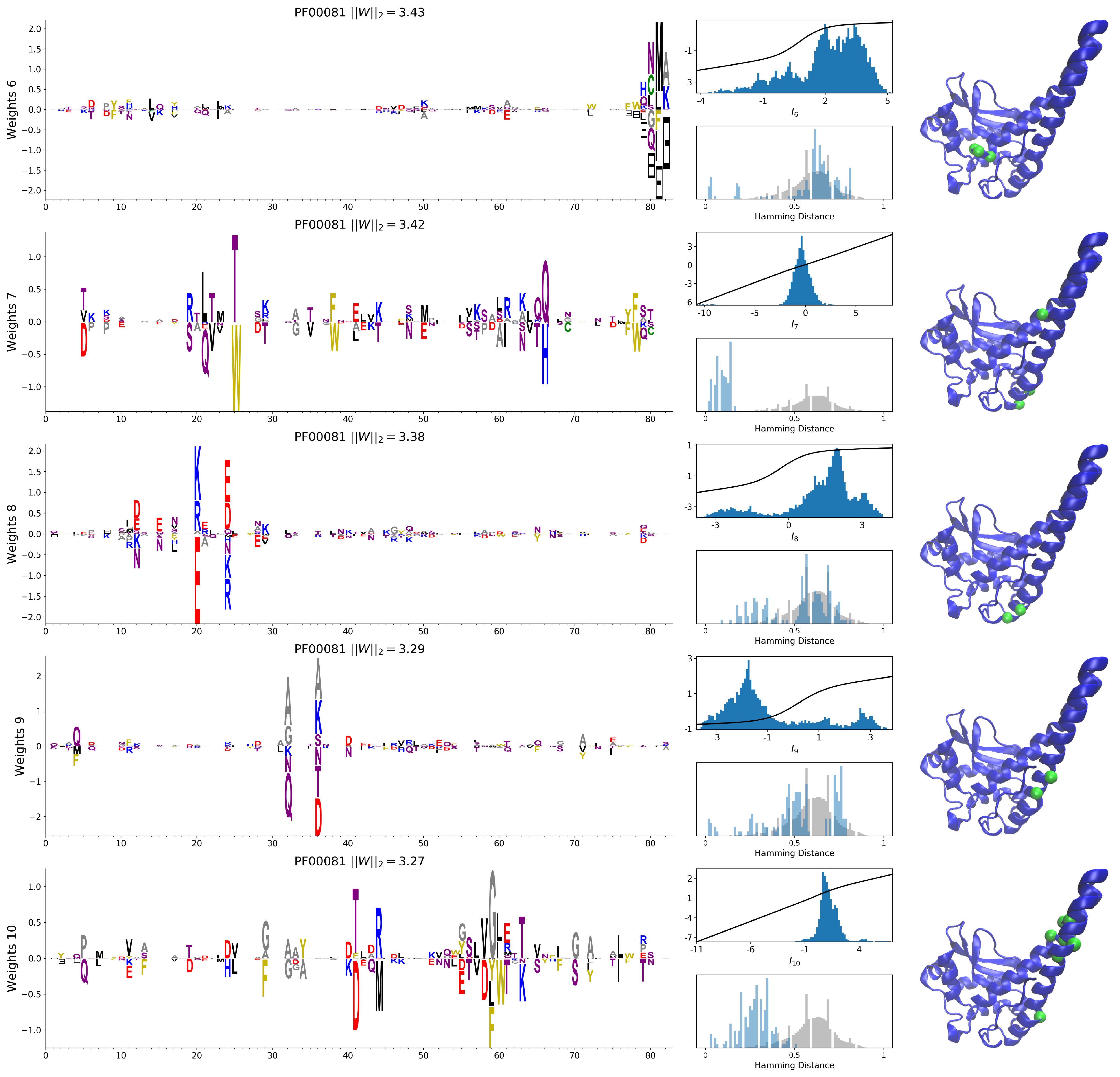

Supplement: Supplementary file 5. [file elife-39397-supp5.zip › Top_features_all/PF00081_top_features.pdf]

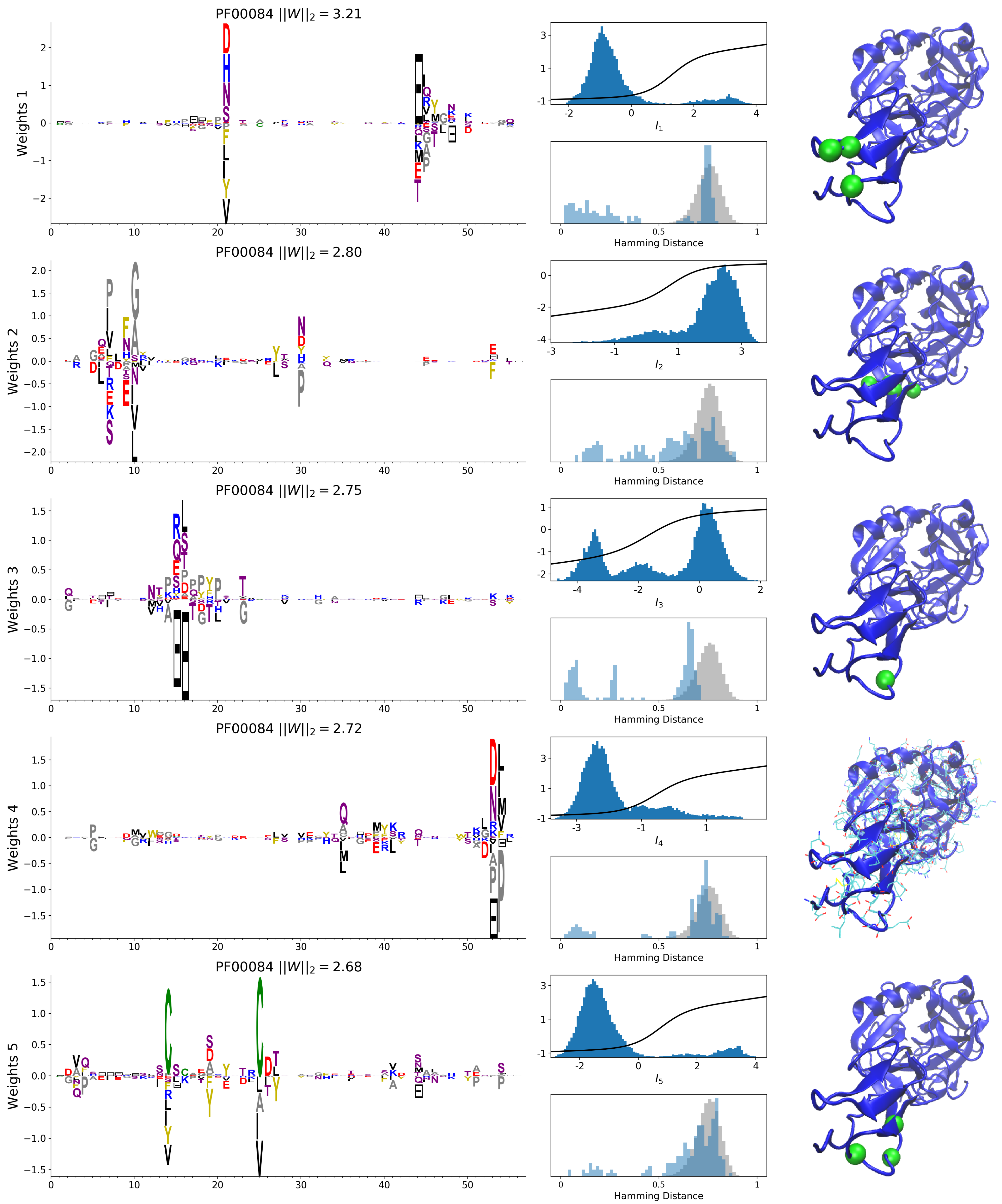

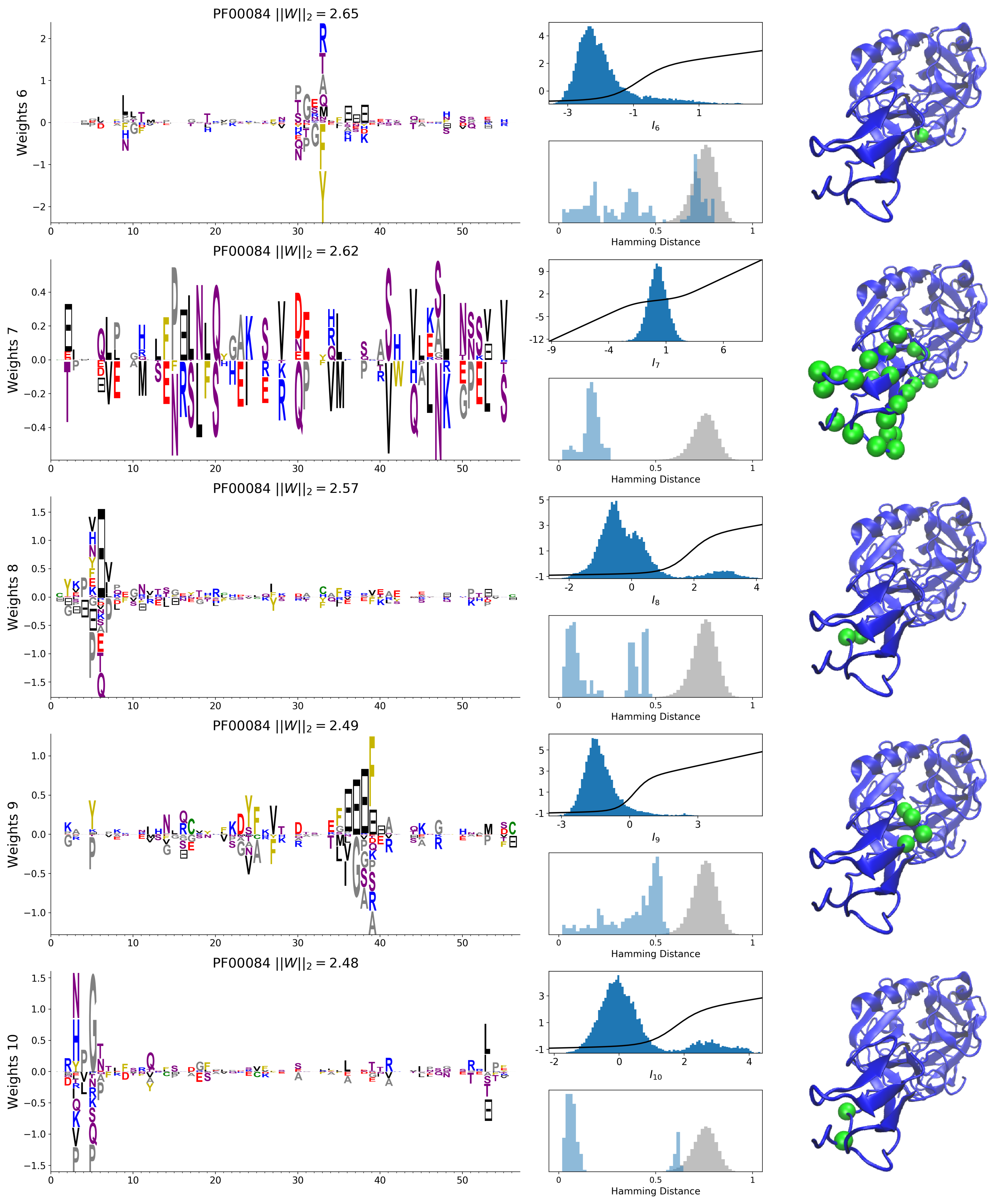

Supplement: Supplementary file 5. [file elife-39397-supp5.zip › Top_features_all/PF00084_top_features.pdf]

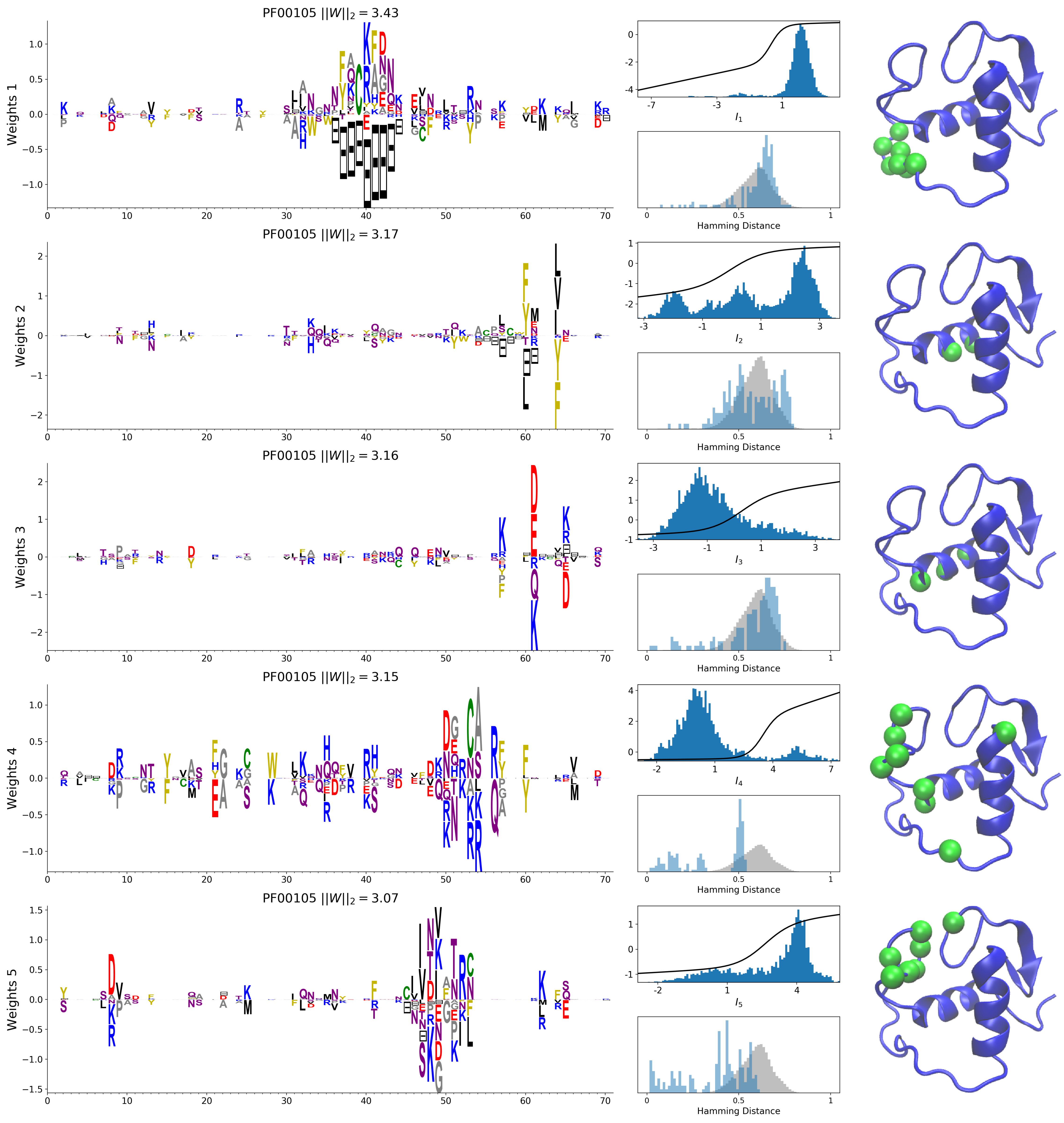

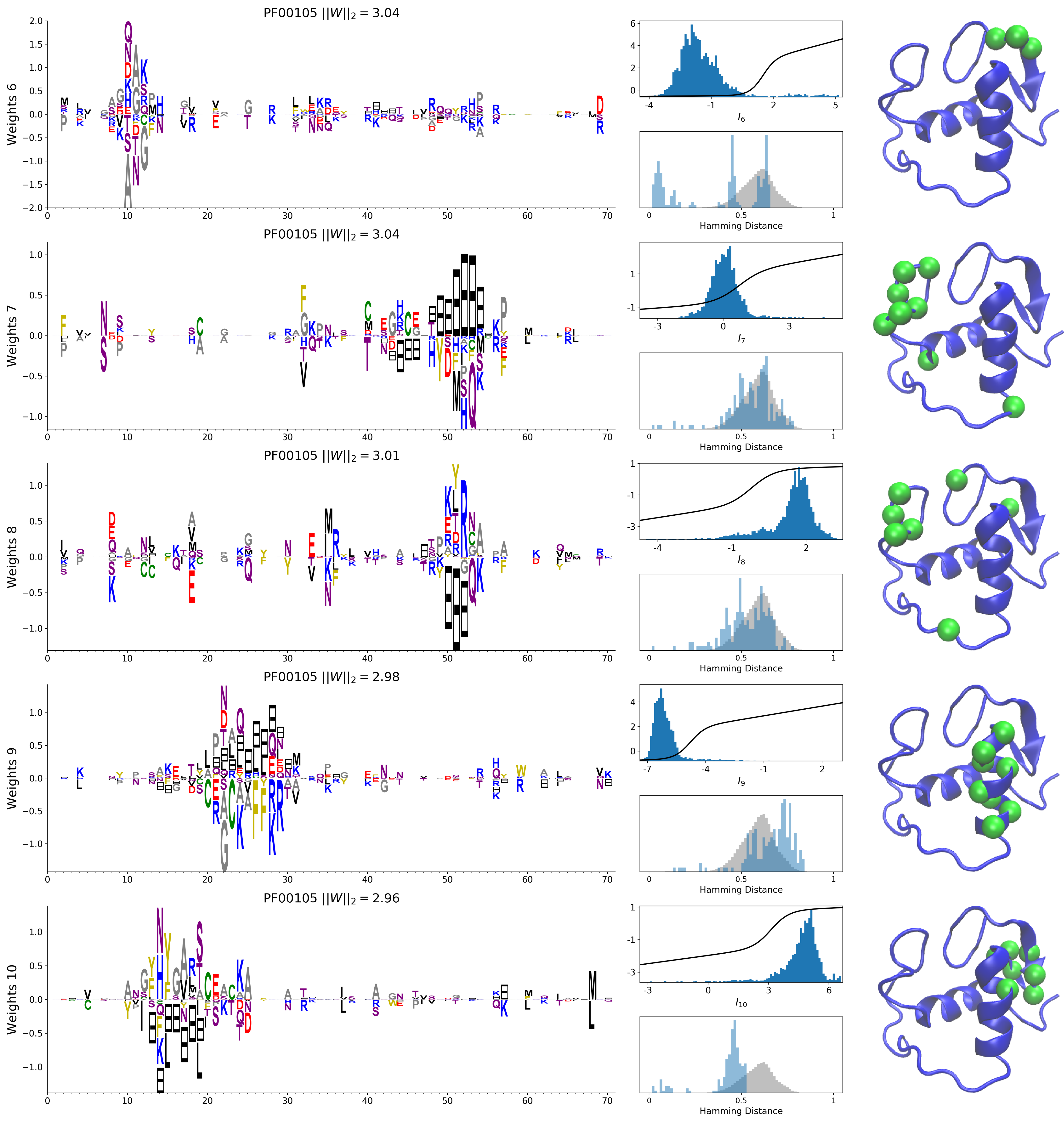

Supplement: Supplementary file 5. [file elife-39397-supp5.zip › Top_features_all/PF00105_top_features.pdf]

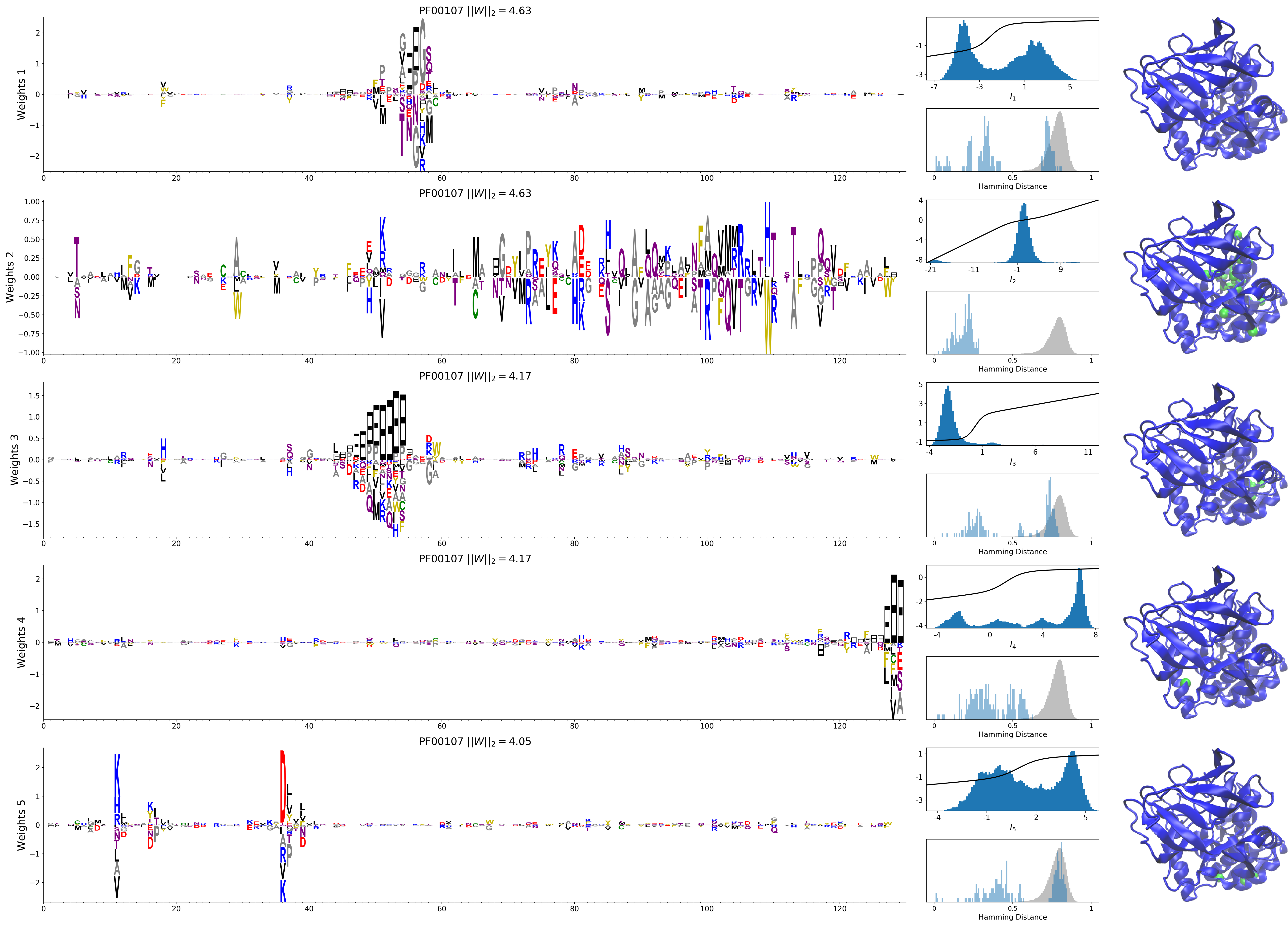

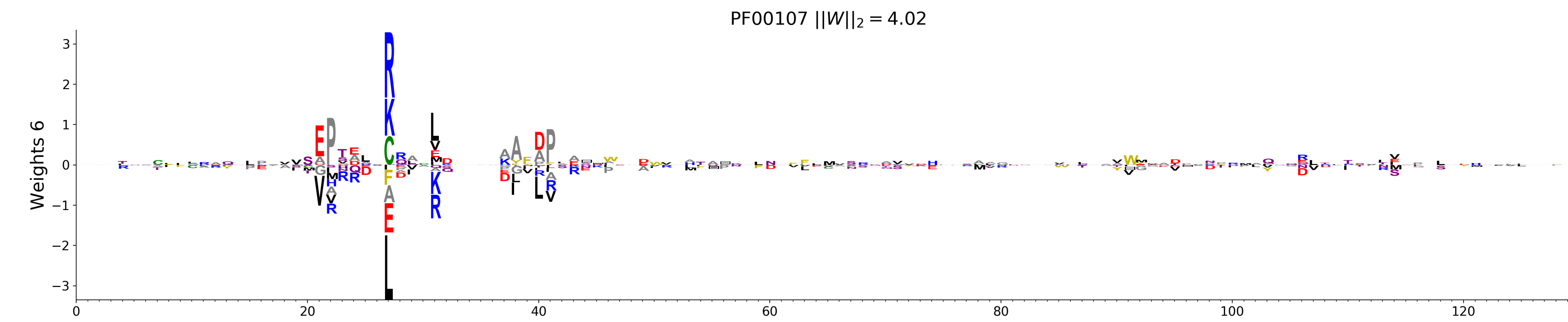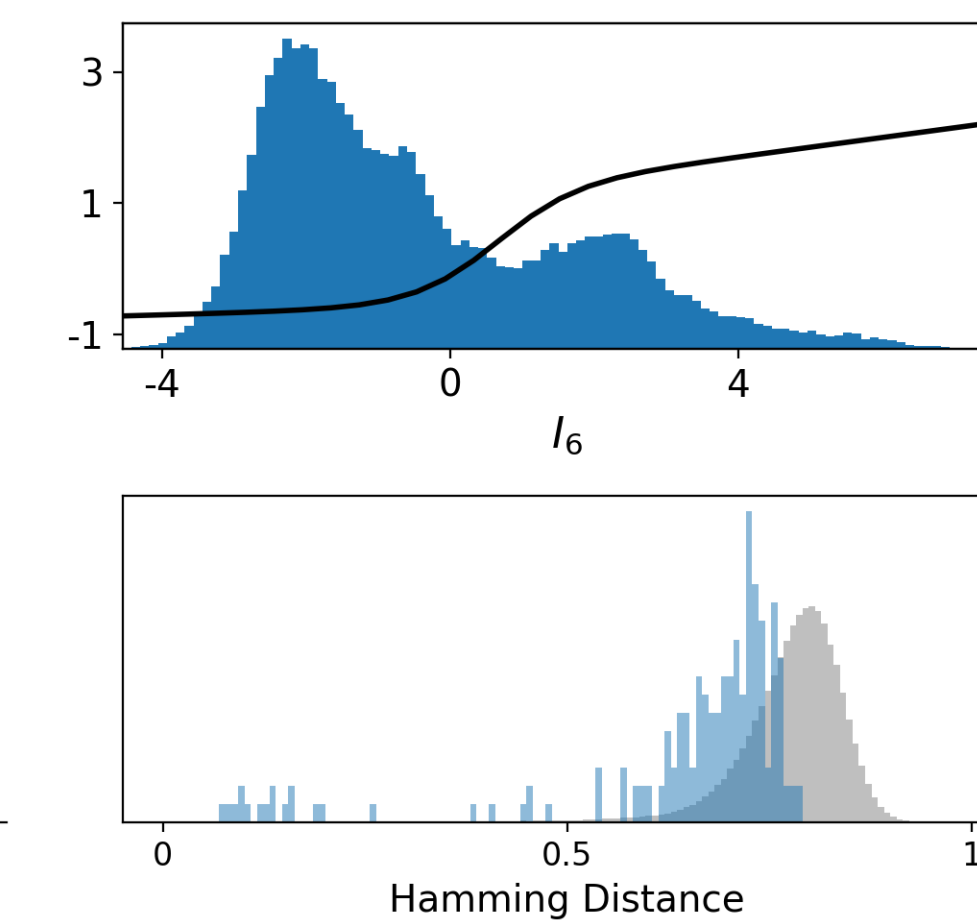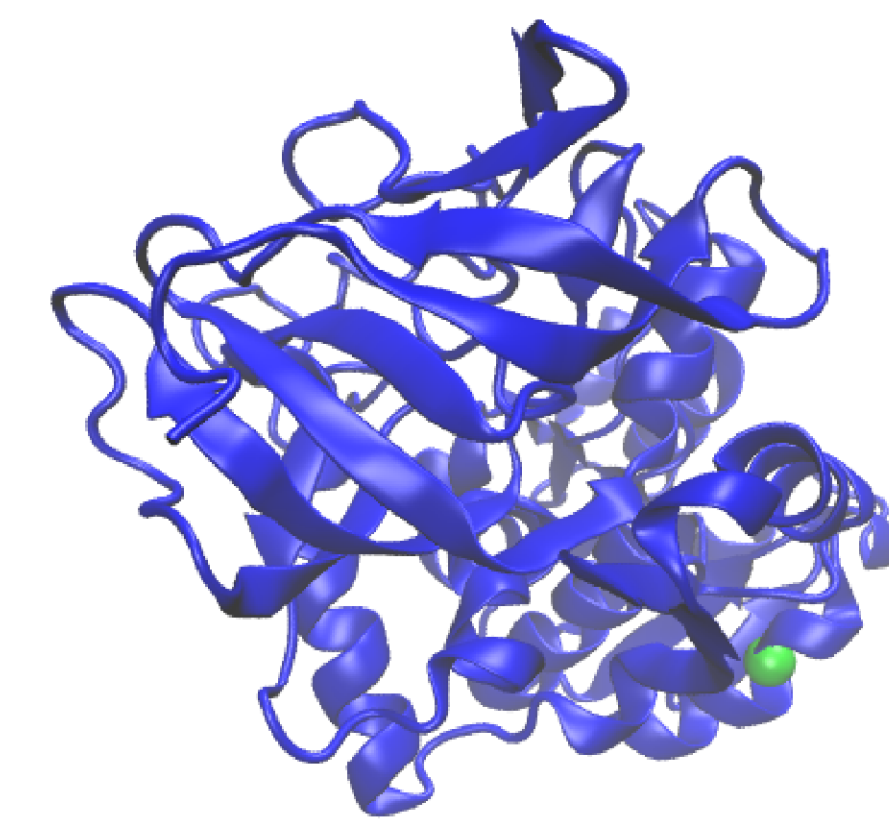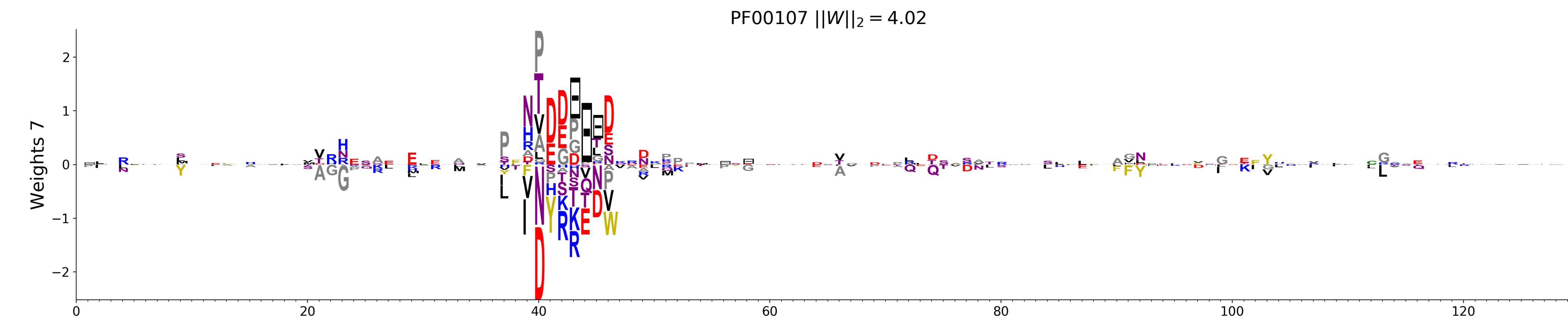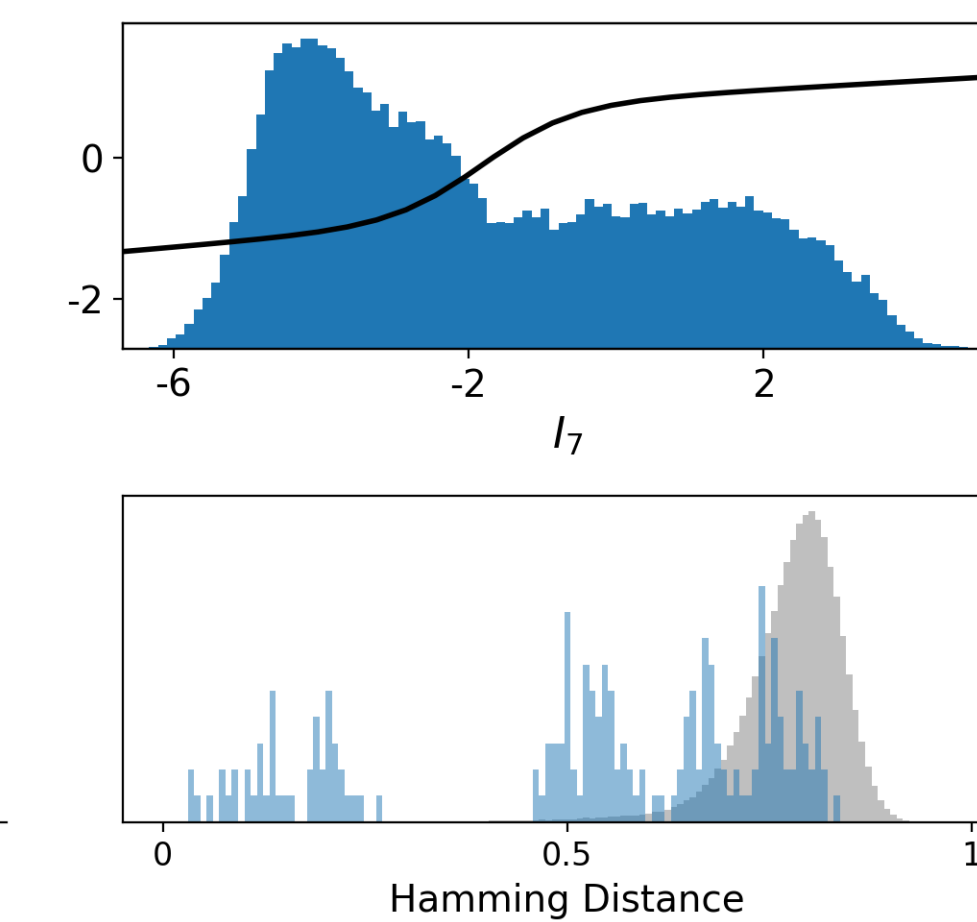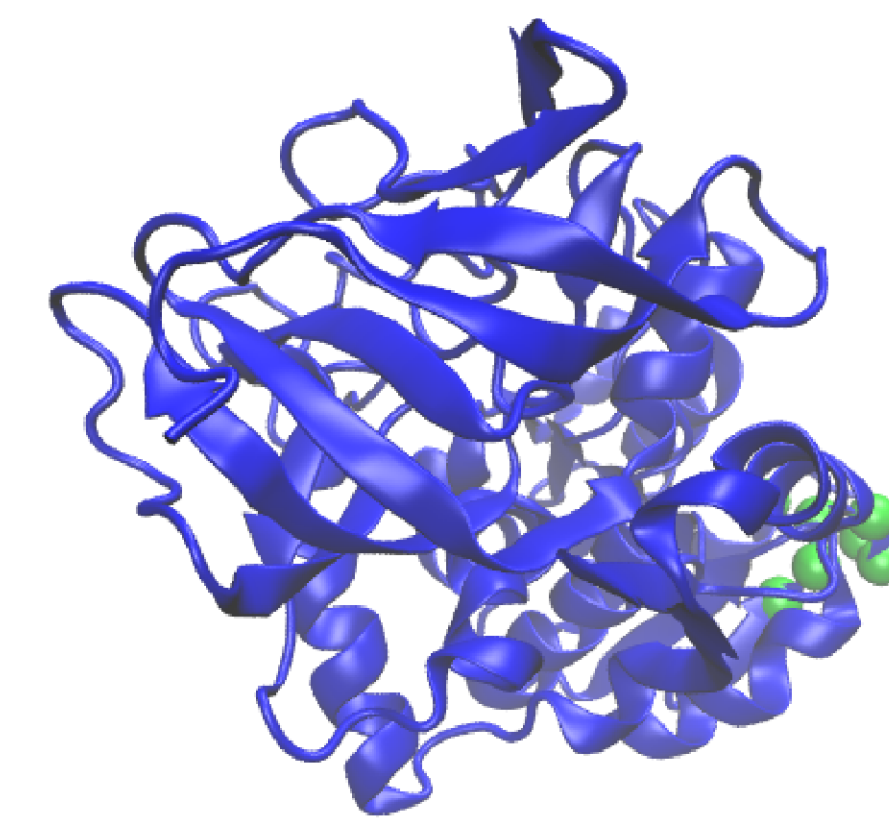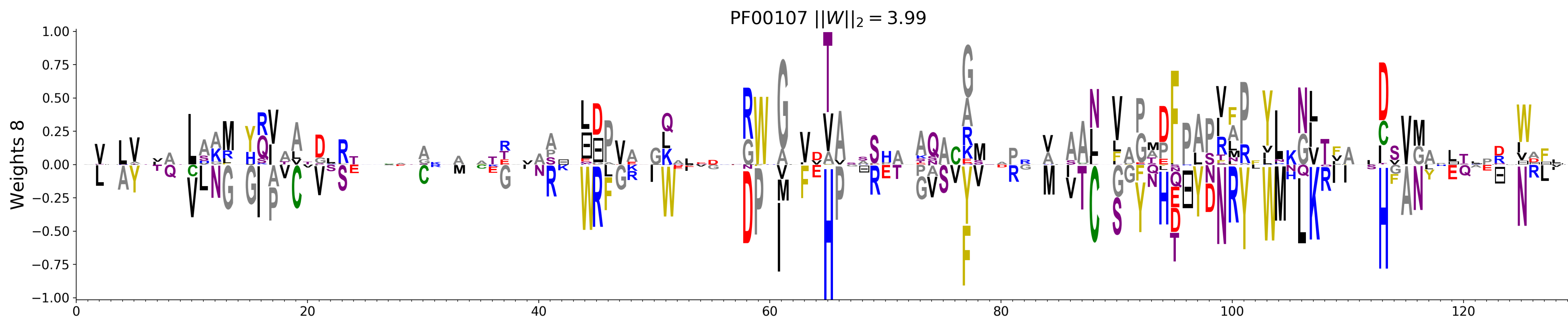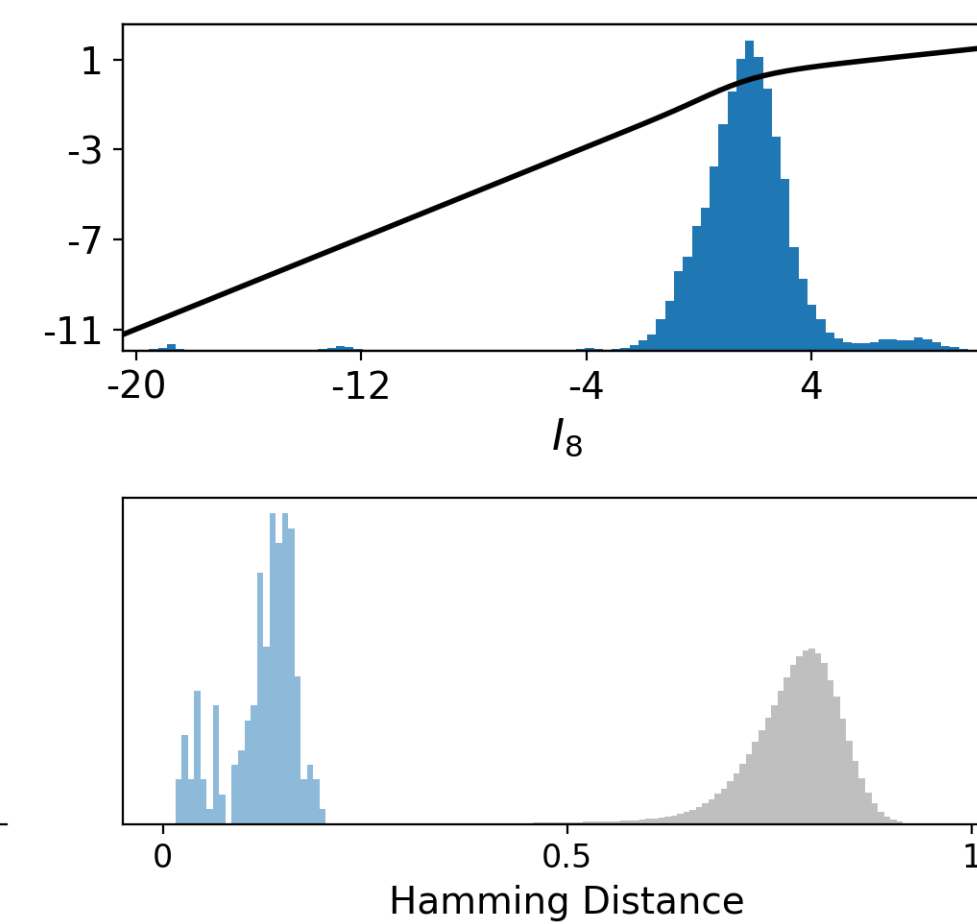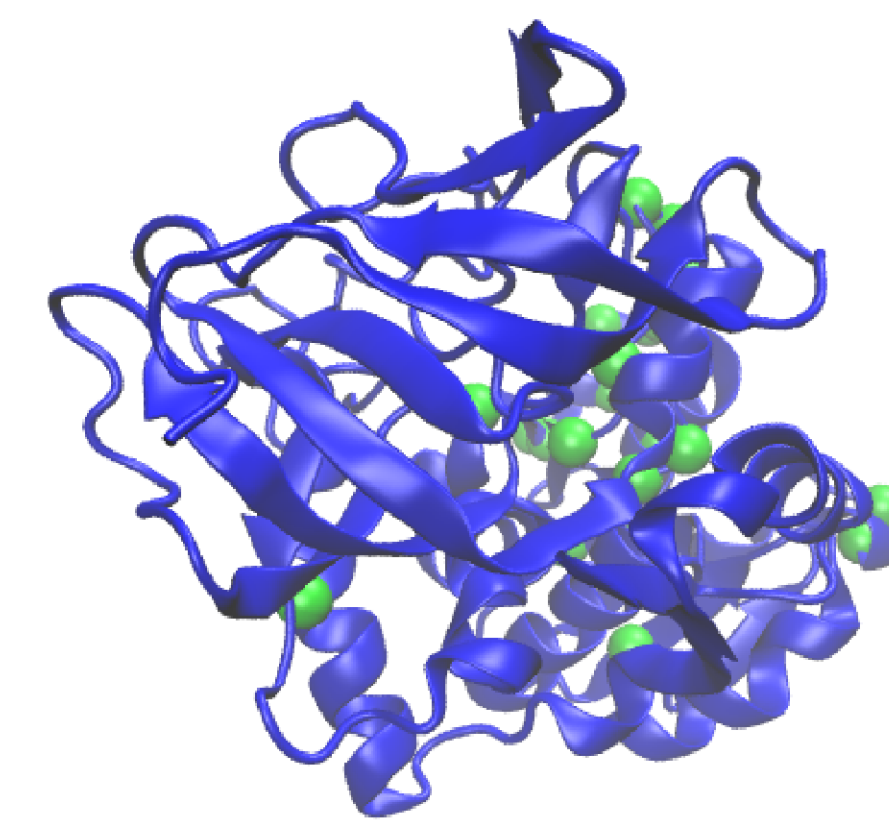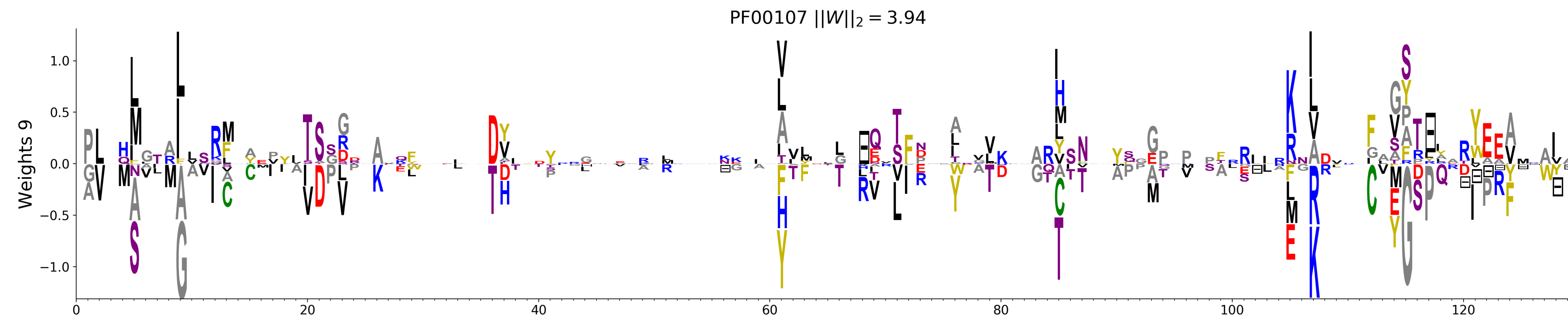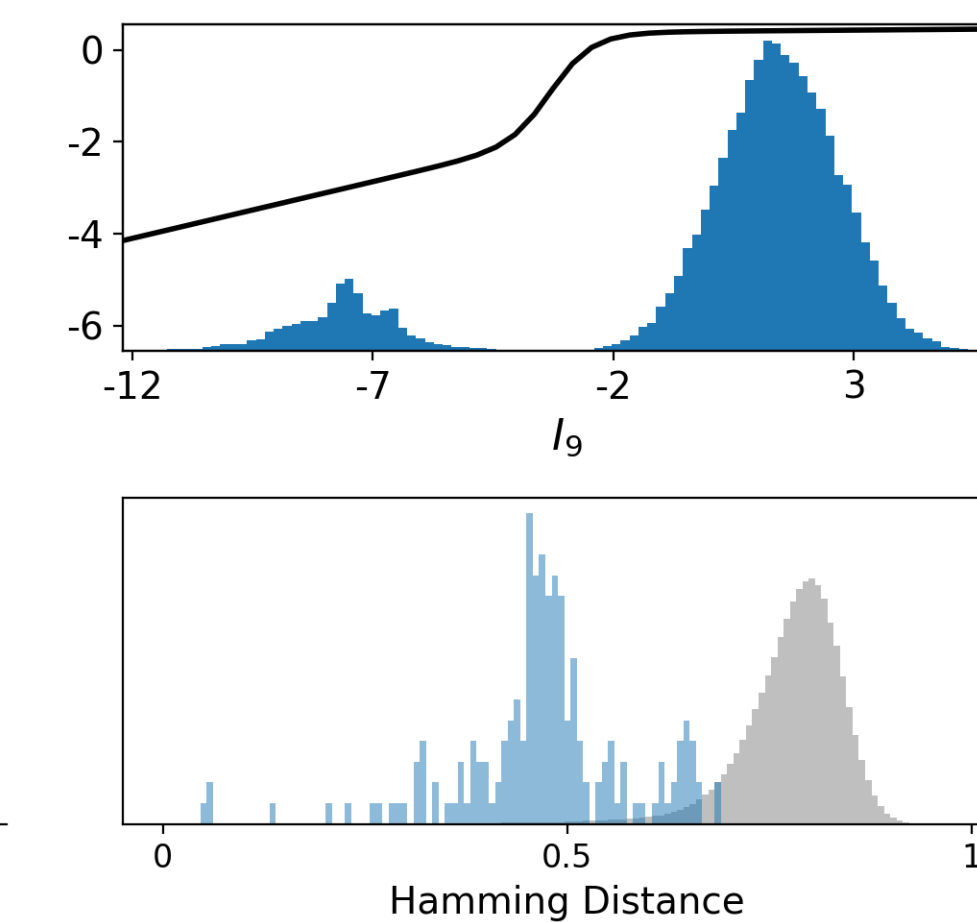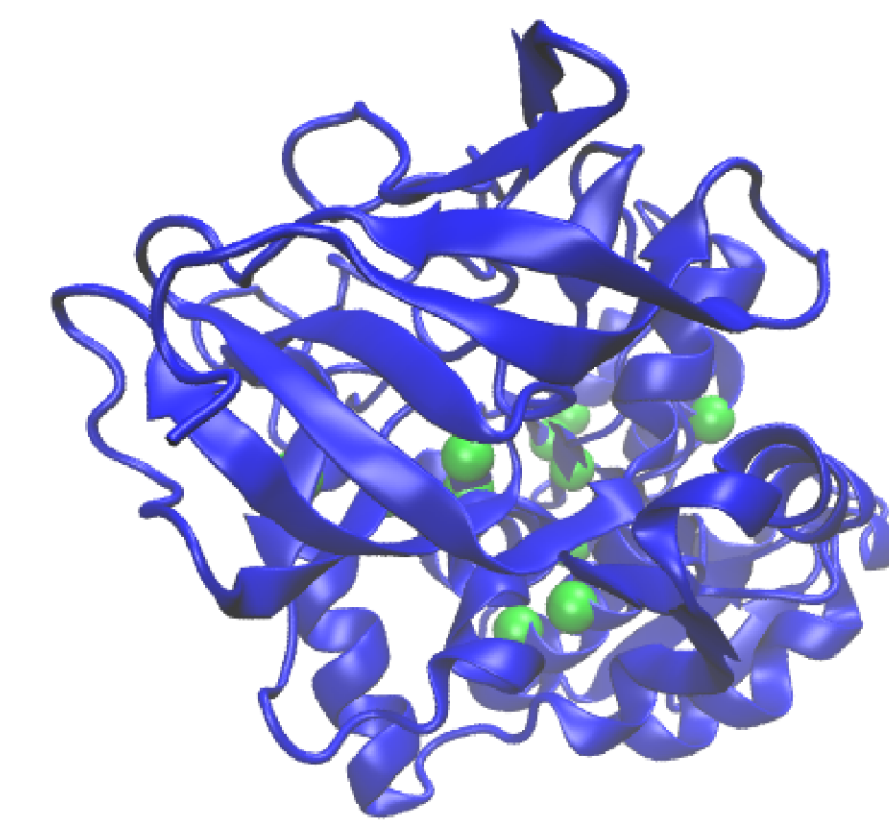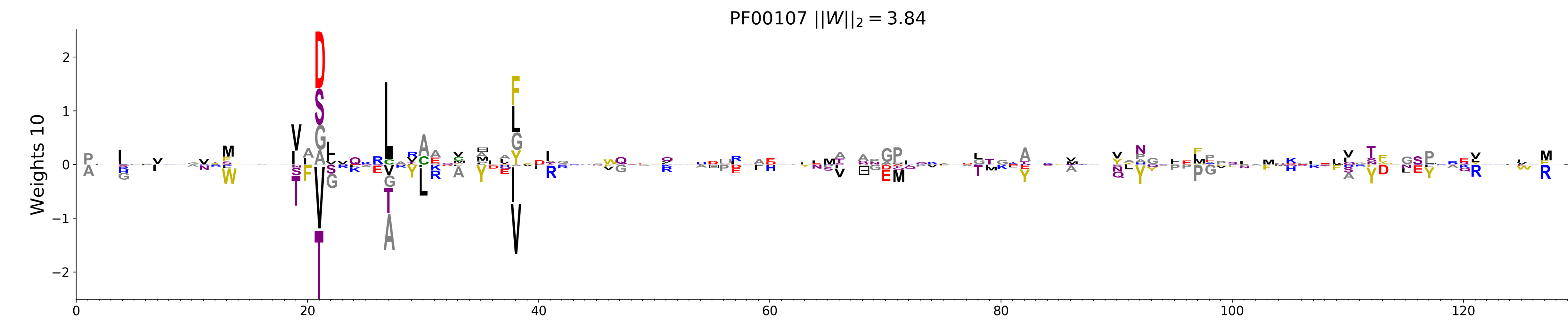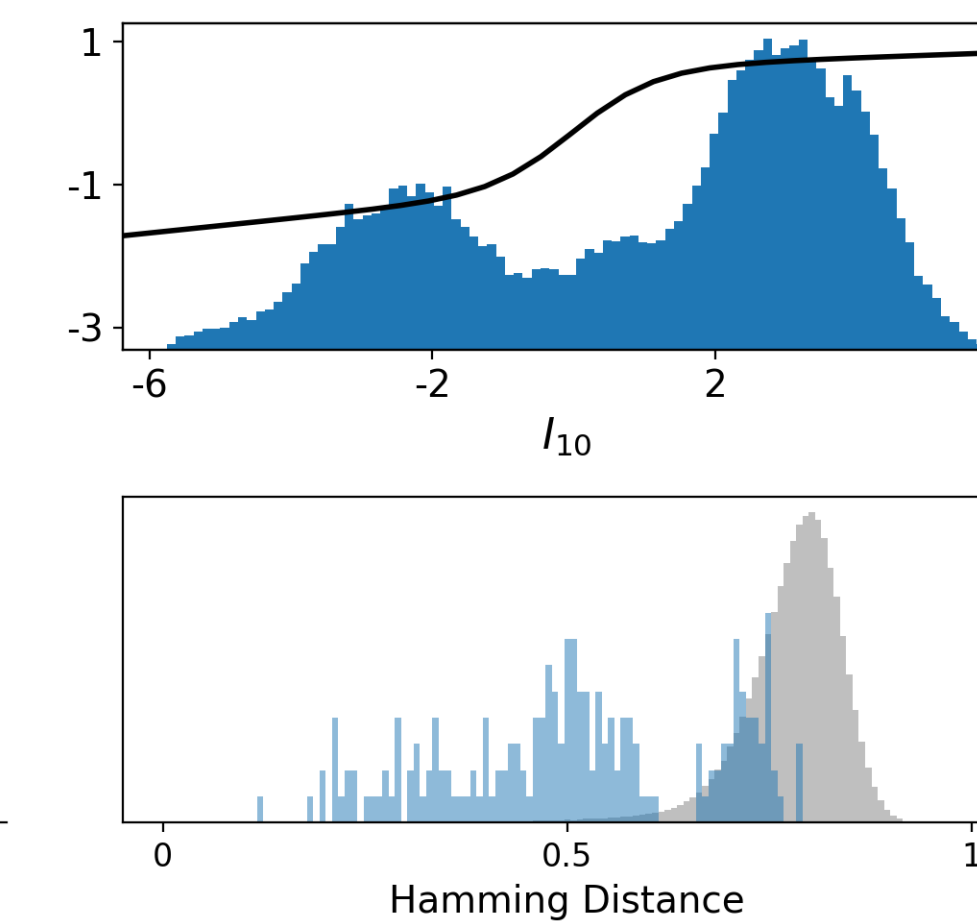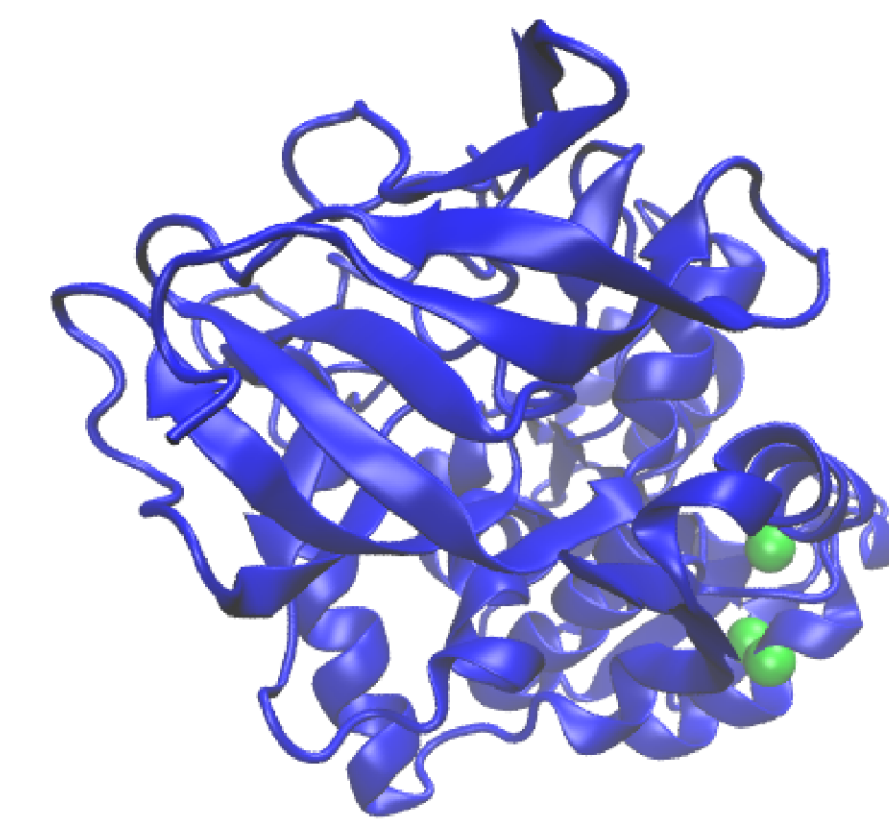

Supplement: Supplementary file 5. [file elife-39397-supp5.zip › Top_features_all/PF00107_top_features.pdf]

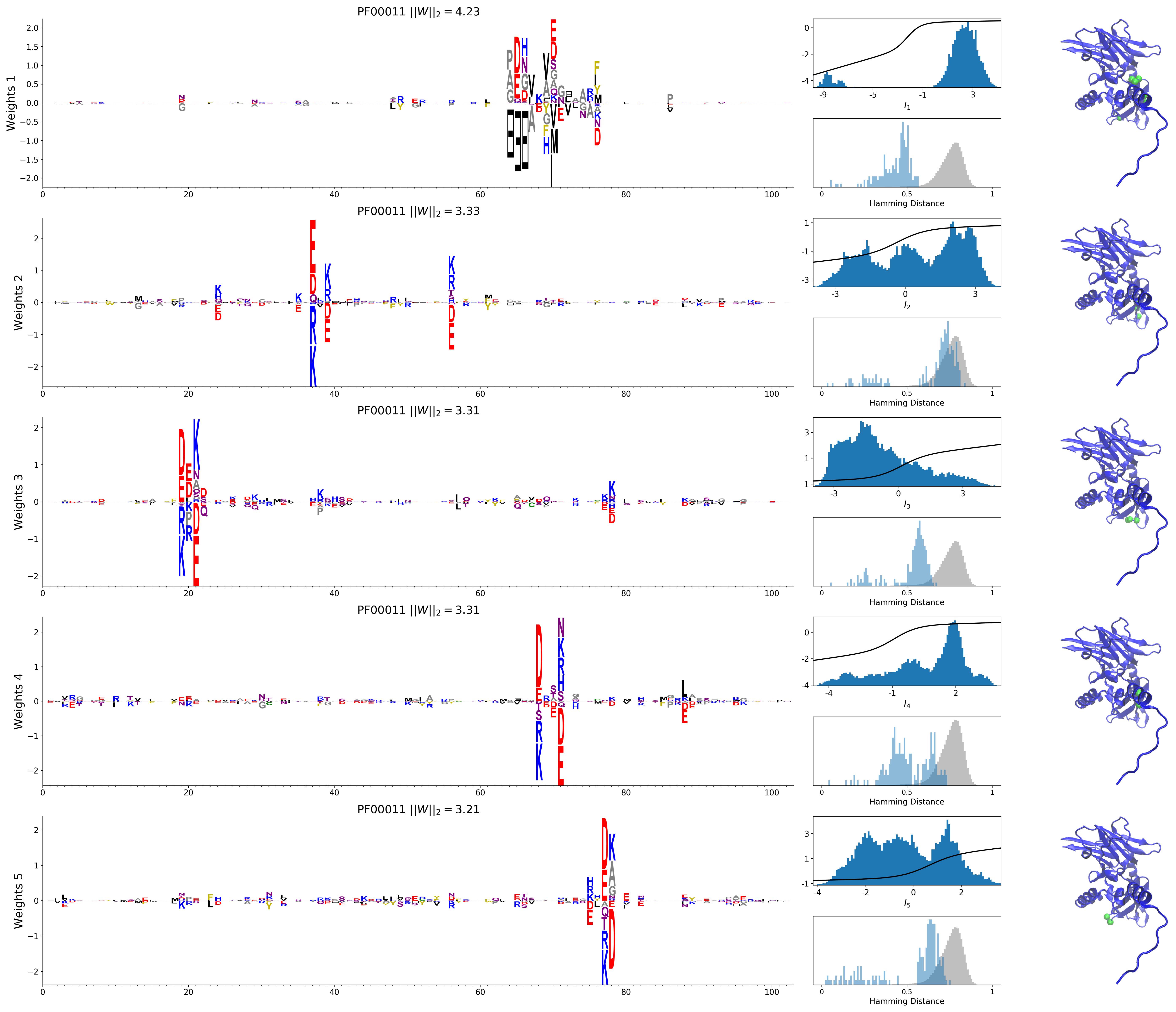

Supplement: Supplementary file 6. [file elife-39397-supp6.zip › Top_Sparse_features_all/PF00011_top_sparse_features.pdf]

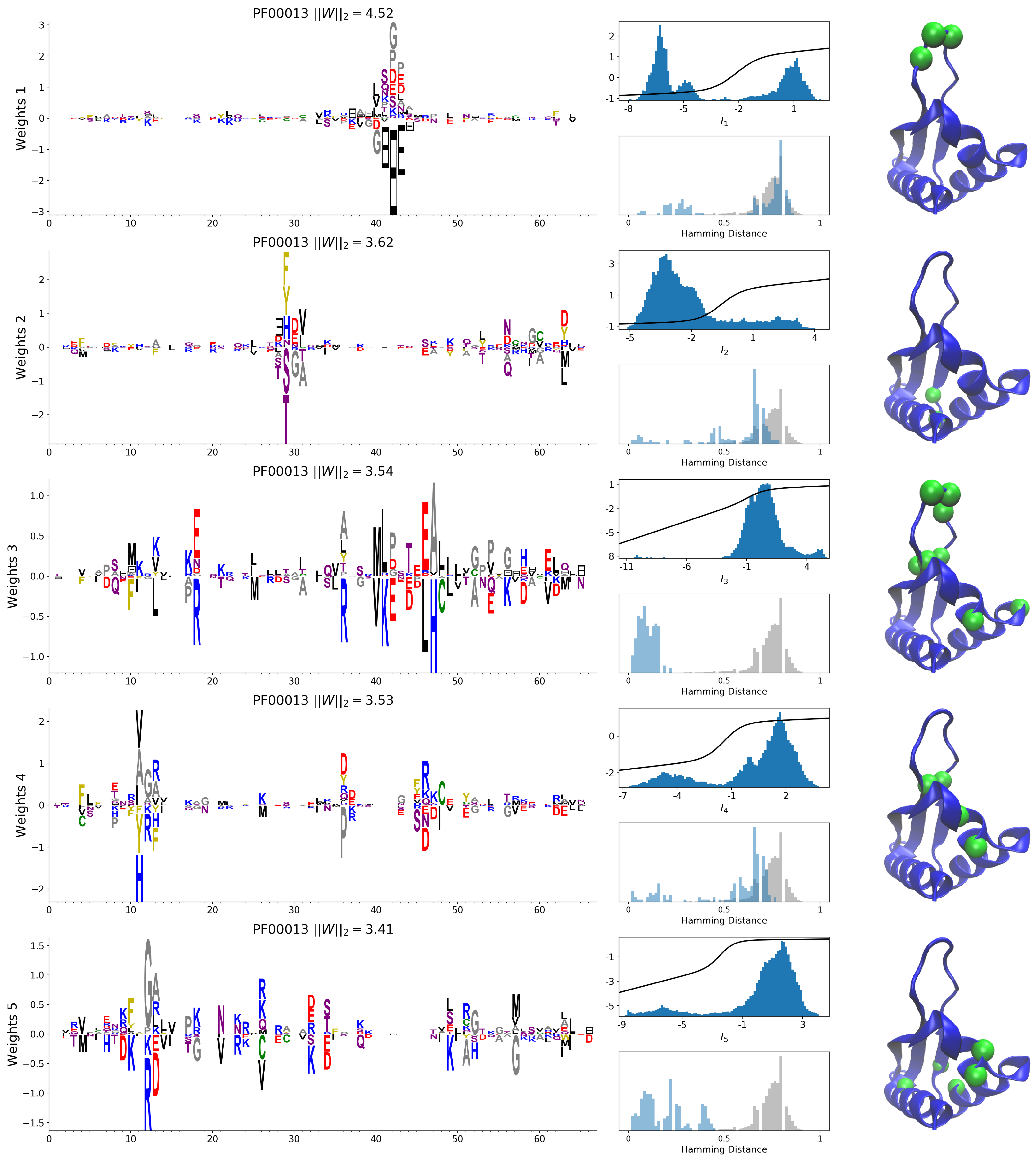

Supplement: Supplementary file 6. [file elife-39397-supp6.zip › Top_Sparse_features_all/PF00013_top_sparse_features.pdf]

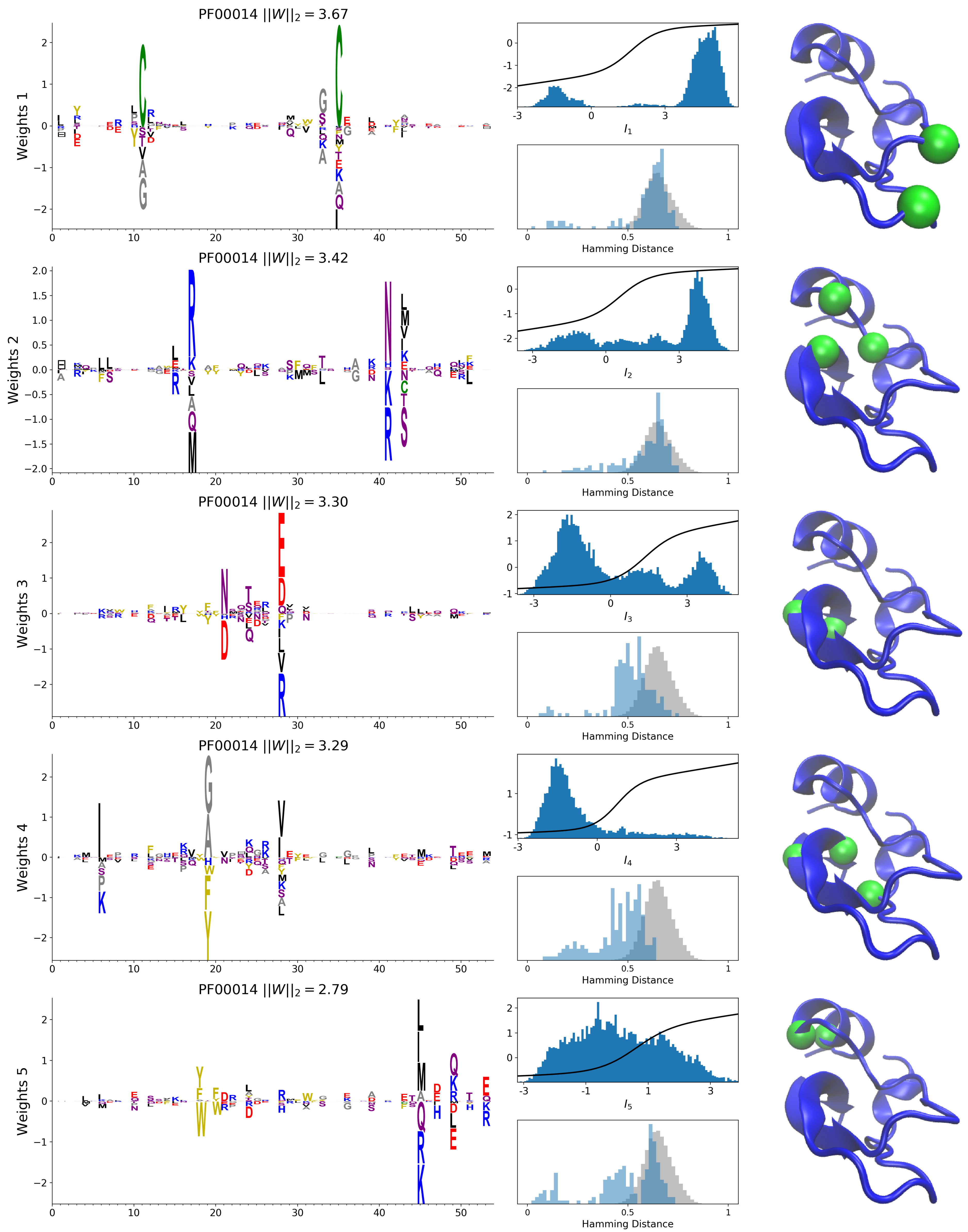

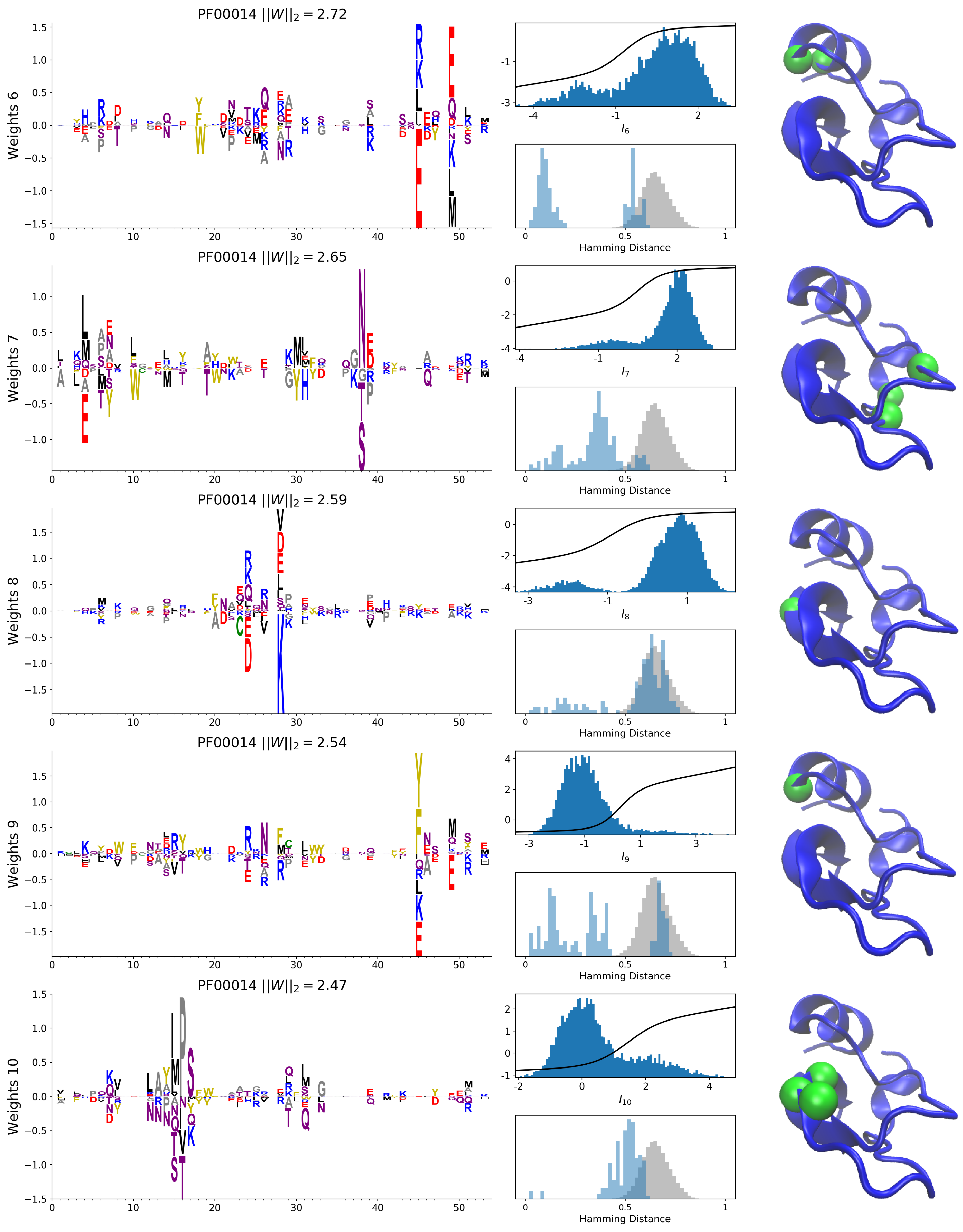

Supplement: Supplementary file 6. [file elife-39397-supp6.zip › Top_Sparse_features_all/PF00014_top_sparse_features.pdf]

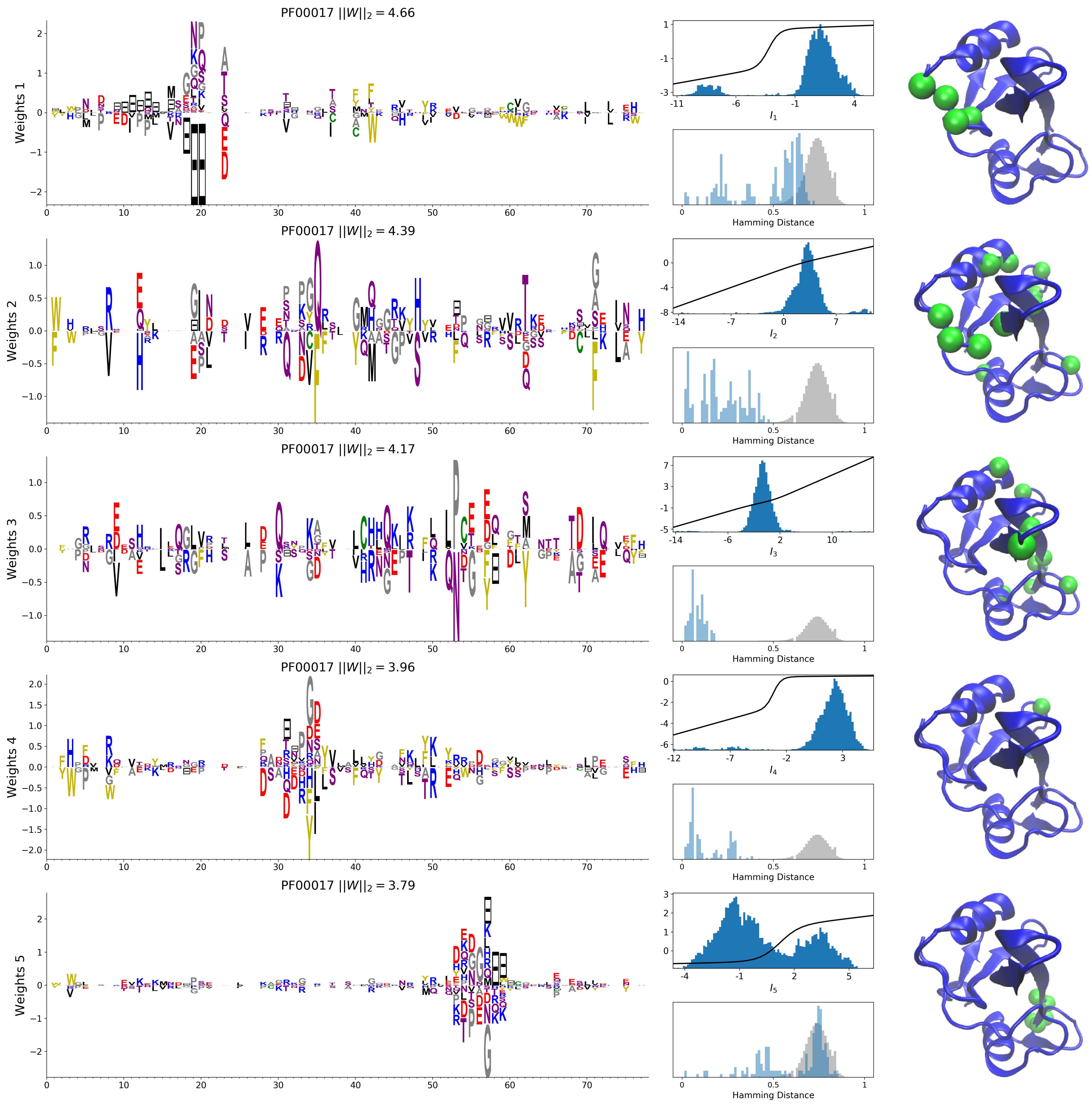

PF00017  $||W||_2 = 3.79$ 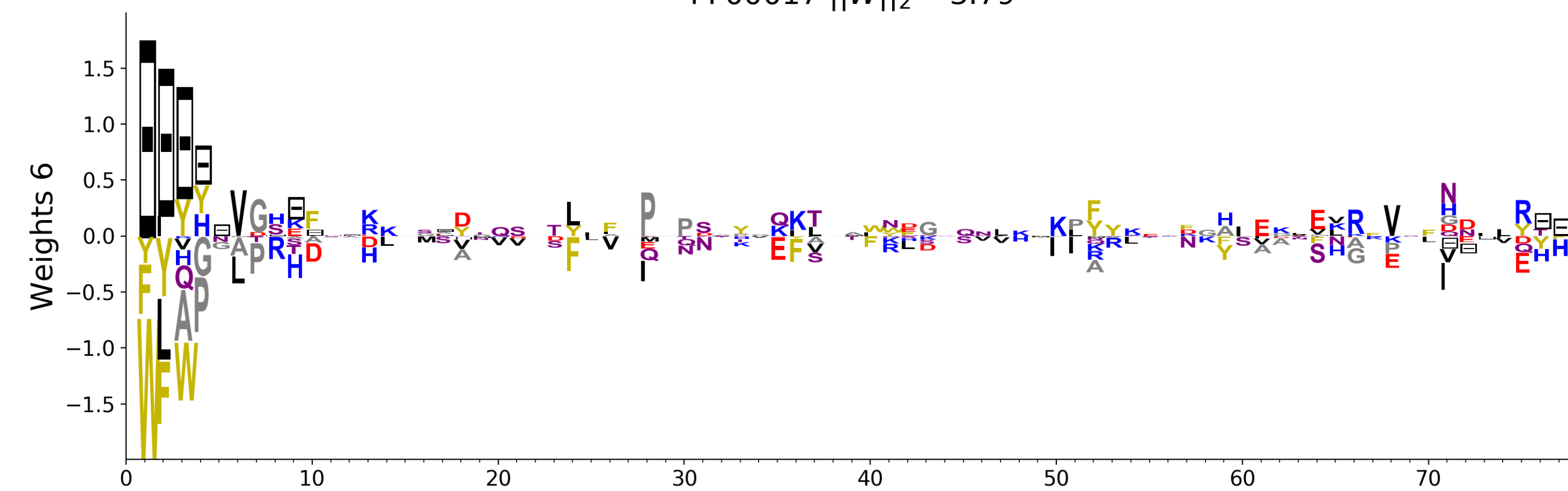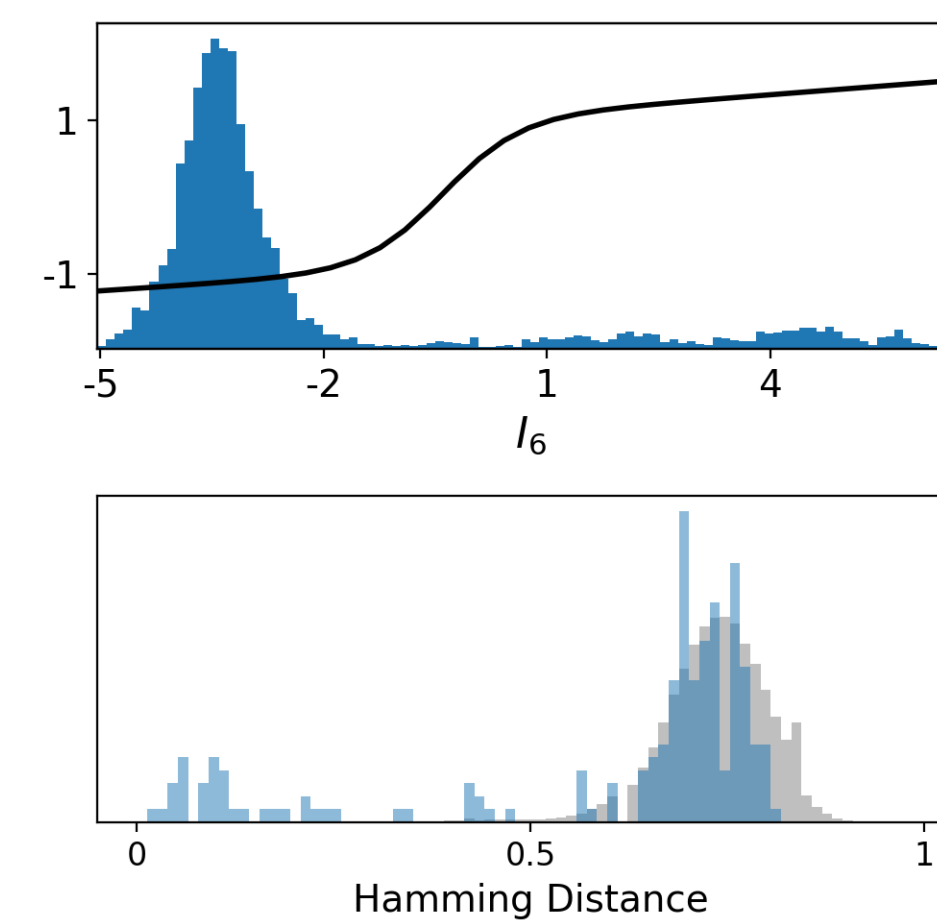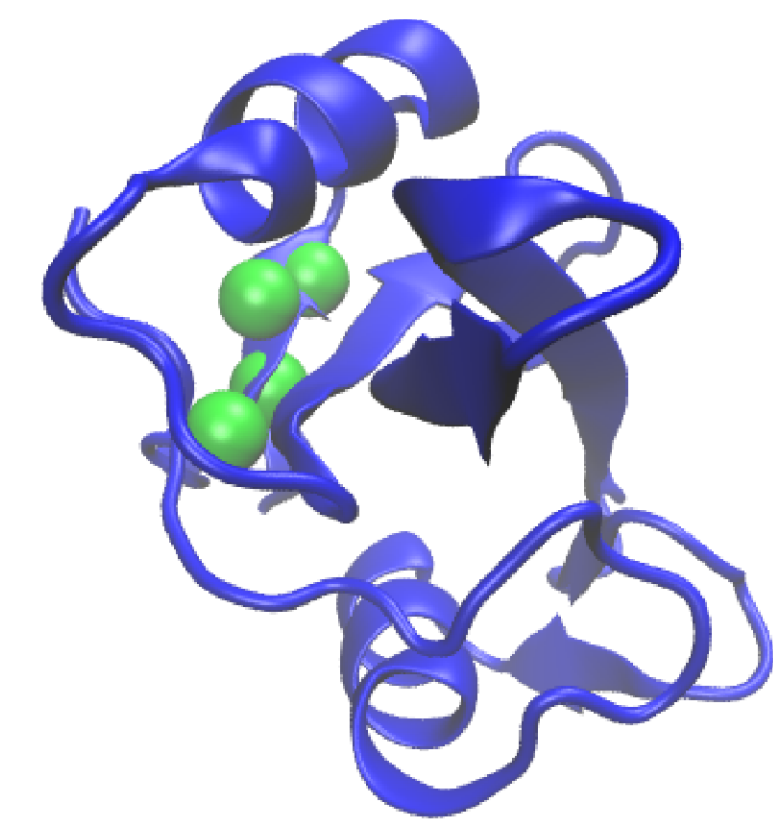PF00017  $||W||_2 = 3.68$ 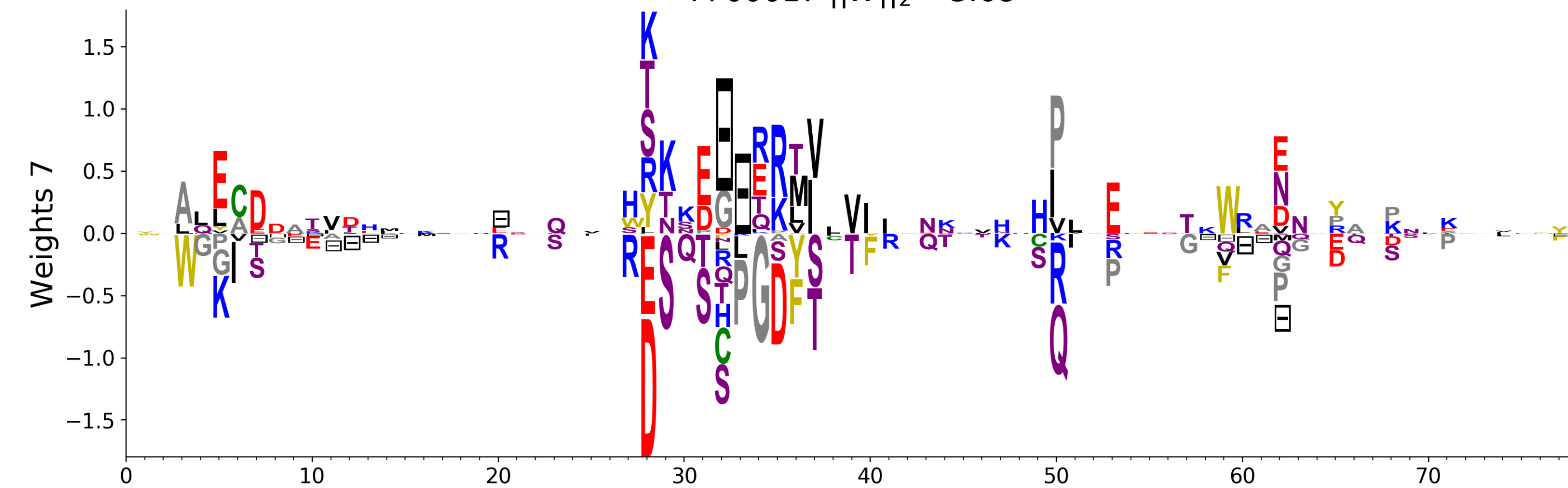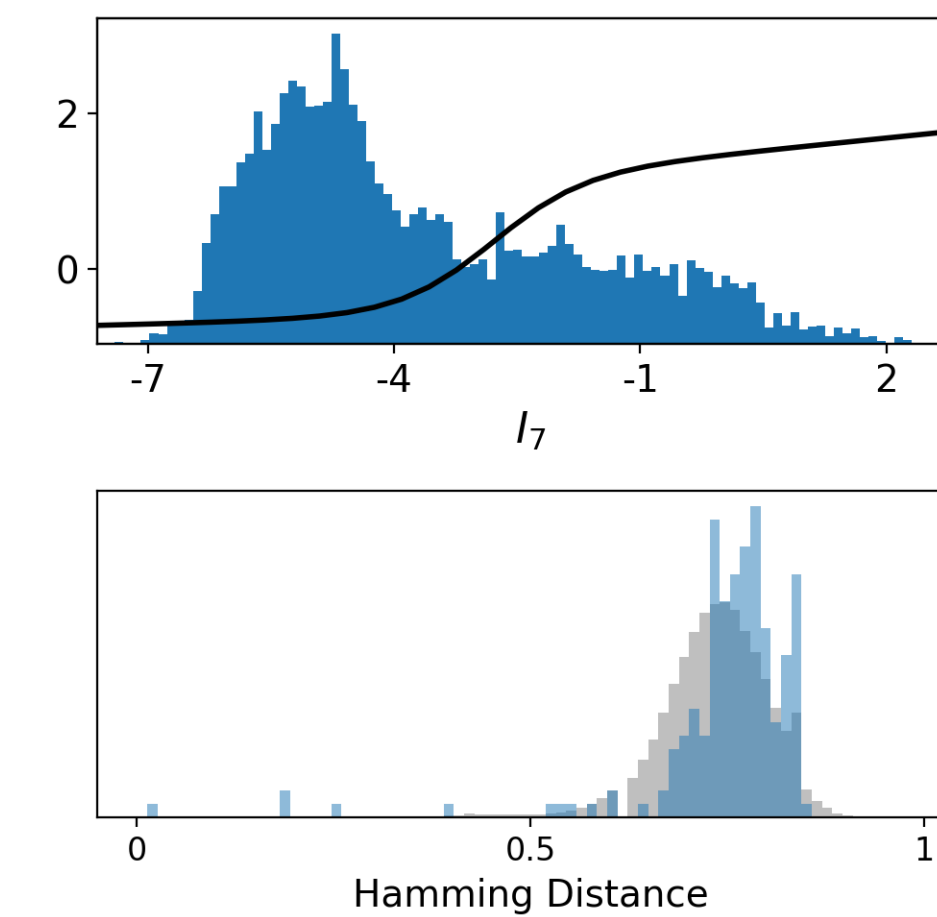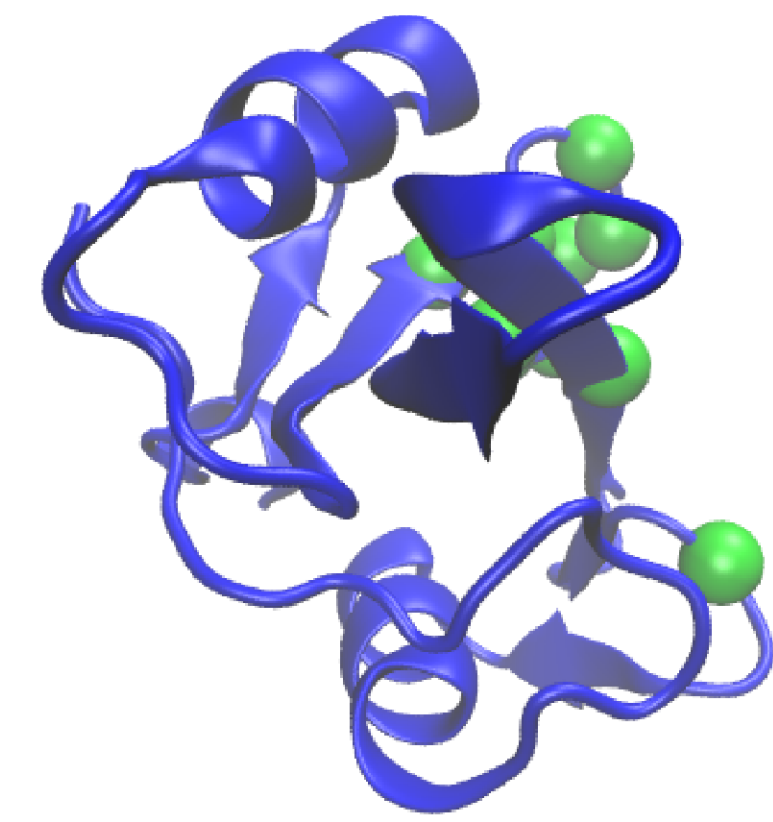PF00017  $||W||_2 = 3.63$ 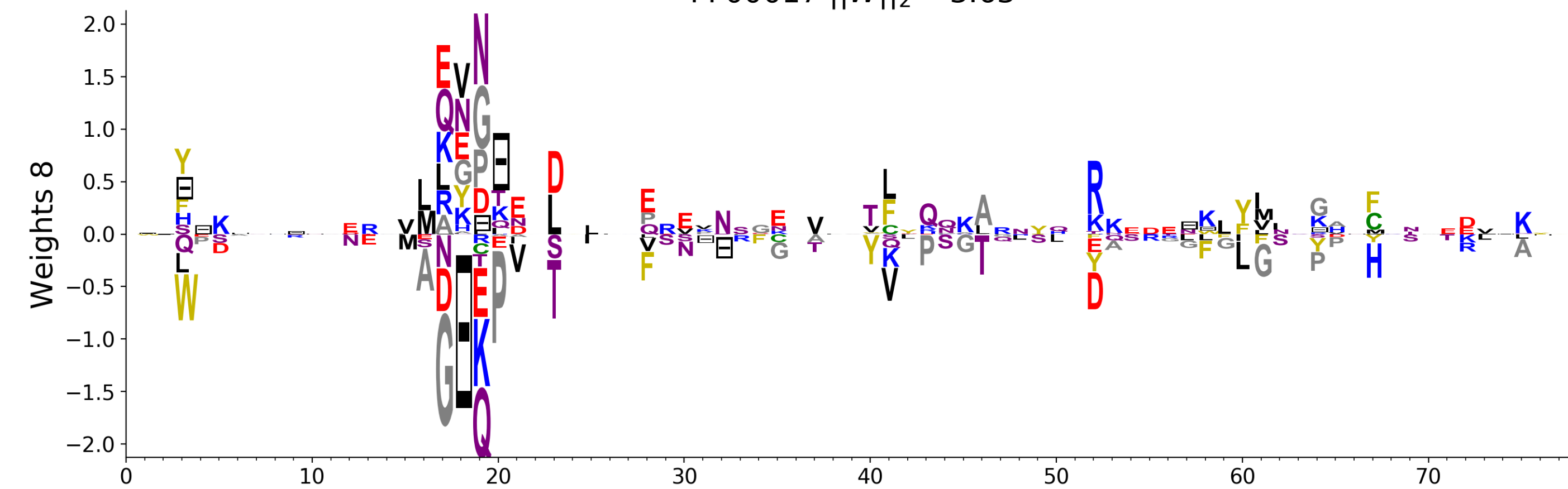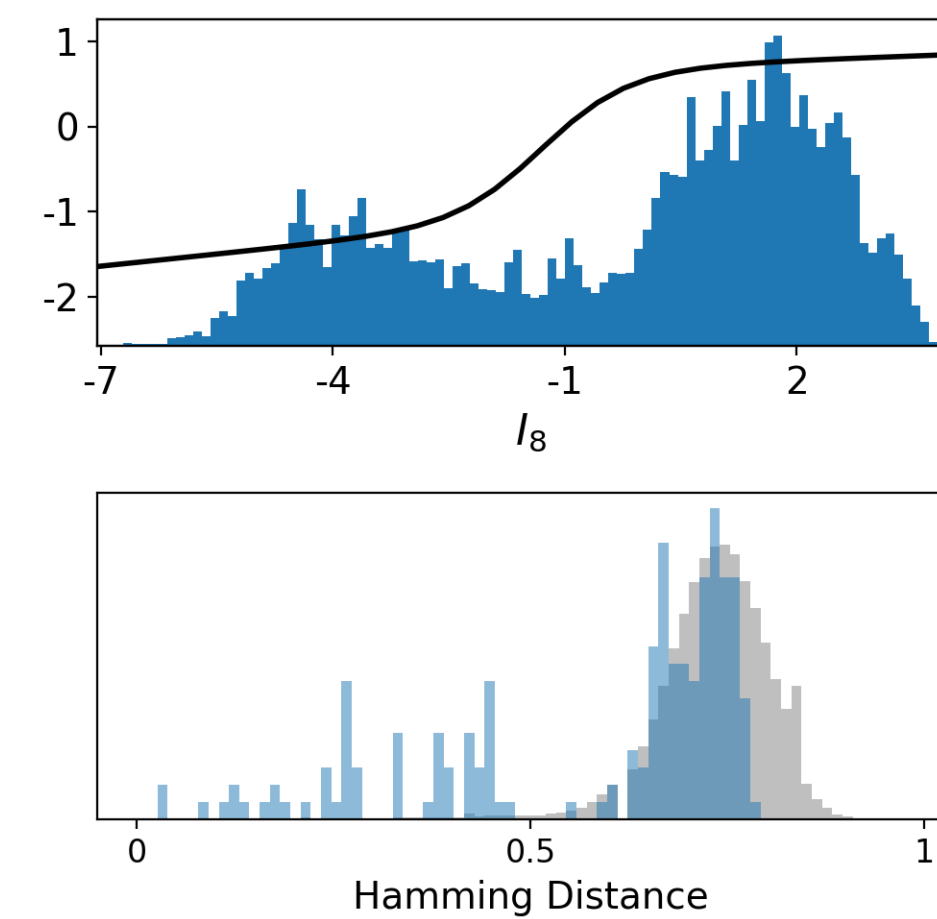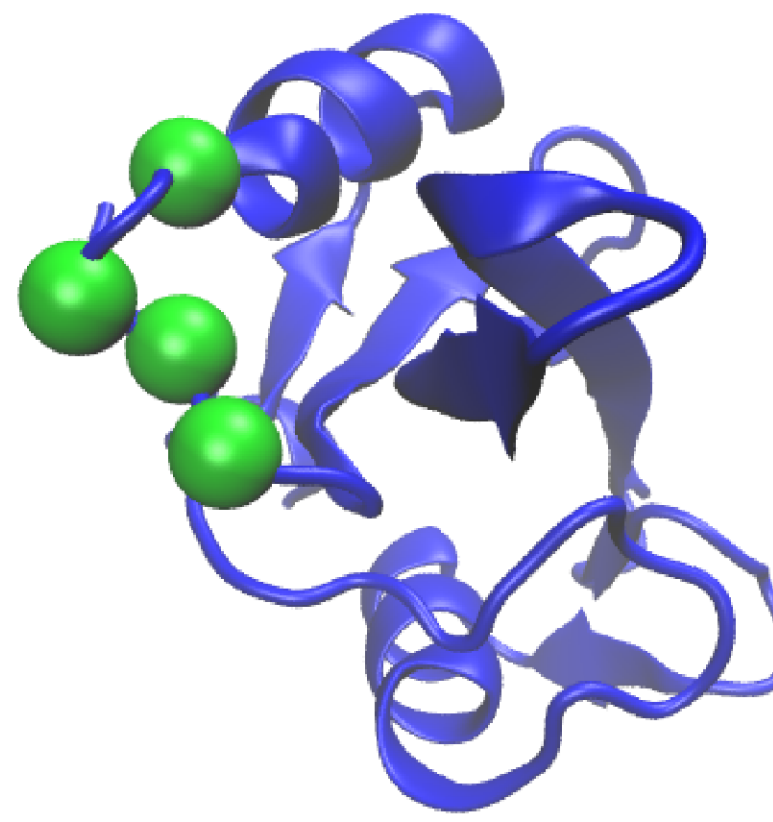PF00017  $||W||_2 = 3.49$ 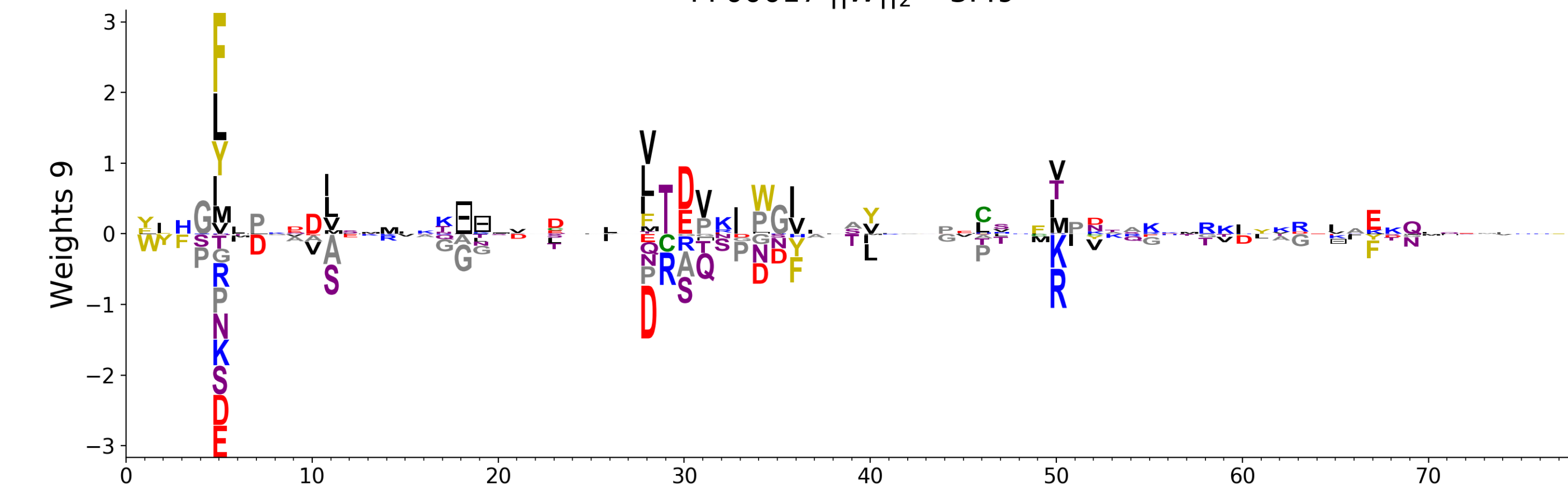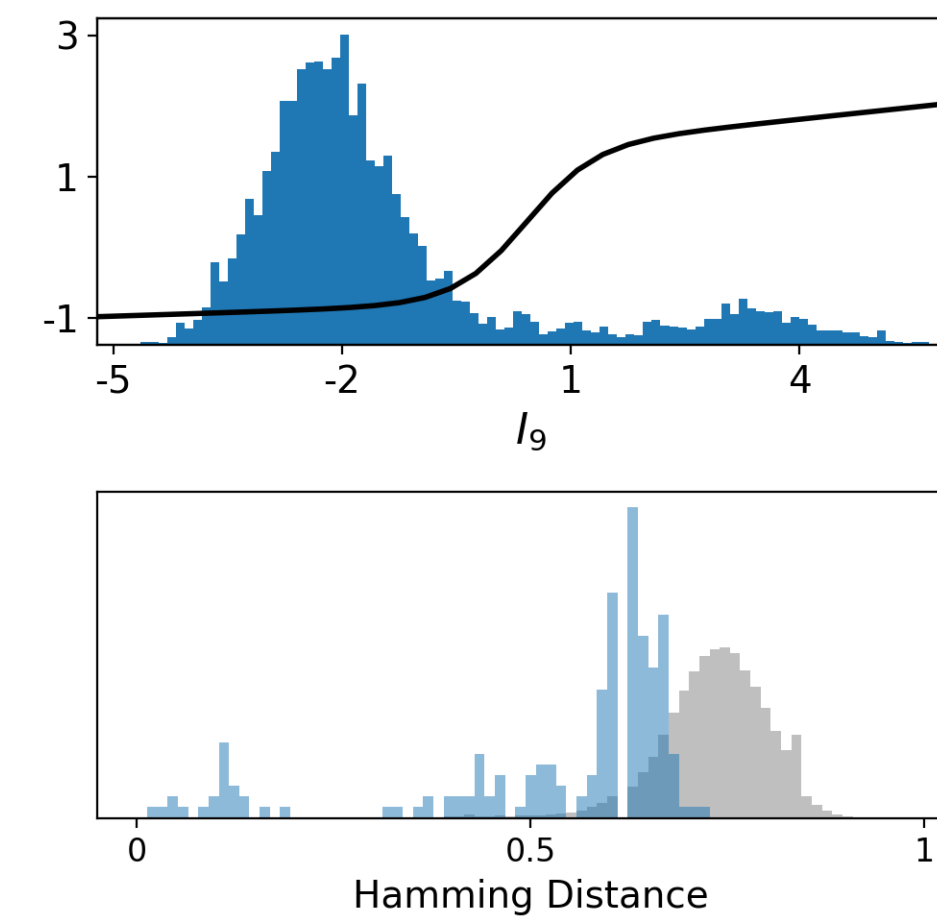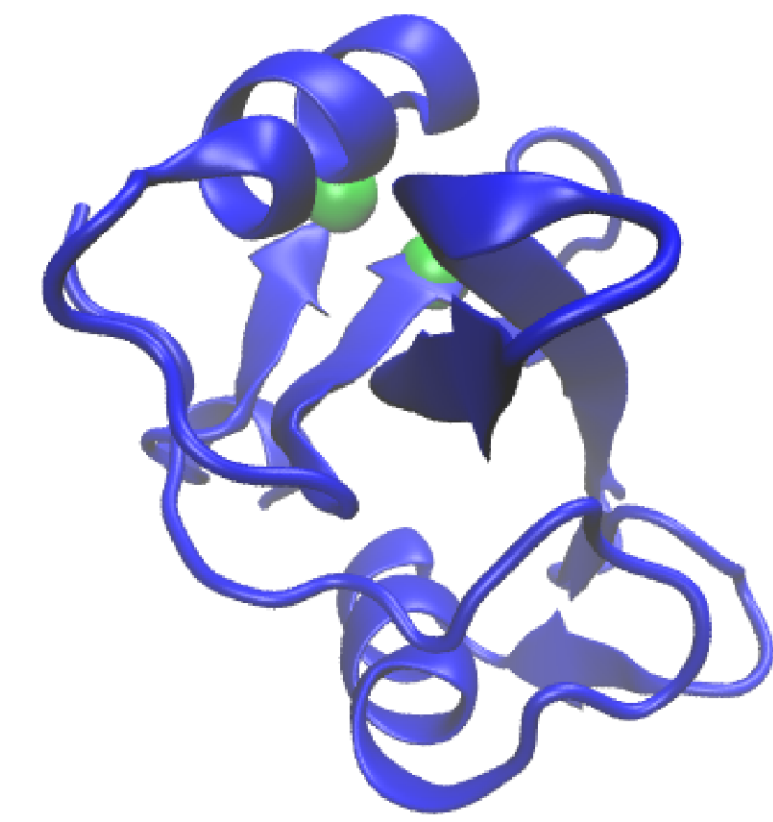PF00017  $||W||_2 = 3.32$ 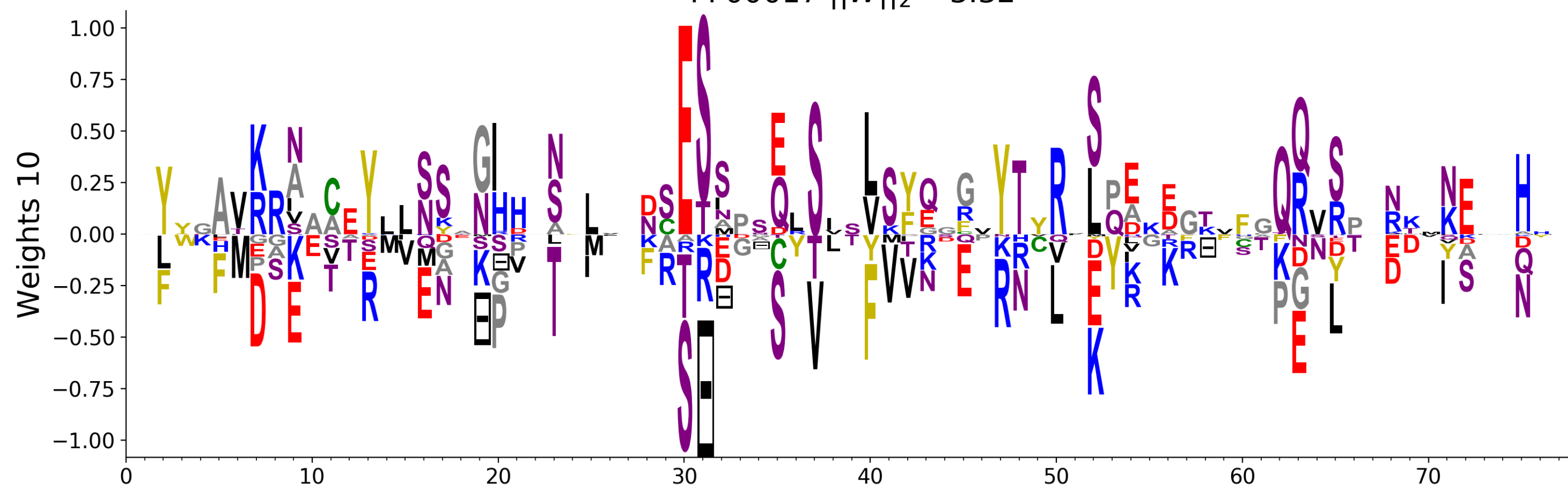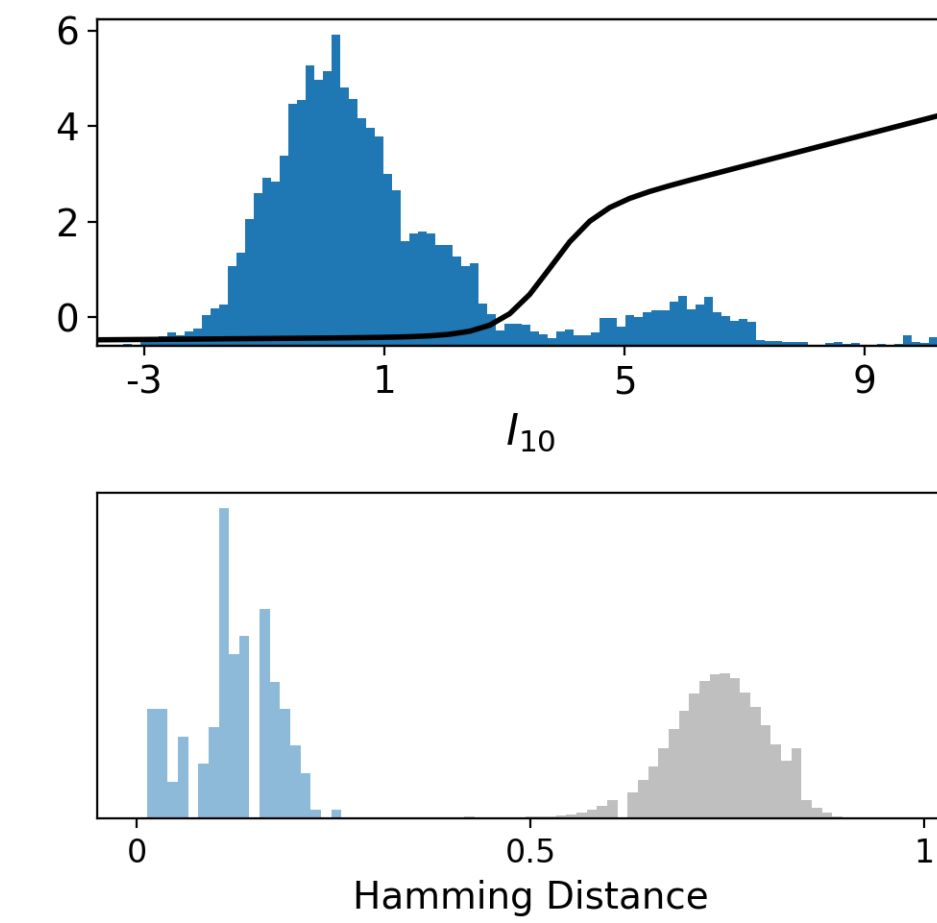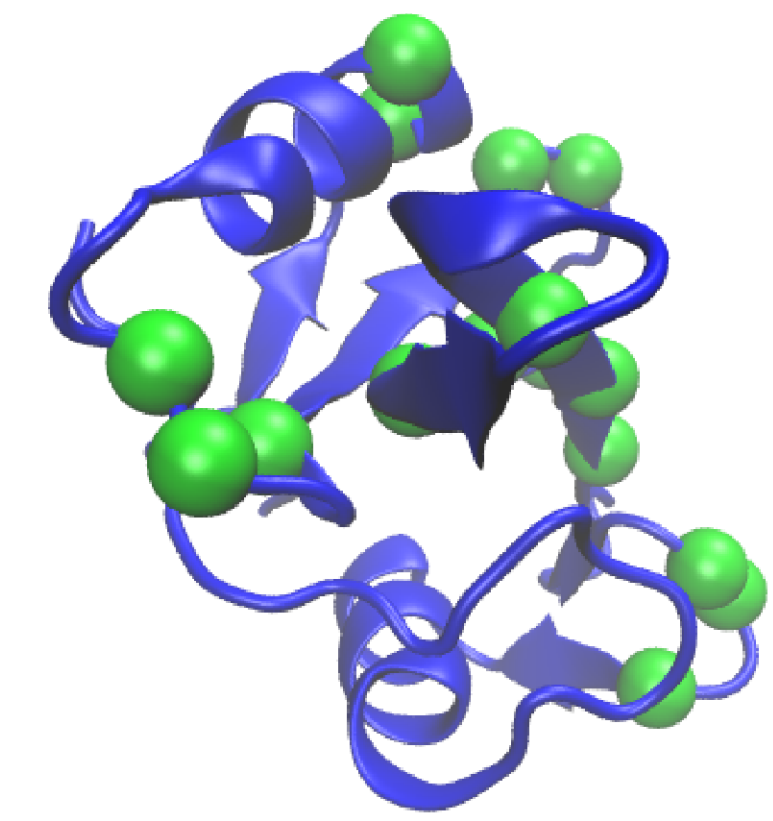

Supplement: Supplementary file 6. [file elife-39397-supp6.zip › Top_Sparse_features_all/PF00017_top_sparse_features.pdf]

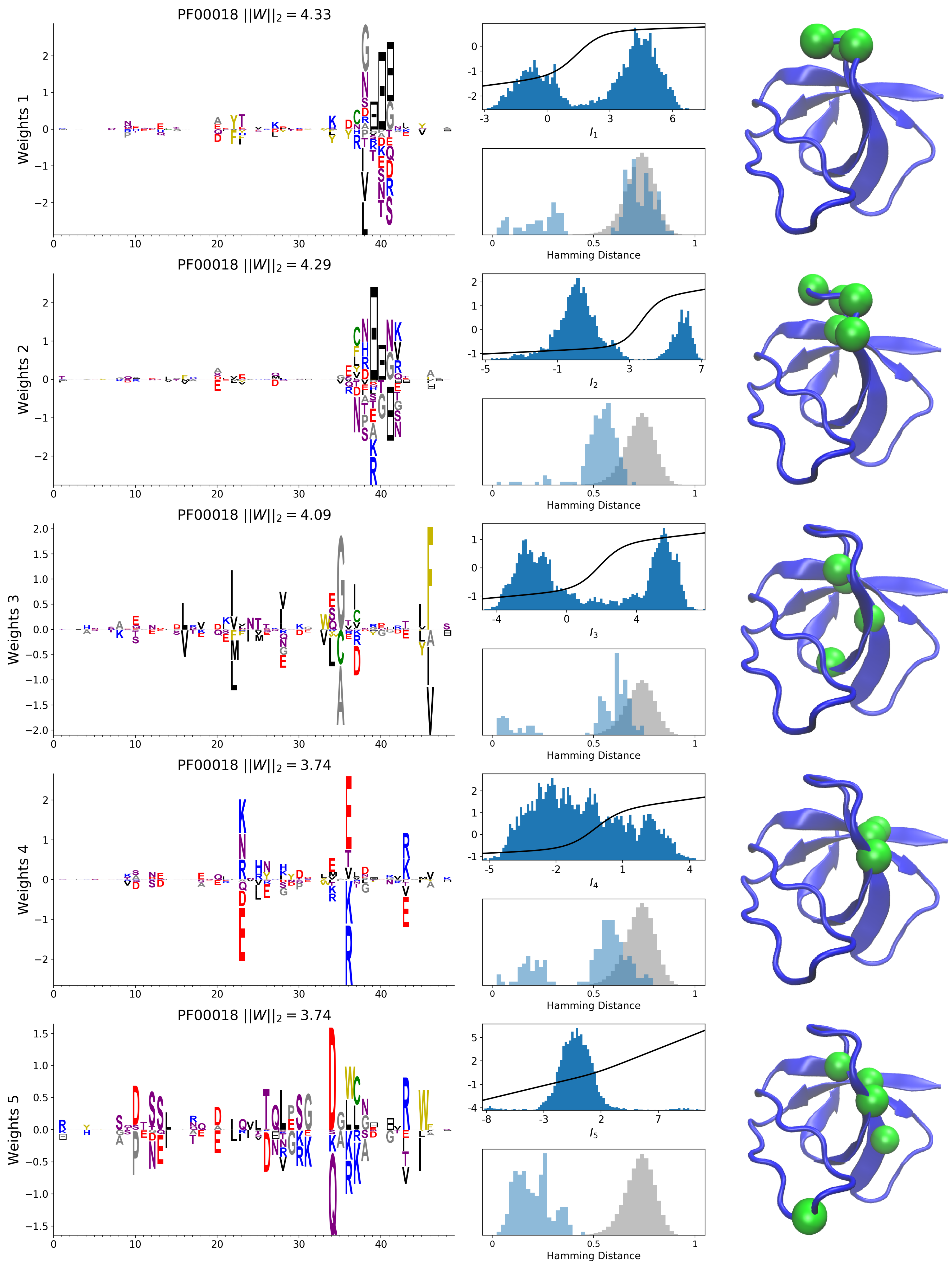

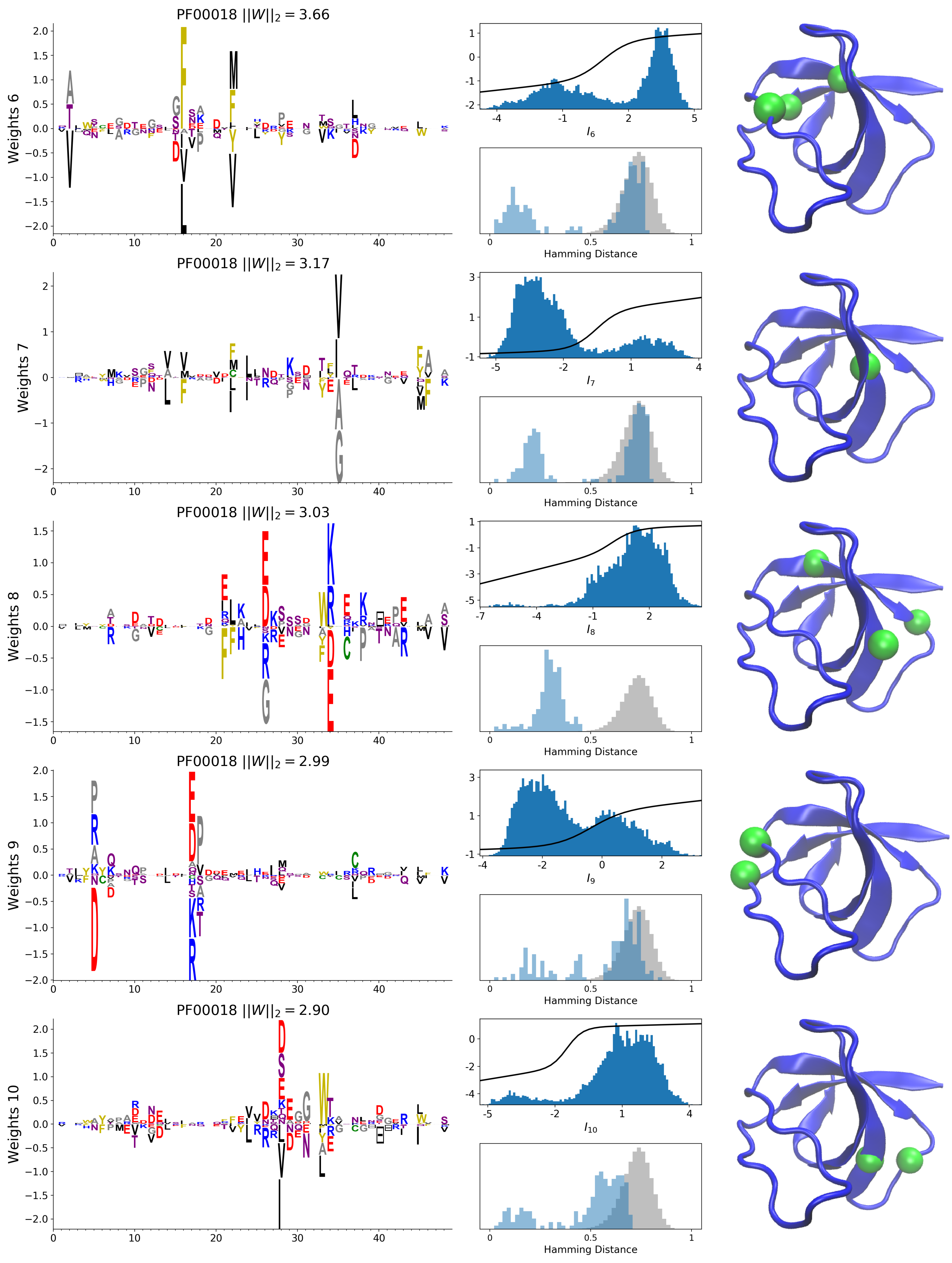

Supplement: Supplementary file 6. [file elife-39397-supp6.zip › Top_Sparse_features_all/PF00018_top_sparse_features.pdf]

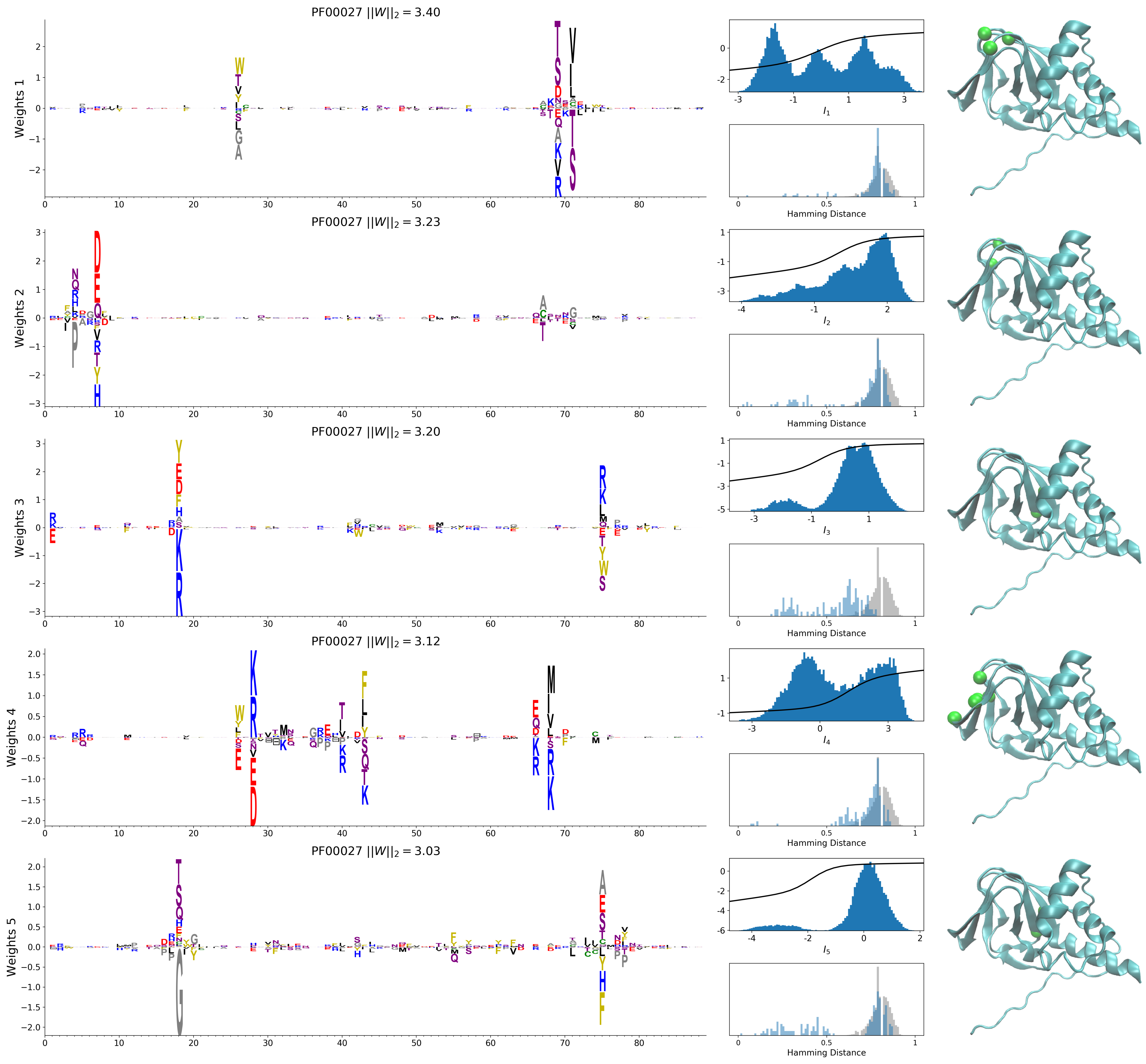

Supplement: Supplementary file 6. [file elife-39397-supp6.zip › Top_Sparse_features_all/PF00027_top_sparse_features.pdf]

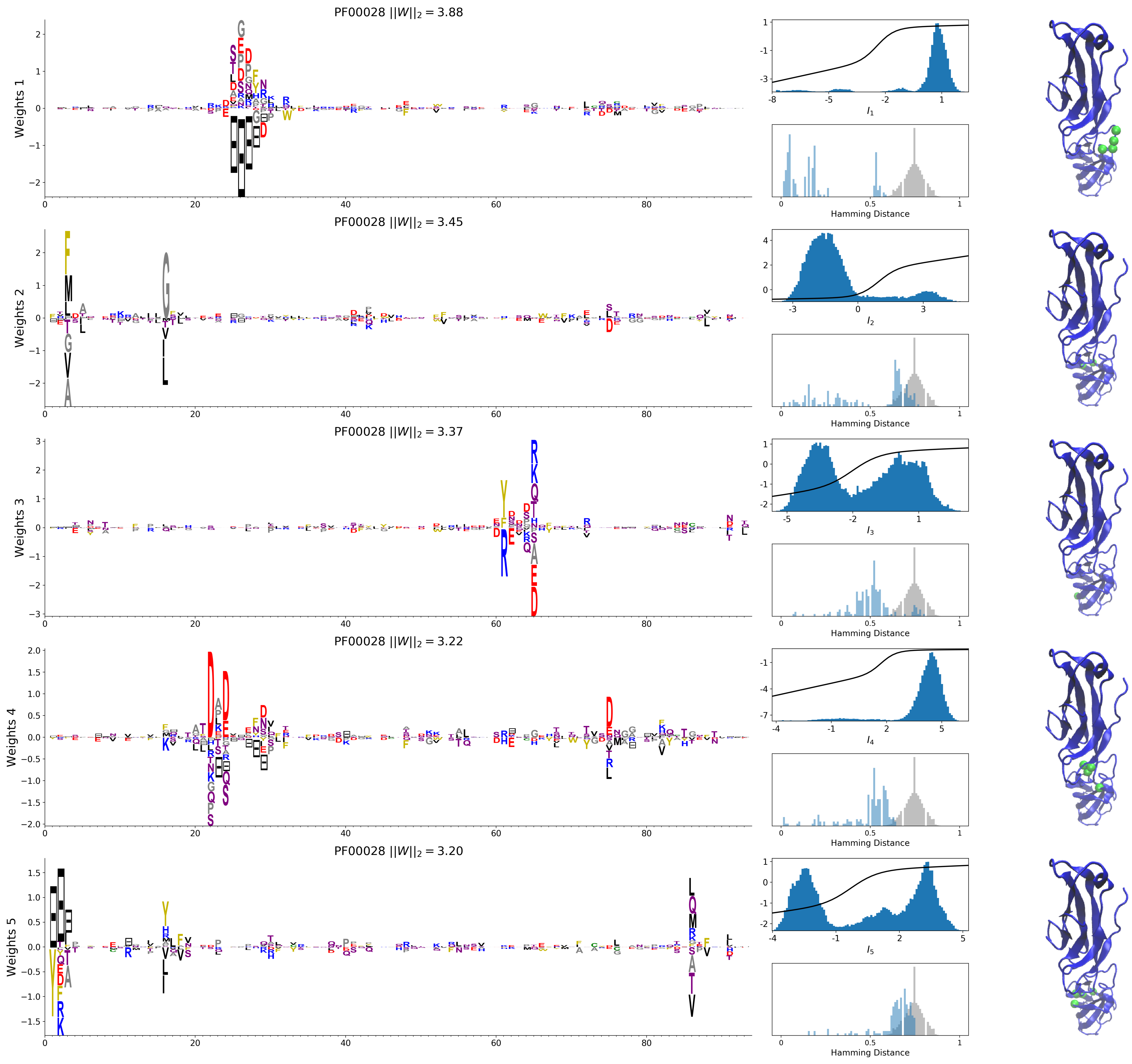

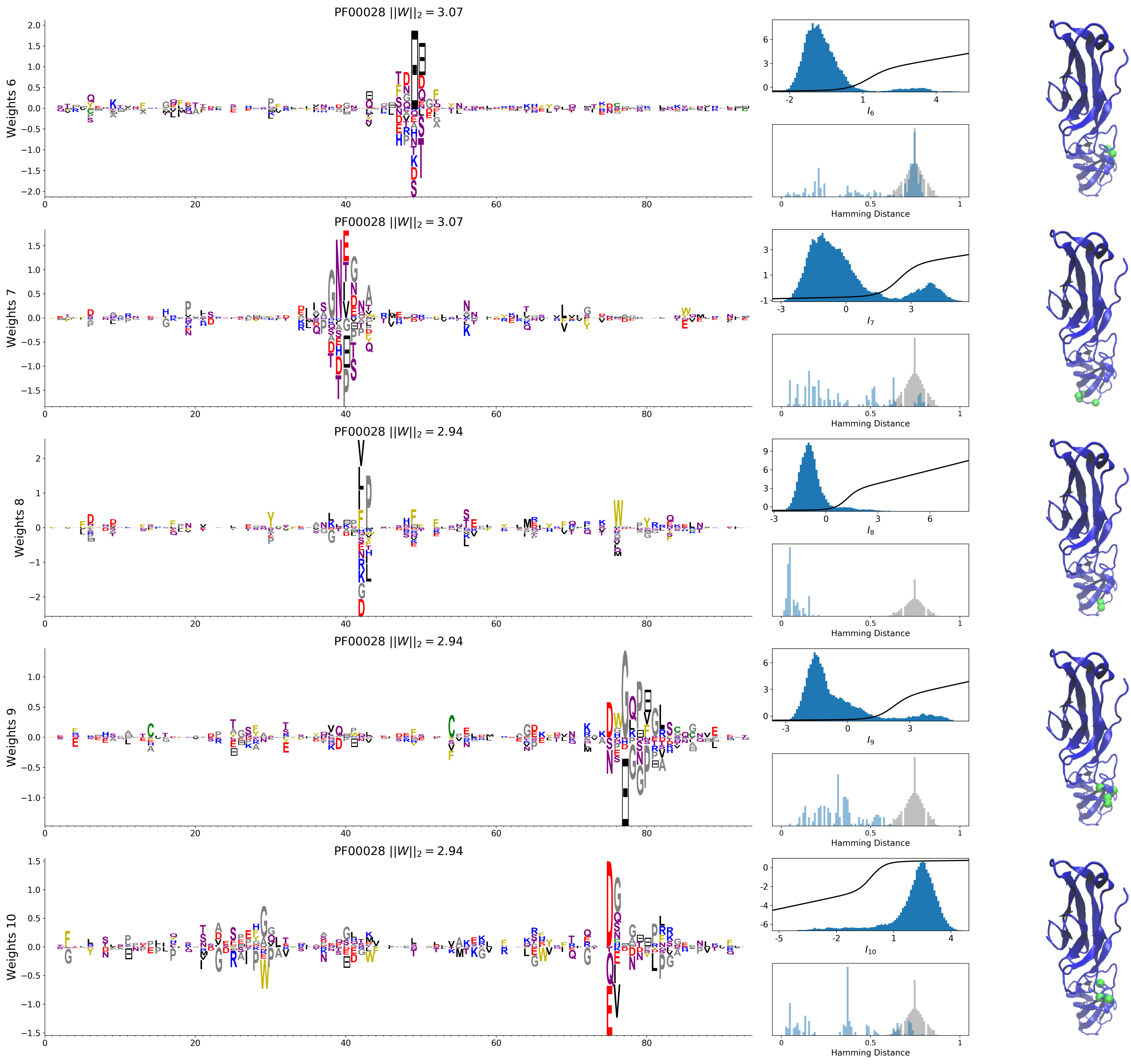

Supplement: Supplementary file 6. [file elife-39397-supp6.zip › Top_Sparse_features_all/PF00028_top_sparse_features.pdf]

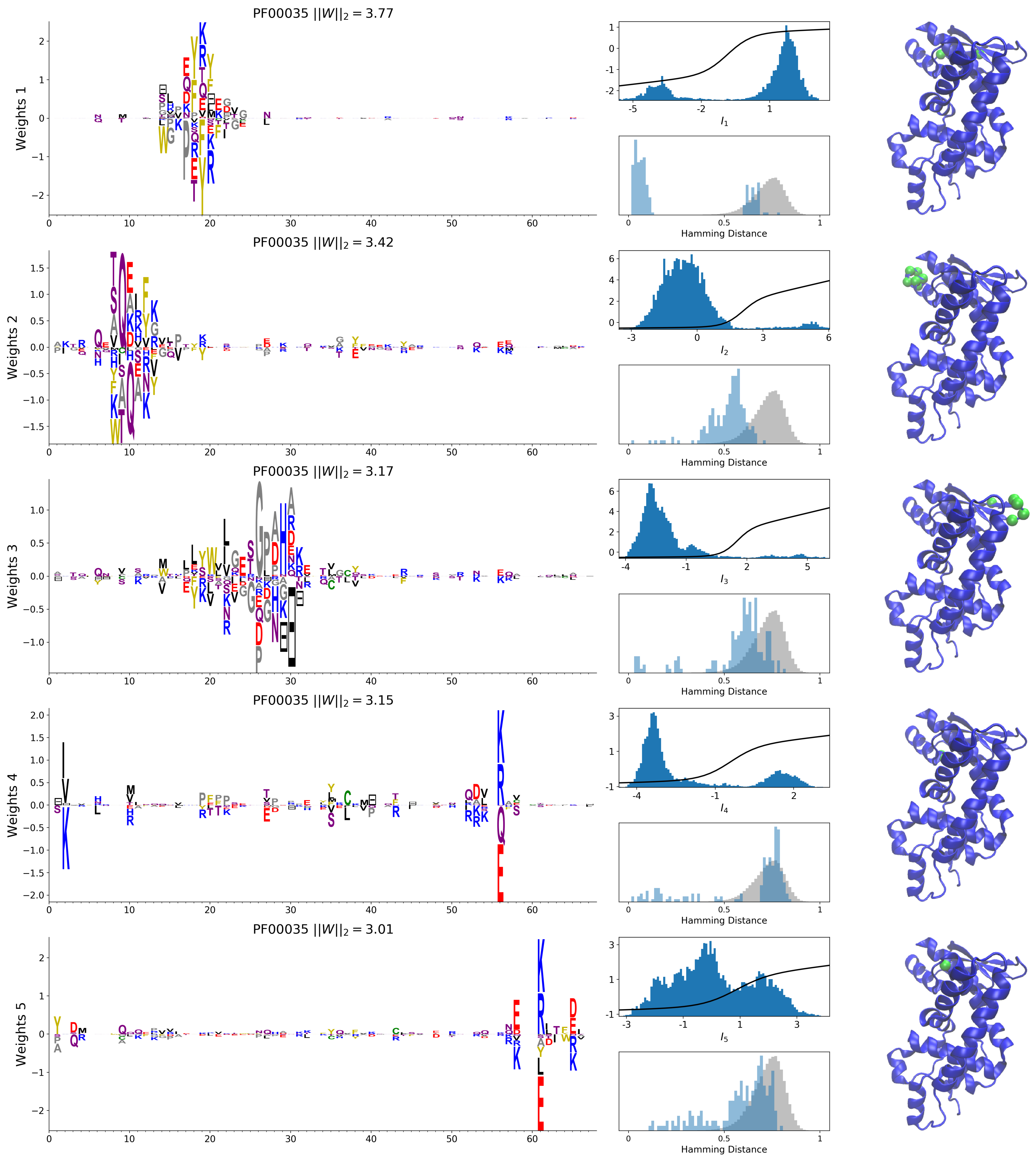

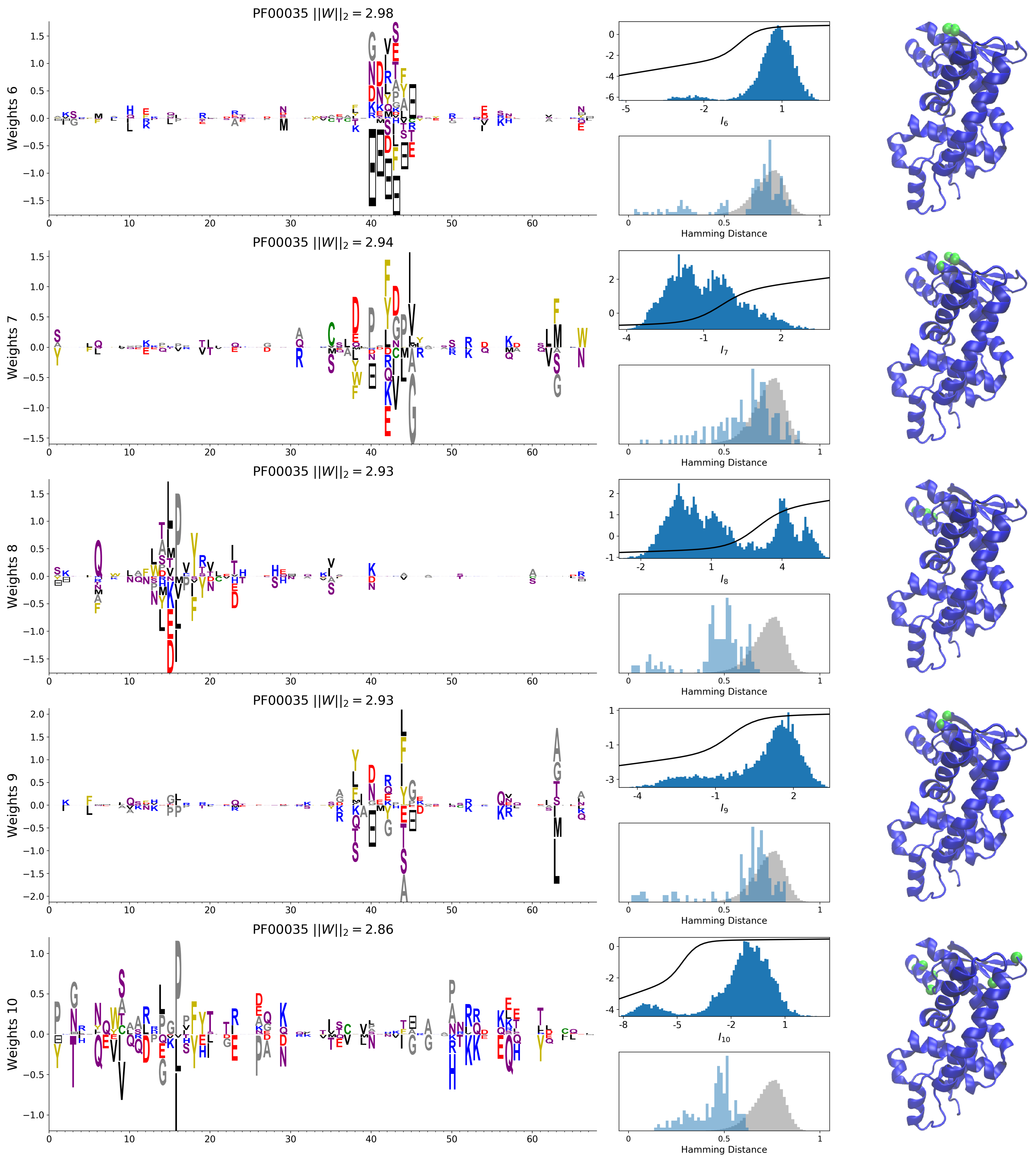

Supplement: Supplementary file 6. [file elife-39397-supp6.zip › Top_Sparse_features_all/PF00035_top_sparse_features.pdf]

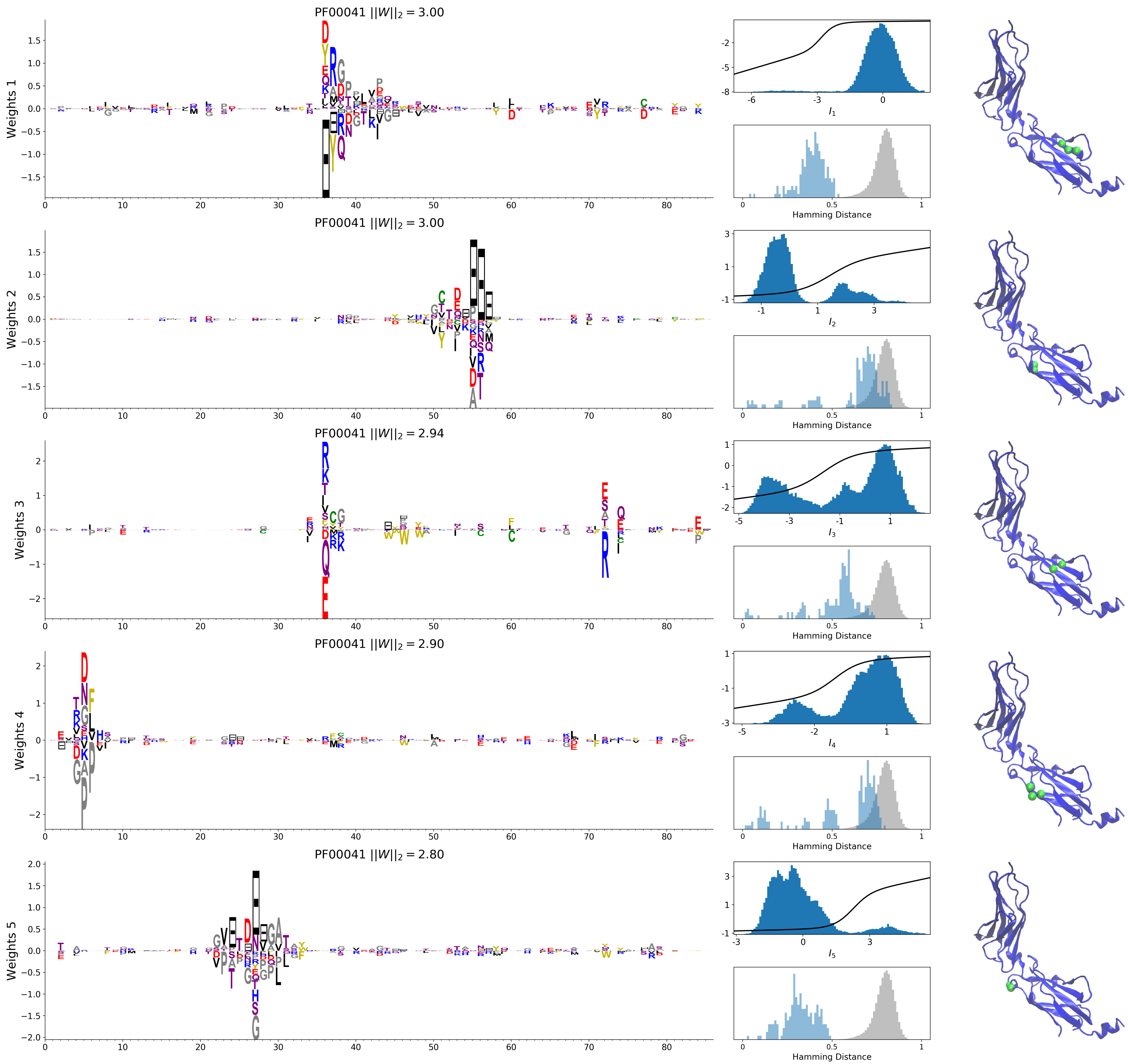

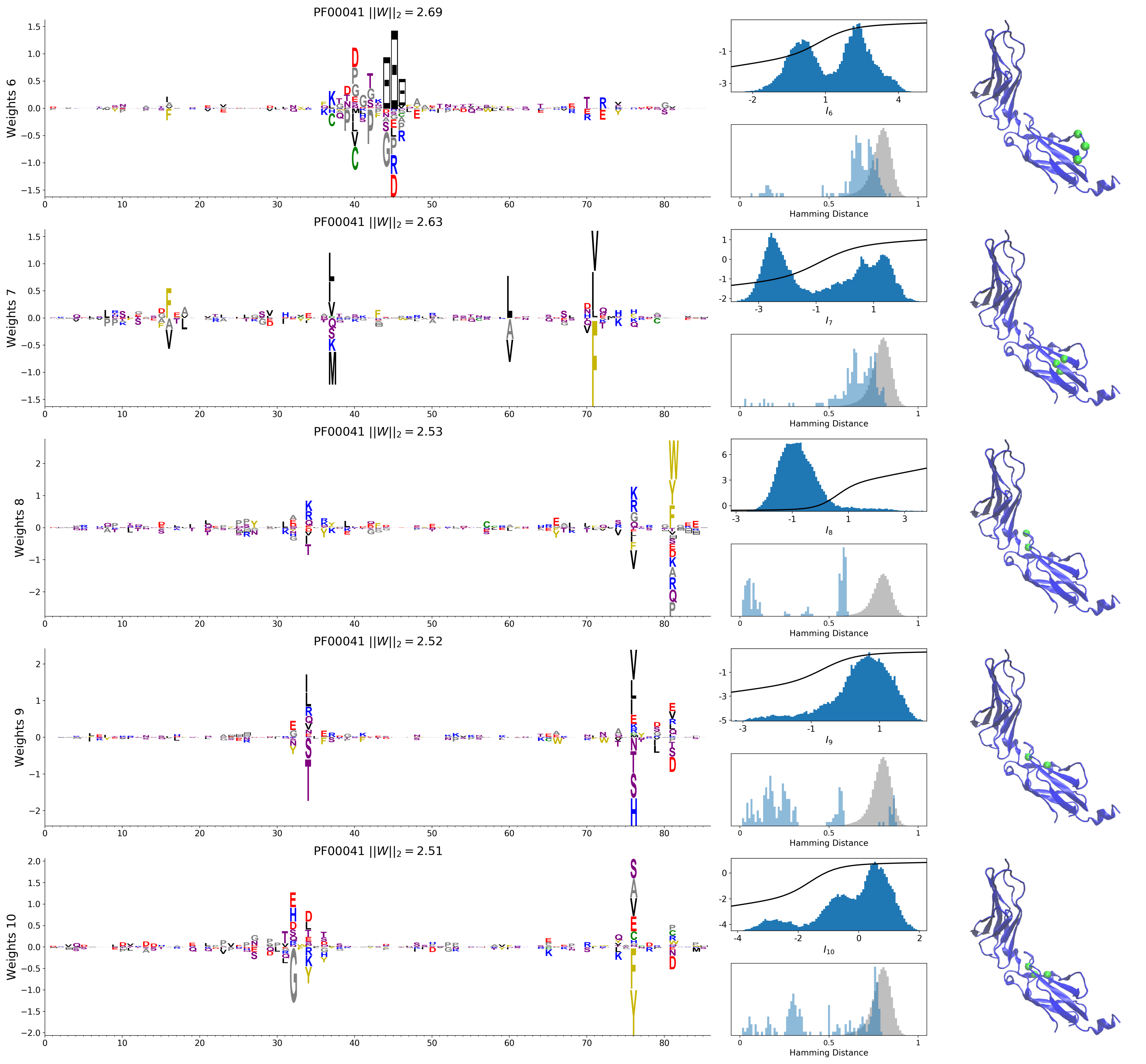

Supplement: Supplementary file 6. [file elife-39397-supp6.zip › Top_Sparse_features_all/PF00041_top_sparse_features.pdf]

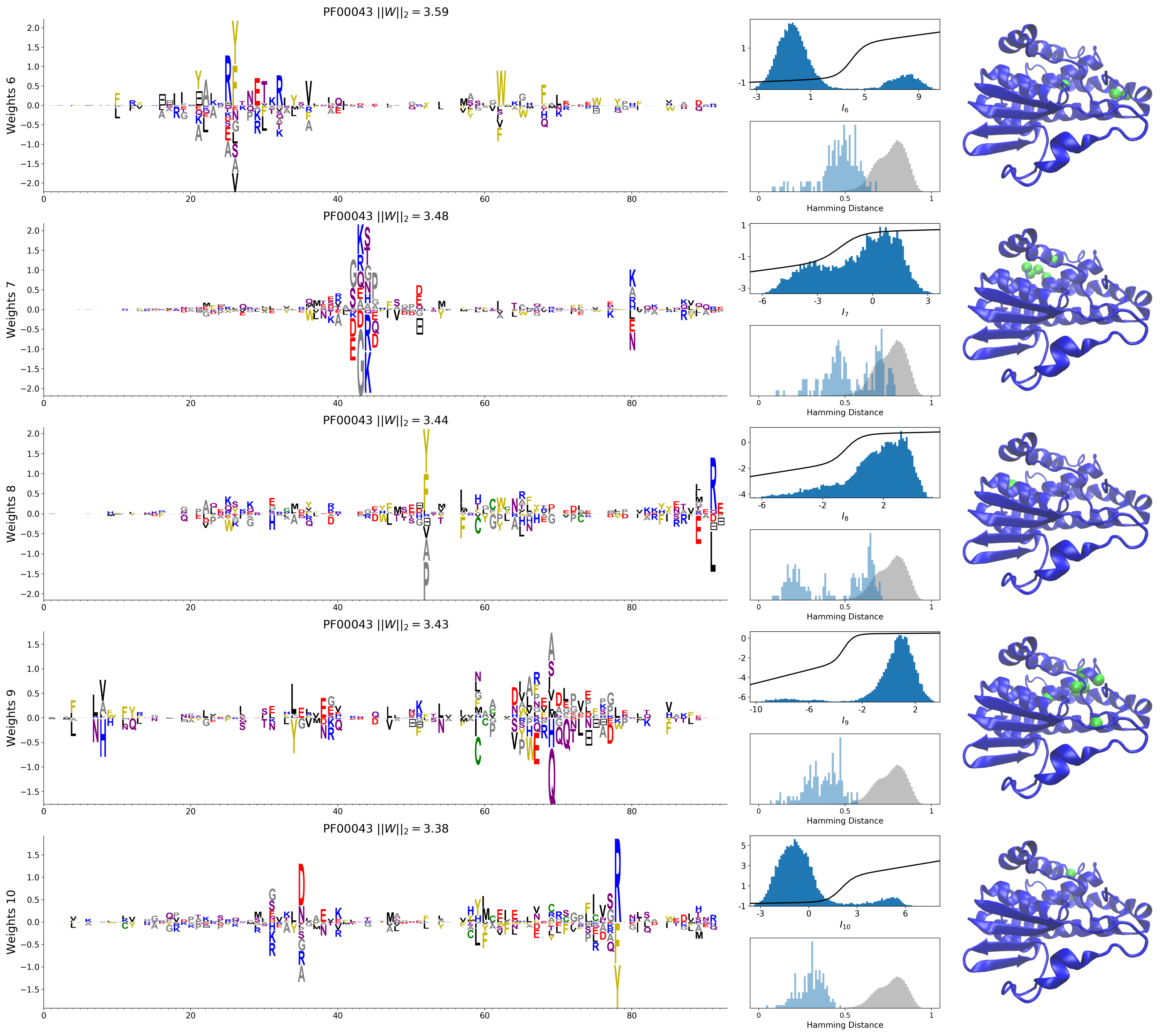

Supplement: Supplementary file 6. [file elife-39397-supp6.zip › Top_Sparse_features_all/PF00043_top_sparse_features.pdf]

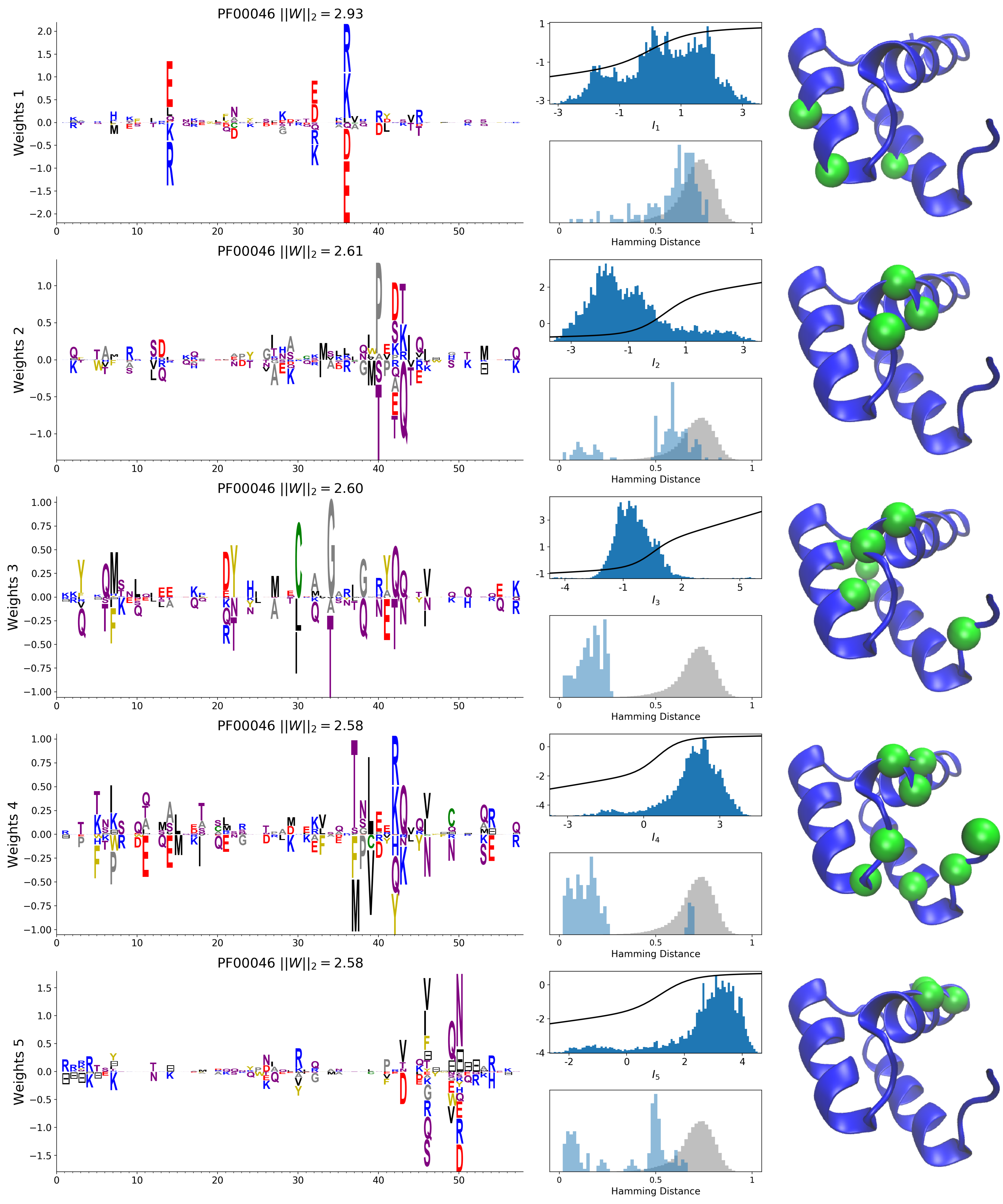

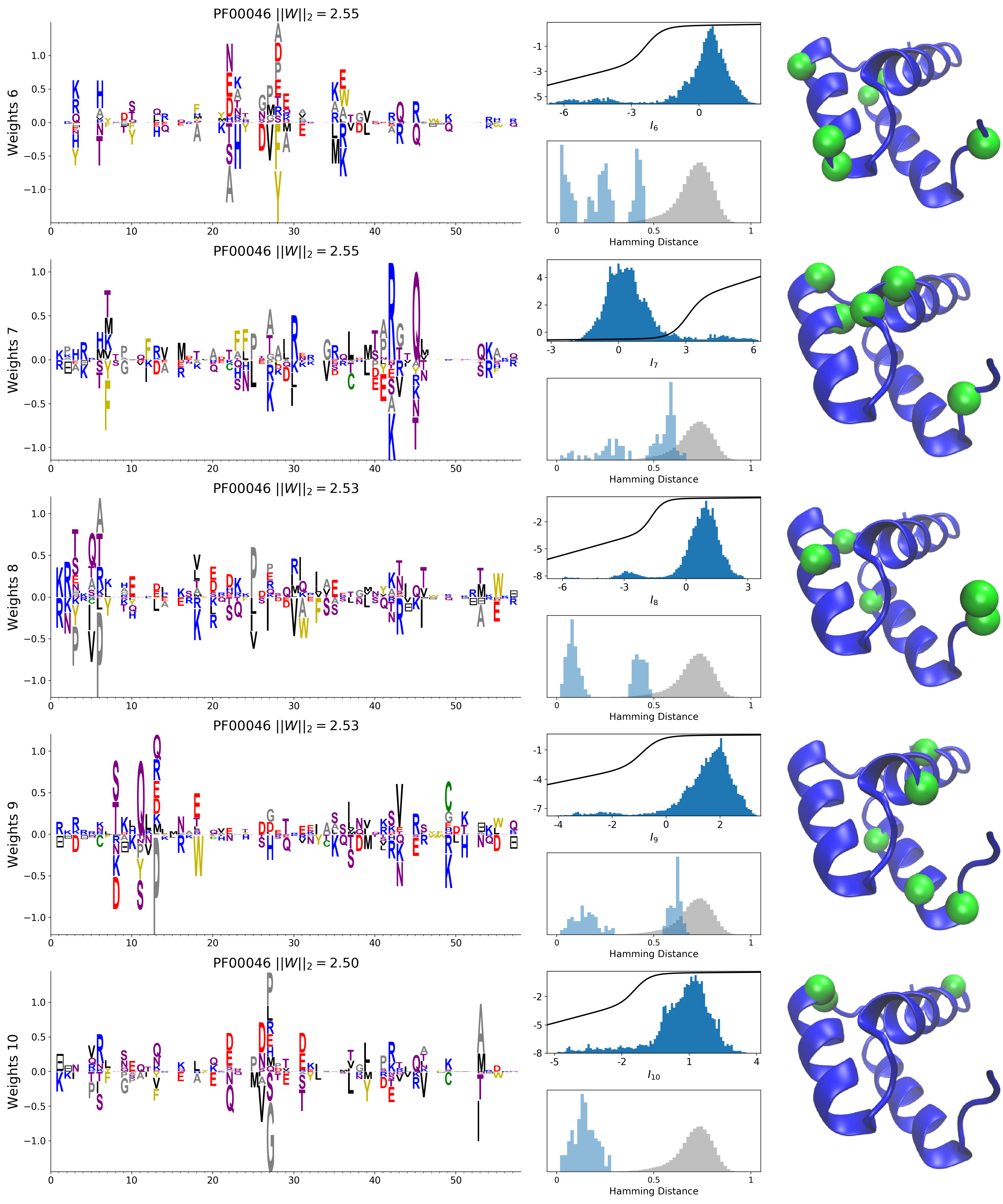

Supplement: Supplementary file 6. [file elife-39397-supp6.zip › Top_Sparse_features_all/PF00046_top_sparse_features.pdf]

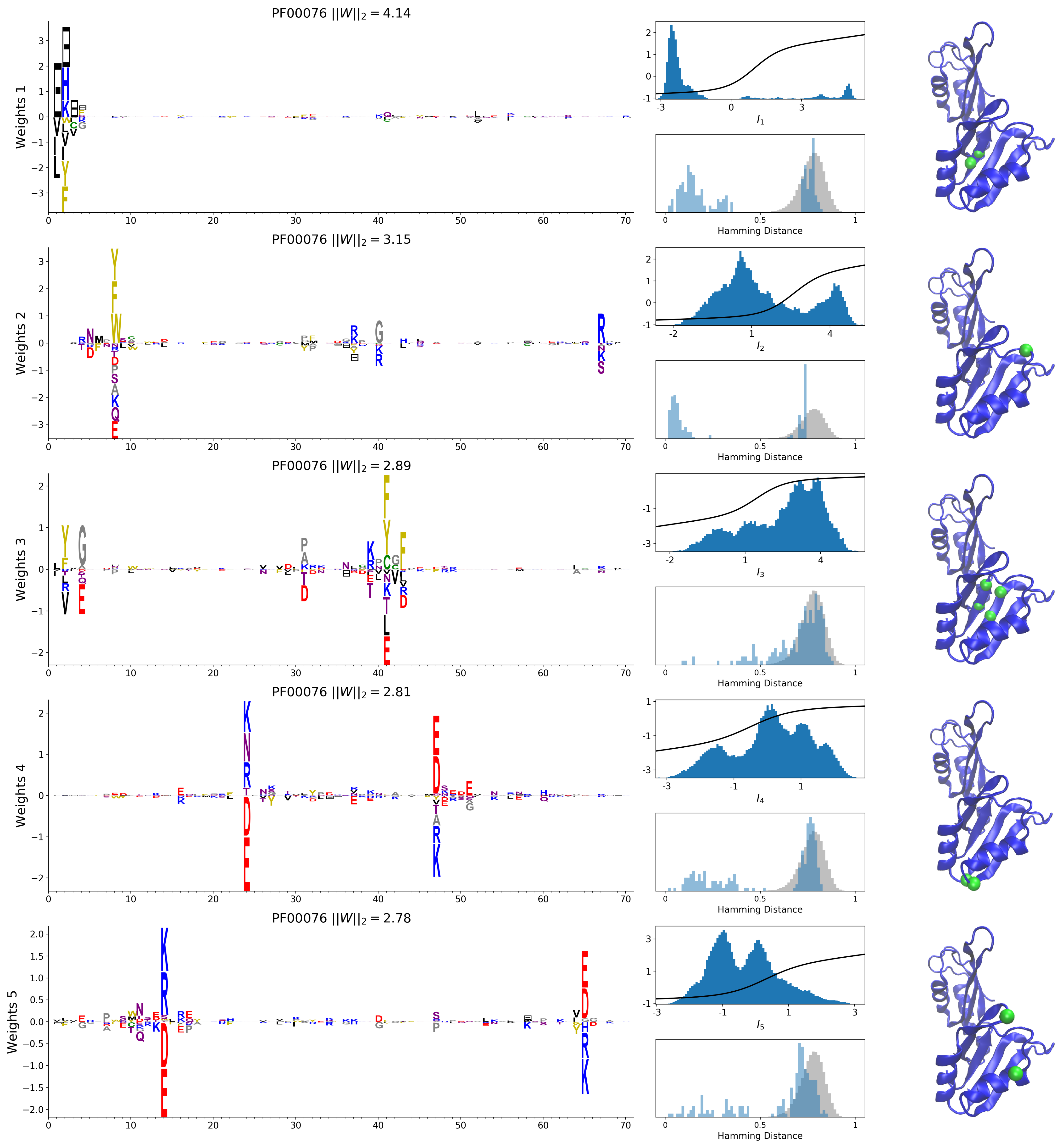

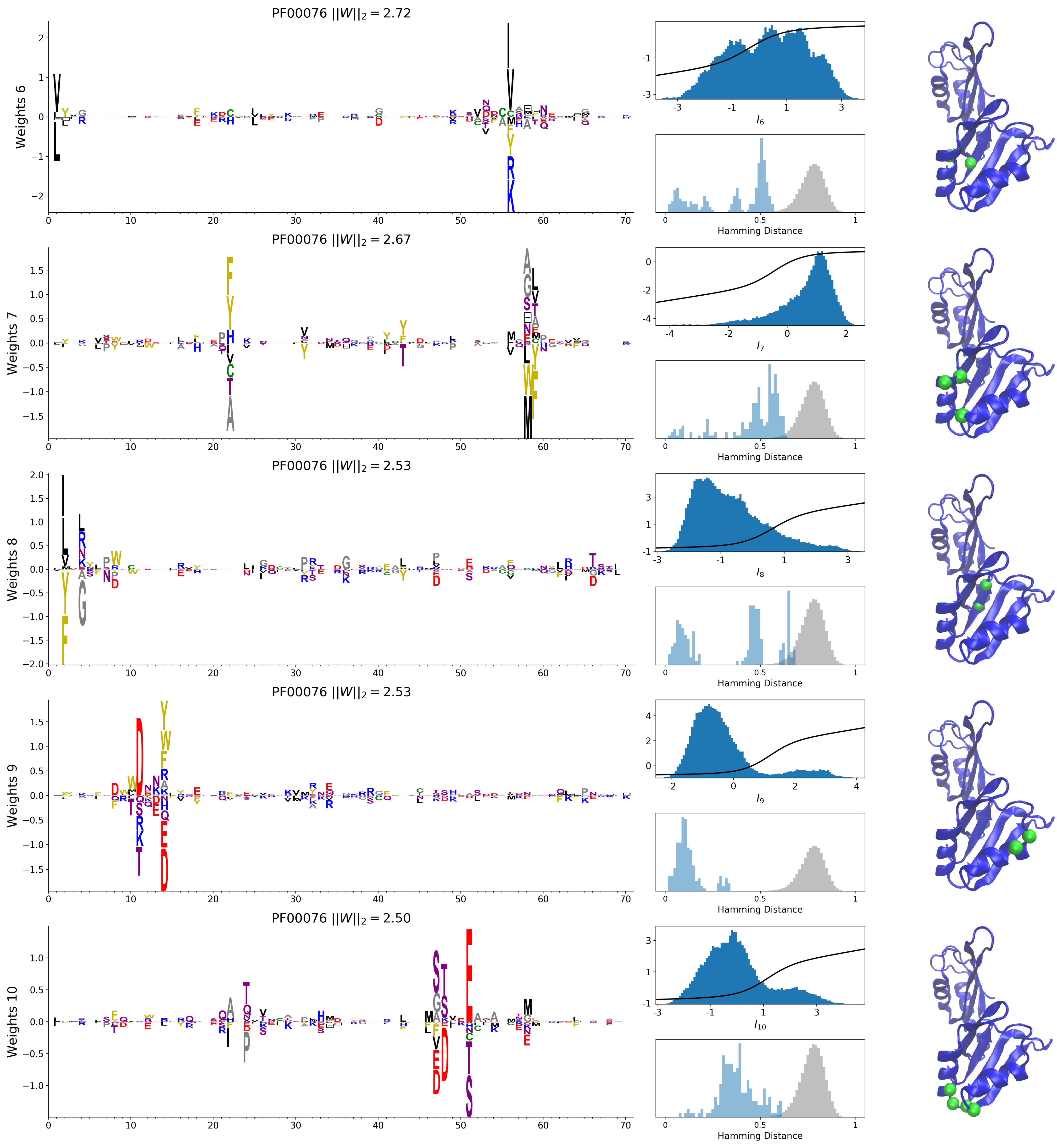

Supplement: Supplementary file 6. [file elife-39397-supp6.zip › Top_Sparse_features_all/PF00076_top_sparse_features.pdf]

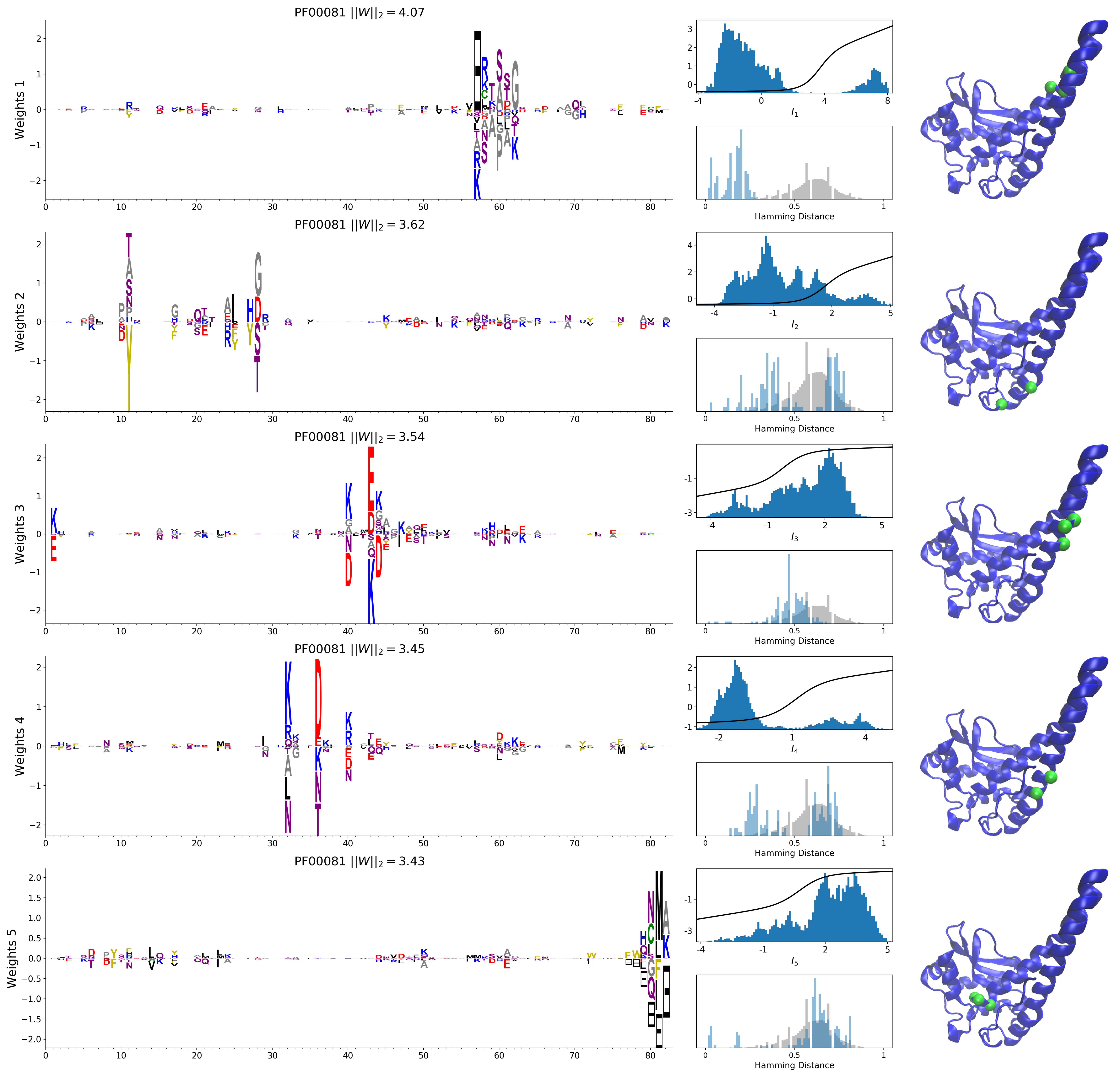

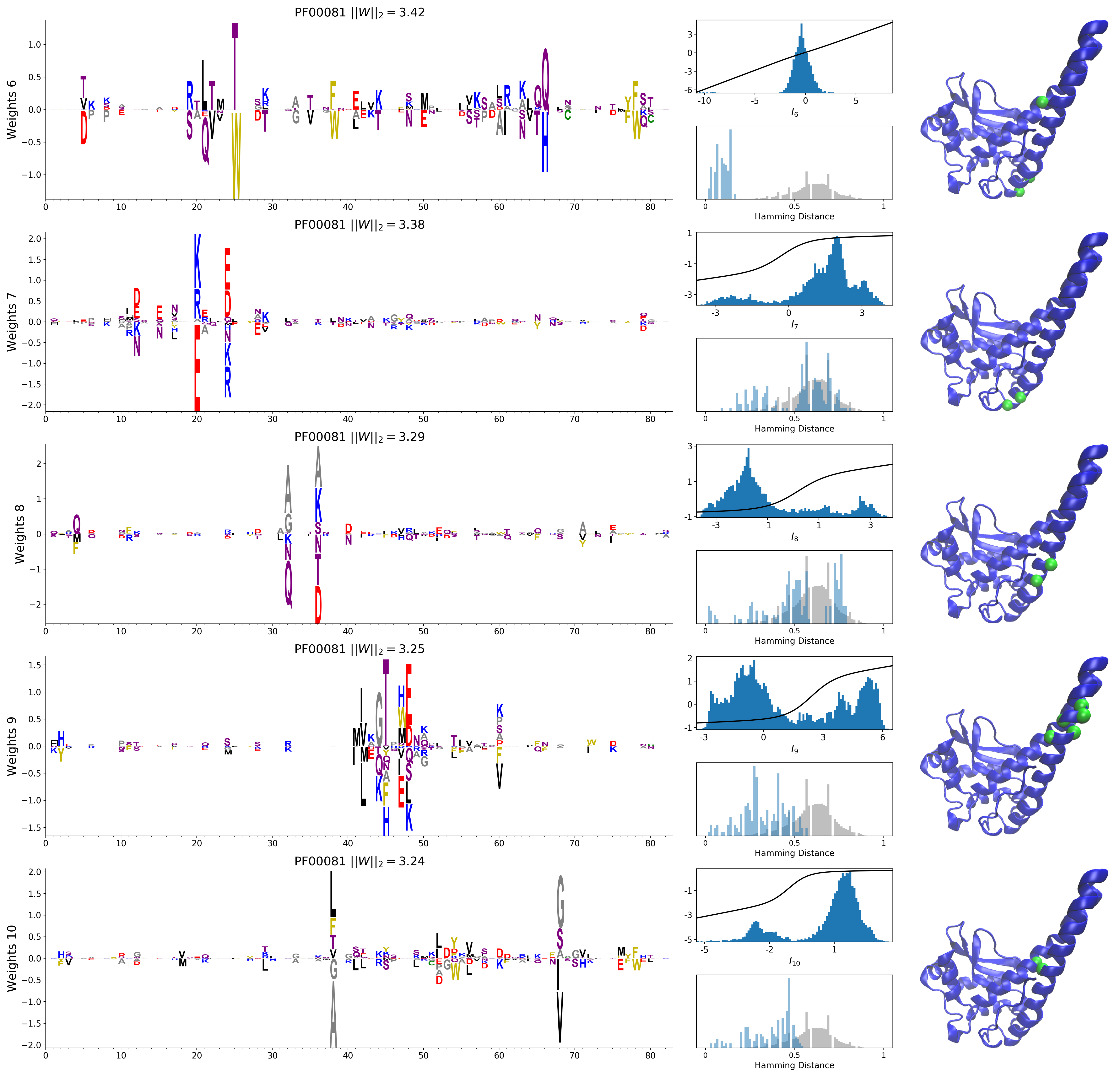

Supplement: Supplementary file 6. [file elife-39397-supp6.zip › Top_Sparse_features_all/PF00081_top_sparse_features.pdf]

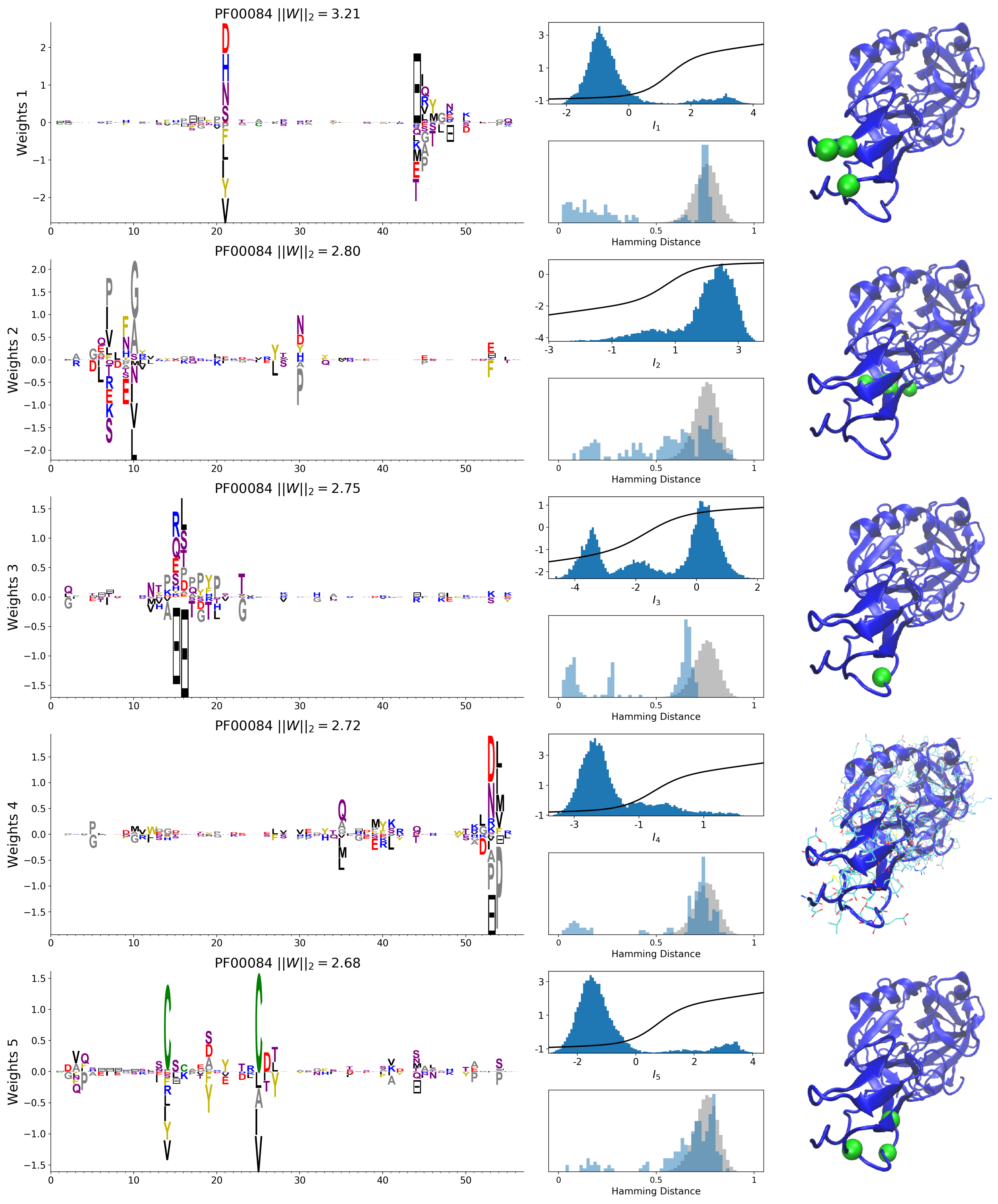

Supplement: Supplementary file 6. [file elife-39397-supp6.zip › Top_Sparse_features_all/PF00084_top_sparse_features.pdf]

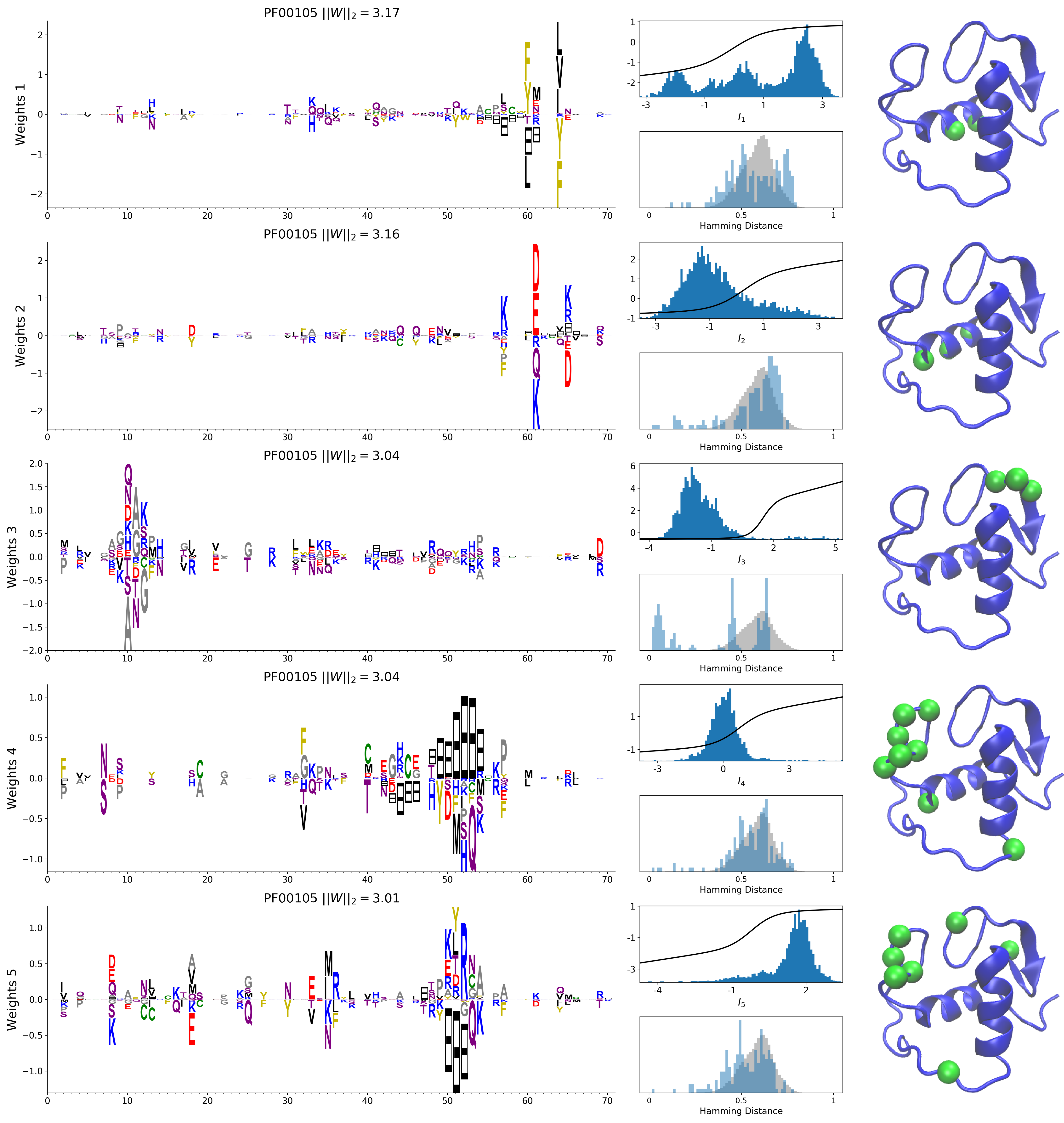

Supplement: Supplementary file 6. [file elife-39397-supp6.zip › Top_Sparse_features_all/PF00105_top_sparse_features.pdf]

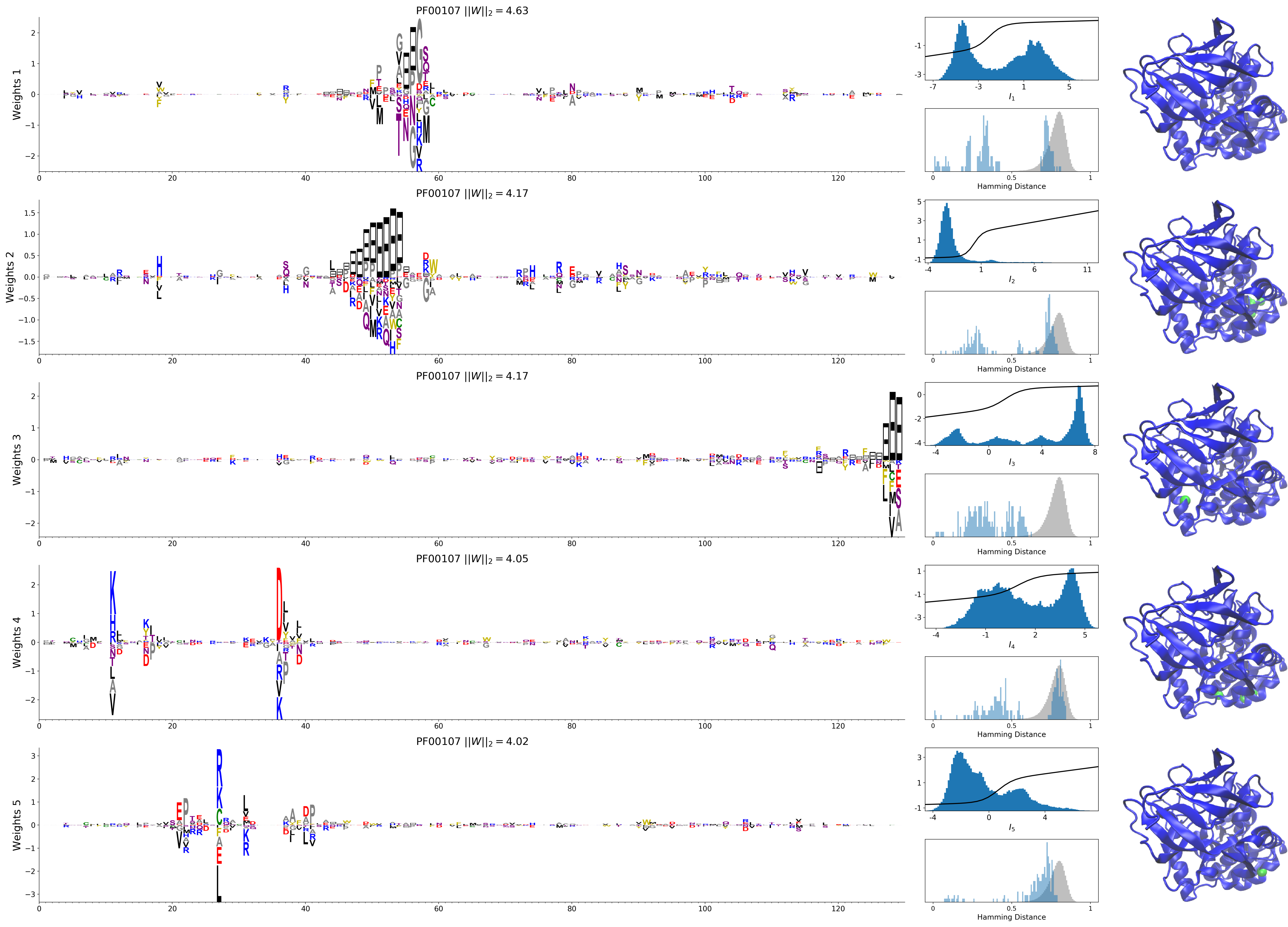

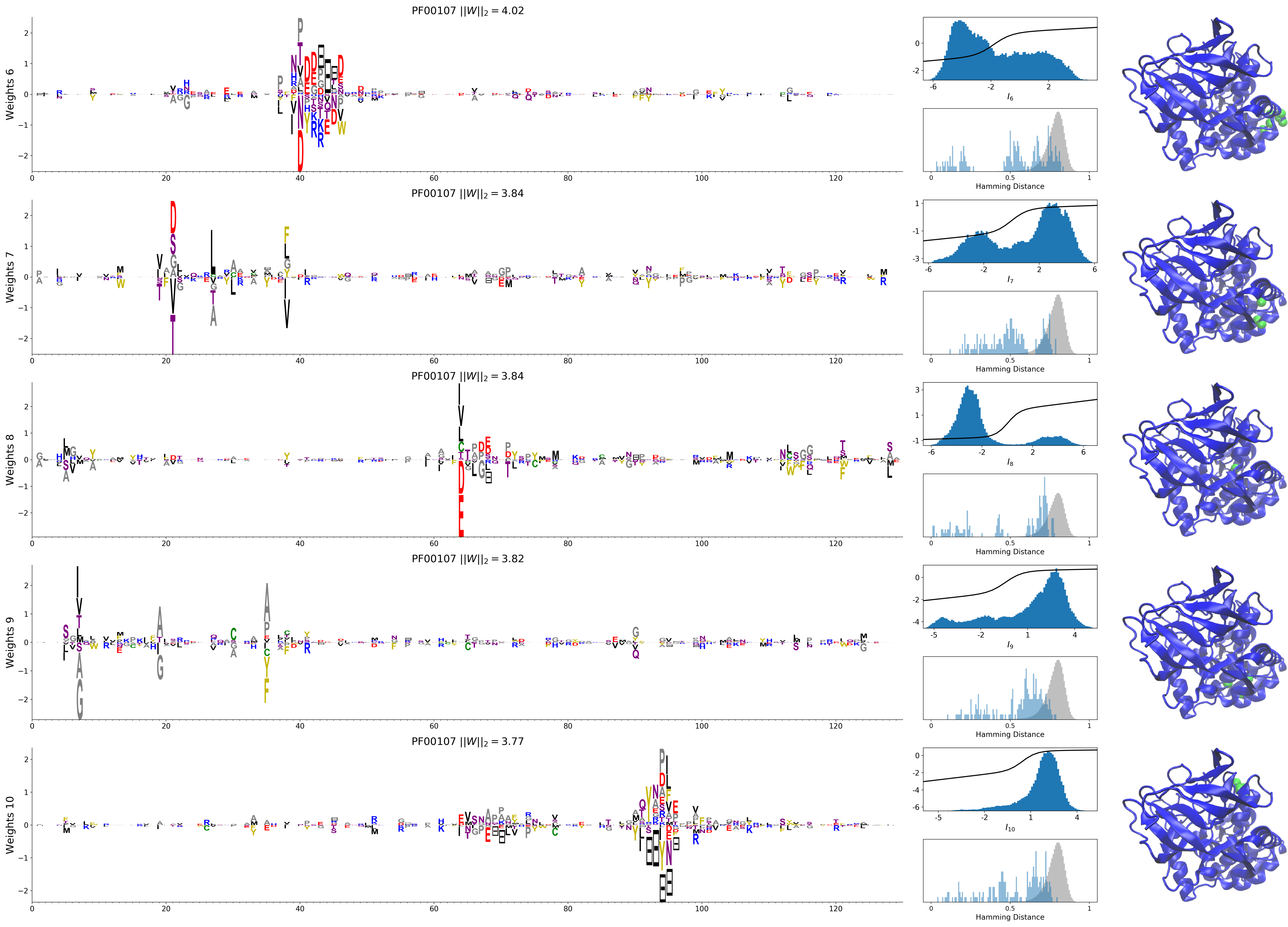

Supplement: Supplementary file 6. [file elife-39397-supp6.zip › Top_Sparse_features_all/PF00107_top_sparse_features.pdf]

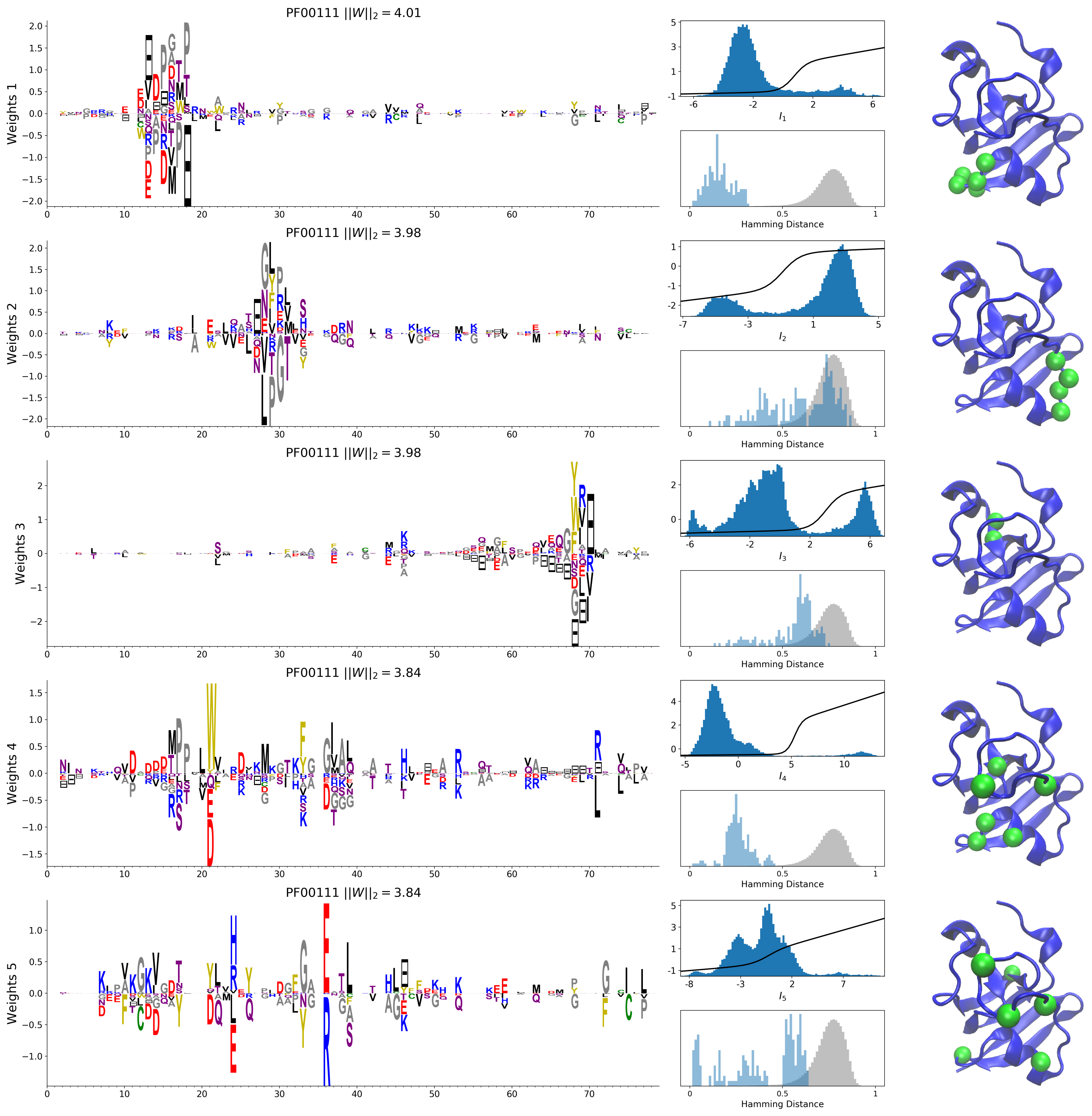

Supplement: Supplementary file 6. [file elife-39397-supp6.zip › Top_Sparse_features_all/PF00111_top_sparse_features.pdf]
