# Supplementary material for: Learning protein constitutive motifs from sequence data
Source: eLife. 2019 Mar 12;8:e39397. doi: 10.7554/eLife.39397 (PMC6436896; doi:10.7554/eLife.39397)
Supplement: Supplementary file 4. — The format is the same as that used for Appendix 1—figures 14–16. [file elife-39397-supp4.pdf]

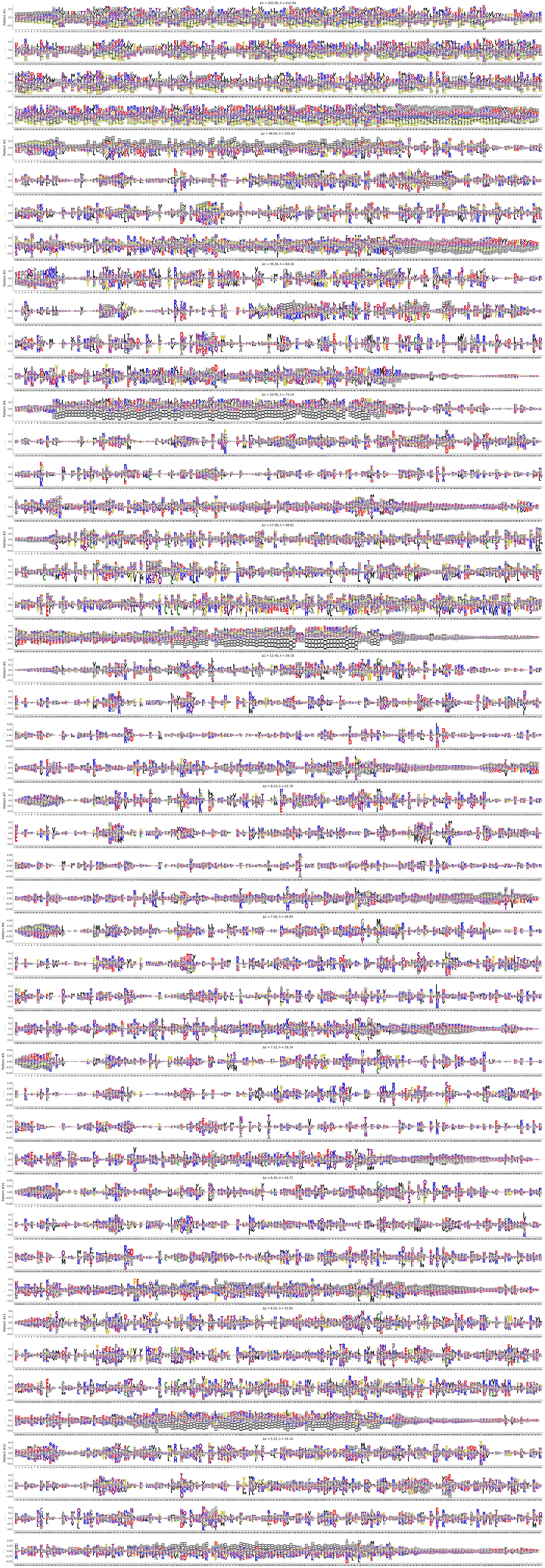
Pattern #1
Δε = 202.96, λ = 412.94
Pattern #2
Δε = 48.40, λ = 102.42
Pattern #3
Δε = 39.36, λ = 84.16
Pattern #4
Δε = 33.92, λ = 73.14
Pattern #5
Δε = 17.08, λ = 38.81
Pattern #6
Δε = 12.40, λ = 29.18
Pattern #7
Δε = 9.33, λ = 22.79
Pattern #8
Δε = 7.50, λ = 18.93
Pattern #9
Δε = 7.12, λ = 18.14
Pattern #10
Δε = 6.45, λ = 16.72
Pattern #11
Δε = 6.02, λ = 15.81
Pattern #12
Δε = 5.22, λ = 14.10
