# Supplementary material for: Learning protein constitutive motifs from sequence data
Source: eLife. 2019 Mar 12;8:e39397. doi: 10.7554/eLife.39397 (PMC6436896; doi:10.7554/eLife.39397)
Supplement: Supplementary file 5. [file PF00111_top_features.pdf › Top_features_all/PF00111_top_features.pdf]

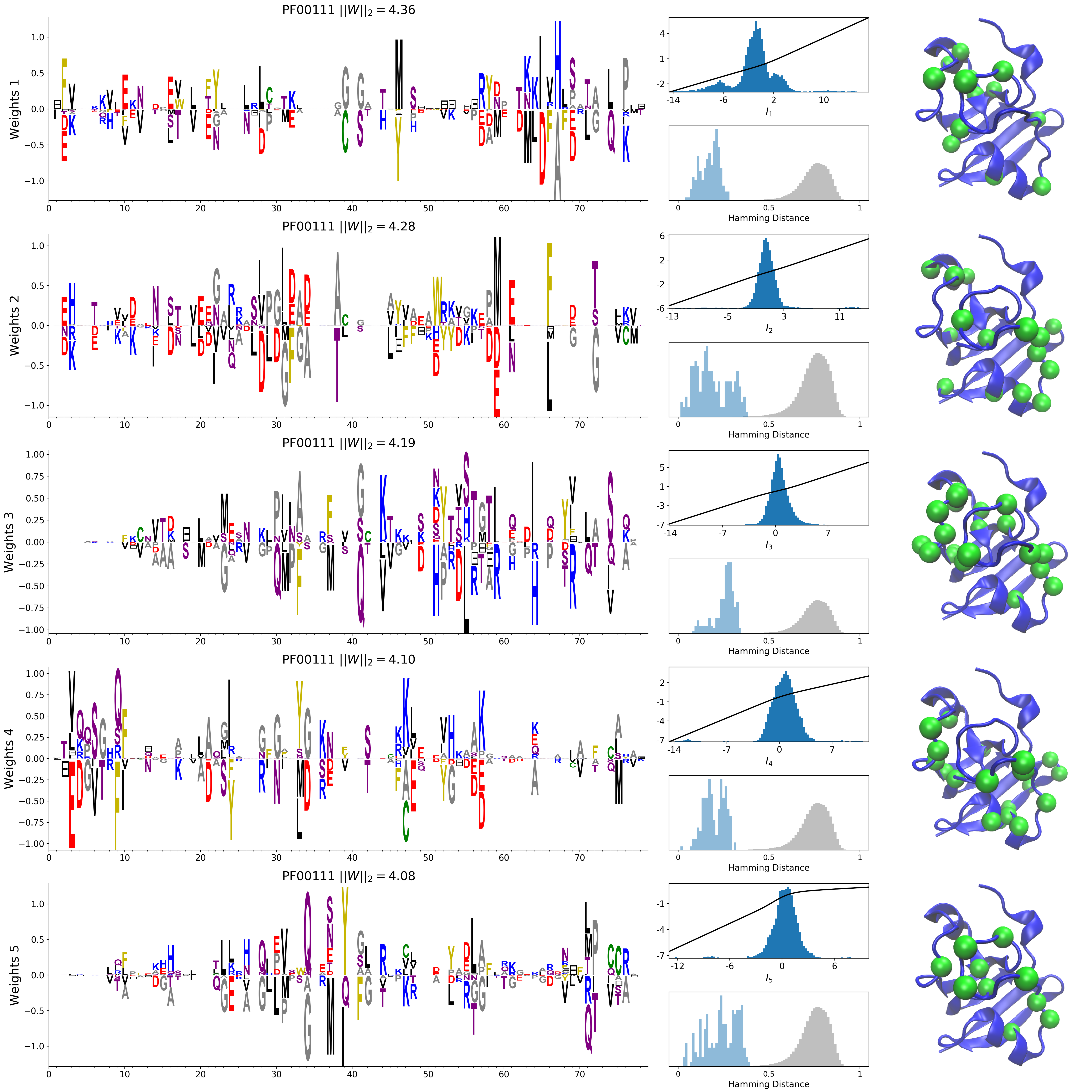
PF00111 $||W||_2 = 4.36$
Weights 1
I_1
Hamming Distance
PF00111 $||W||_2 = 4.28$
Weights 2
I_2
Hamming Distance
PF00111 $||W||_2 = 4.19$
Weights 3
I_3
Hamming Distance
PF00111 $||W||_2 = 4.10$
Weights 4
I_4
Hamming Distance
PF00111 $||W||_2 = 4.08$
Weights 5
I_5
Hamming Distance

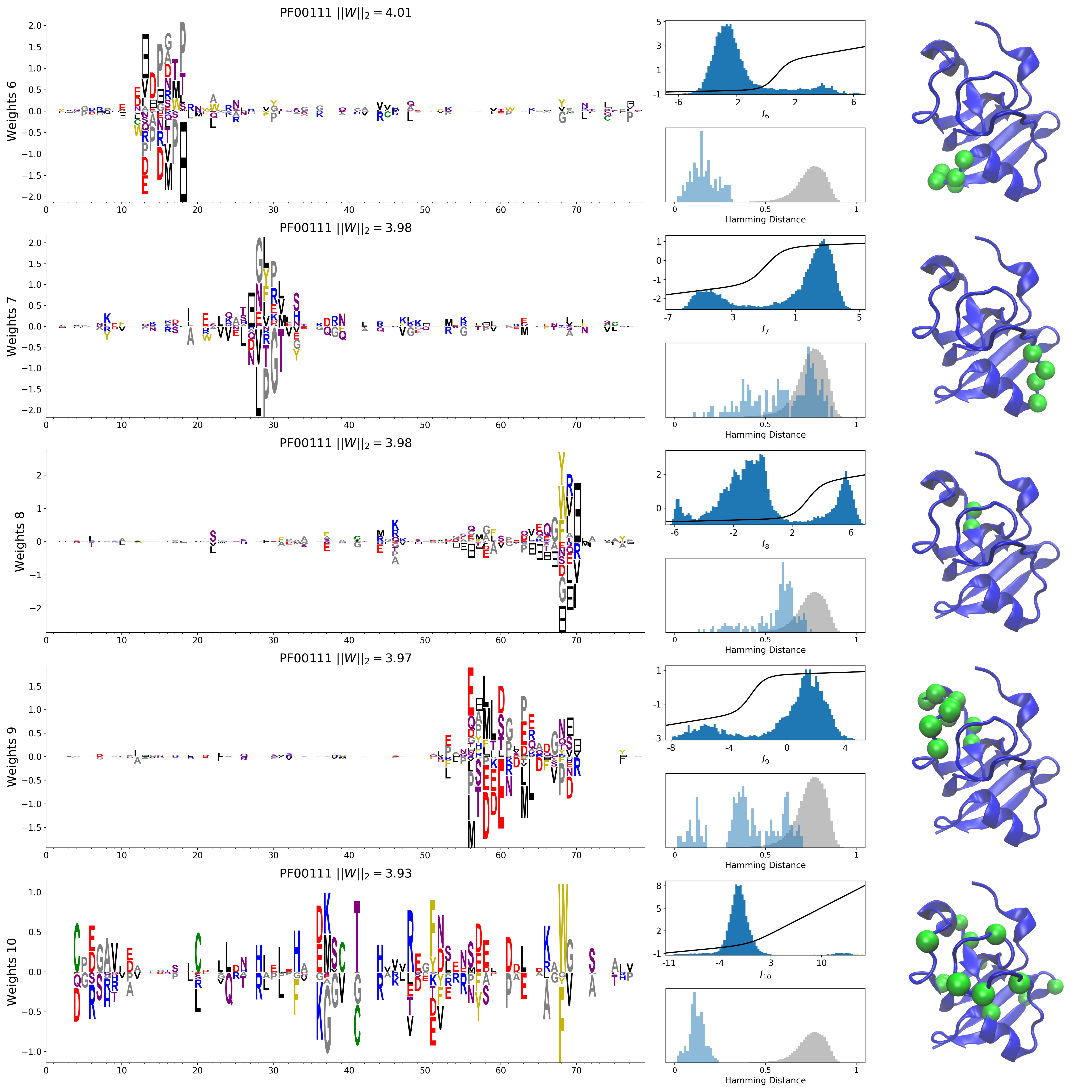

PF00111 $||W||_2 = 4.01$
Weights 6
I_6
Hamming Distance
PF00111 $||W||_2 = 3.98$
Weights 7
I_7
Hamming Distance
PF00111 $||W||_2 = 3.98$
Weights 8
I_8
Hamming Distance
PF00111 $||W||_2 = 3.97$
Weights 9
I_9
Hamming Distance
PF00111 $||W||_2 = 3.93$
Weights 10
I_{10}
Hamming Distance
